# Understanding the Effect of M(III) Choice in Heterodinuclear Polymerization Catalysts

**DOI:** 10.1021/acs.inorgchem.4c04430

**Published:** 2024-11-19

**Authors:** Katharina H.S. Eisenhardt, Francesca Fiorentini, Charlotte K. Williams

**Affiliations:** Department Chemistry, University of Oxford, Chemistry Research Laboratory, 12 Mansfield Road, Oxford, OX1 3TA U.K.

## Abstract

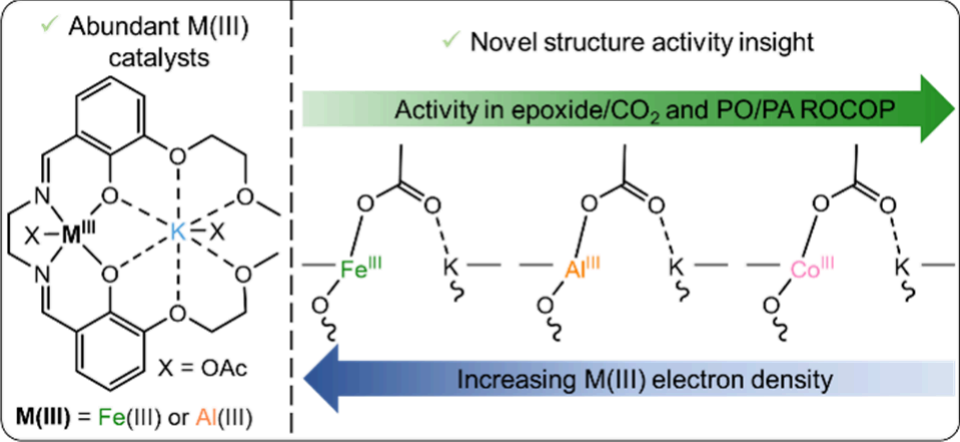

The ring-opening copolymerization (ROCOP) of epoxides with CO_2_ or anhydrides is a promising strategy to produce sustainable polycarbonates and polyesters. Currently, most catalysts are reliant on scarce and expensive cobalt as the active center, while more abundant aluminum and iron catalysts often suffer from lower activities. Here, two novel heterodinuclear catalysts, featuring abundant Al(III), Fe(III), and K(I) active centers, are synthesized, and their performance in the polymerization of four different monomer combinations is compared to that of their Co(III) analogue. The novel Al(III)K(I) catalyst exhibits outstanding activities in the cyclohexane oxide (CHO)/CO_2_ ROCOP, and at 1 bar CO_2_ pressure it is the fastest aluminum-based catalyst reported to date. The M(III) site electronics for all three catalysts, Al(III)K(I), Fe(III)K(I), and Co(III)K(I), are measured using IR and NMR spectroscopy, cyclic voltammetry, and single crystal X-ray diffraction. A correlation between M(III) electron density and catalytic activity is revealed and, based on the established structure–activity relationship, recommendations for the future catalyst design of abundant Al(III)- and Fe(III)-based catalysts are made. The catalytic performances of both Al(III)K(I) and Fe(III)K(I) are further contextualized against the relative elemental abundance and cost. On the balance of performance, abundance, and cost, the Al(III)K(I) complex is the better catalyst for the carbon dioxide/epoxide ROCOP, while Fe(III)K(I) is preferable for anhydride/epoxide ROCOP.

## Abstract 2

Two novel catalysts containing abundant Fe(III), Al(III), and K(I) active centers are synthesized and their performance in the polymerization of four different monomer combinations is compared to their Co(III) analogue. In the cyclohexane oxide/CO_2_ ROCOP at 1 bar CO_2_ pressure, the novel Al(III)K(I) catalyst is the fastest aluminum-based catalyst reported to date. The M(III) site electronics for all three catalysts are measured, and a correlation between M(III) electron density and catalytic activity is established.

## Introduction

The current linear plastic production needs to move toward a circular plastic economy in order to reduce waste and greenhouse gas emissions.^1^ The ring-opening copolymerization (ROCOP) of epoxides with CO_2_ or anhydrides furnishes polycarbonates or polyesters and is a promising strategy since it applies waste or bioderived monomers to make recyclable polymers, some of which are also degradable.^2−8^ Over the past two decades, significant advances in epoxide/heterocumulene ROCOP catalysis have occurred, with leading catalysts reaching impressive activities and selectivity even for “challenging” monomers, such as propene oxide (PO) and CO_2_.^4,9−11^ The majority of these highly active and selective catalysts rely on cobalt(III) active sites.^4,9,12−16^ One concern is that cobalt is not very Earth-abundant (crustal abundance: 27 ppm), is located in specific geographies, and is a key element in many battery configurations. These factors resulted in its classification as a “critical mineral” by the UK parliament in 2023.^17^

Considering the low abundance and high global demand for cobalt, it may be desirable to replace it in catalysis, especially with more abundant metals, such as aluminum (crustal abundance: 84000 ppm) and iron (crustal abundance: 52000 ppm).^18^ There is little precedent in this field of polymerization catalysts for these metals, and where they were applied the resulting catalysts usually showed significantly lower activity and selectivity compared to cobalt catalysts.^19−21^ A further consideration is that many of these Fe(III) and Al(III) catalysts are applied with at least an equivalent of cocatalysts, preferably a phophoniminium halide (PPNX, X = Cl, Br, I) (Figure S1 and Figure S2).^19−22^ The cocatalyst tends to be expensive and heavy, and the starting elements in these salts (P, N, halogens) also have environmental impacts.^23−25^

**Figure 1:**
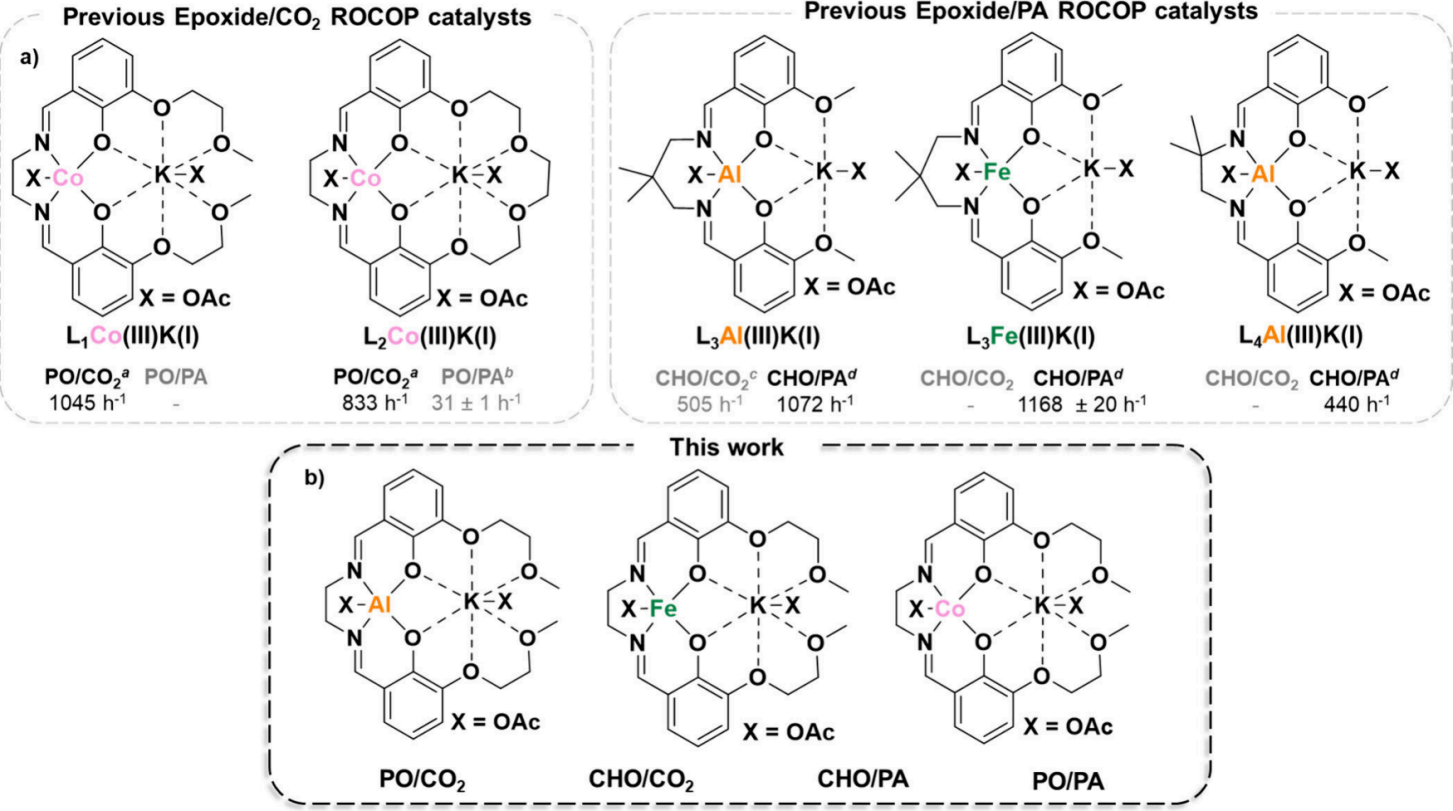
(a) Previously reported heterodinuclear polymerization catalysts. Four different ligands (**L1–L4**) have been employed with three different M(III) centers, where M(III) = Co(III), Al(III), or Fe(III), reaction conditions: ^*a*^1:20:4000 [catalyst]/[CTA]/[PO], 70 °C, 20 bar;^16,37^^*b*^1:100:1000 [catalyst]/[PA]/[PO], 50 °C;^38^^*c*^1:2000 [catalyst]/[CHO], 100 °C, 20 bar;^32,39,40^^*d*^1:400:2000 [catalyst]/[PA]/[CHO], 100 °C.^32,39^ (b) Systematic investigation of the effect of M(III) choice on four different polymerization reactions conducted in this work.

Considering the reported Al(III) or Fe(III) catalysts in carbon dioxide/epoxide ROCOP, the most active is a bifunctional Al(III) porphyrin catalyst, reported by Nozaki and co-workers, in which the cocatalyst, with four quaternary ammonium halides per Al(III), is covalently tethered to the porphyrin ligand. It achieved a very impressive activity (turn over frequency, TOF) of 10 000 h^–1^ and tolerated low loadings using cyclohexene oxide (1:40 000, catalyst/cyclohexane oxide (CHO), 120 °C).^26^ For CHO/CO_2_ ROCOP, we reported a heterodinuclear Al(III)K(I) catalyst, coordinated by a Schiff base ligand, which operated without any cocatalyst and reached TOF = 505 h^–1^ (1:2000, catalyst/CHO, 100 °C) but suffered from a low polymer selectivity of 88% (Figure 1).^27^ In terms of iron catalysts, Kleij and co-workers reported an Fe(III) amino triphenolate catalyst system (with 10 equiv of PPNCl), which achieved TOF = 63 h^–1^ for CHO/CO_2_ ROCOP but was applied with supercritical CO_2_ pressures ([Fe]/[PPNCl]/[CHO] = 1:10:200, 80 bar CO_2_, 85 °C).^28^ Recently, we reported a heterodinuclear Fe(II)Mg(II) catalyst, coordinated by a macrocyclic ligand, that operated without a cocatalyst and achieved an activity of 1071 h^–1^ at just 1 bar carbon dioxide pressure ([cat]_0_/[CHO]_0_ = 1:4000, 1 bar CO_2_ 100 °C).^29^ There are a few aluminum- or iron-based catalysts for PO/CO_2_ ROCOP but all require a cocatalyst.^30,31^

For epoxide/anhydride ROCOP, there are only a few iron catalysts. We recently reported an Fe(III)K(I) catalyst, which operates without a cocatalyst, that showed an activity of 4800 h^–1^.^32^ The other Fe catalysts exhibited much lower rates (TOF < 10 h^–1^) and usually require PPNX salts.^32−36^

In contrast, there are already some excellent aluminum catalysts. Coates and co-workers reported an aluminum complex, coordinated by a Schiff base ligand incorporating aminocyclopropenium halide, showing a very good TOF of 93 h^–1^ using the generally slower monomer PO (60 °C, 1:400:2000, [catalyst]/[CPMA]/[PO]).^41^ Using CHO, which is usually polymerized both hotter and faster than PO, we reported two Al(III)K(I) catalysts coordinated by Schiff base ligands, which exhibited TOFs of 1072 and 1070 h^–1^ (100 °C, [cat]_0_/[phthalic anhydride]_0_/[CHO]_0_ = 1:400:4000, Figure 1a (L_3_ and L_4_, respectively)).^27,40^ However, when the Al(III)K(I) catalysts were used for PO/phthalic anhydride (PA) ROCOP, lower TOFs of 20 and 26 h^–1^, respectively, were observed (60 °C, [cat]_0_/[PA]_0_/[CHO]_0_ = 1:400:4000).^27,40^ Overall, there is some precedent for high-performing aluminum- and iron-based epoxide/anhydride ROCOP catalysts; however, there remains currently only one aluminum- or iron-based epoxide/CO_2_ catalyst that exhibits a performance comparable to that reported for cobalt based catalysts.

In an attempt to overcome the current gap in Al(III) and Fe(III) epoxide/CO_2_ ROCOP catalysts, we initially reviewed heterodinuclear catalysts recently reported by our group as cobalt-based heterodinuclear catalysts utilizing two different ligand frameworks **L1** and **L2**, which have shown outstanding rates for both CHO/CO_2_ and PO/CO_2_ ROCOP (Figure 1).^16,37^ In contrast, heterodinucelar catalysts with Al(III)K(I) and Fe(III)K(I) bound in ligand frameworks **L3** or **L4** showed poor activity or no activity in the epoxide/CO_2_ ROCOP but exhibited high activities in the CHO/PA ROCOP (Figure 1, TOF > 1000 h^–1^).^27,32,40^

Our aim is to investigate whether we could rationally design a new Al(III) or Fe(III) catalyst for epoxide/CO_2_ ROCOP by employing ligands such as **L1** and **L2**, which previously showed excellent activities when used with Co(III). Our approach is to compare, under standardized and equivalent conditions, the performance of a series of heterodinuclear polymerization catalysts with Al(III), Fe(III), and Co(III) in the same ligand framework. The s-block metal is fixed as K(I) since it generally shows the highest rates and selectivity of group 1 congeners and has the benefit of being Earth-abundant (crustal abundance: 22 800 ppm) and inexpensive.^16,18,37−40^ As the ligand framework, we chose the recently developed **L1** (Figure 1b) since its Co(III)K(I) complex showed unparalleled performance in CO_2_/epoxide ROCOP.^16^

We also seek insight into the structure–performance relationships, focusing on the interplay of M(III) and ligand choice in the polymerization of different monomer combinations. All previous studies have focused on one M(III) center (Al(III), Fe(III), or Co(III)) for one monomer combination only, but it is apparent that not all catalysts perform the same for different monomer combinations (Figure 1).^27,38,38,40^ Thus, the two novel Al(III)K(I) and Fe(III)K(I) complexes will be evaluated against the performance of the previously reported Co(III)K(I) heterodinuclear catalysts (**L1**Co(III)K(I)) (Figure 1) for both epoxide/CO_2_ and epoxide/anhydride ROCOP.

## Results and Discussion

### Catalyst Synthesis and Characterization

**Figure 2:**
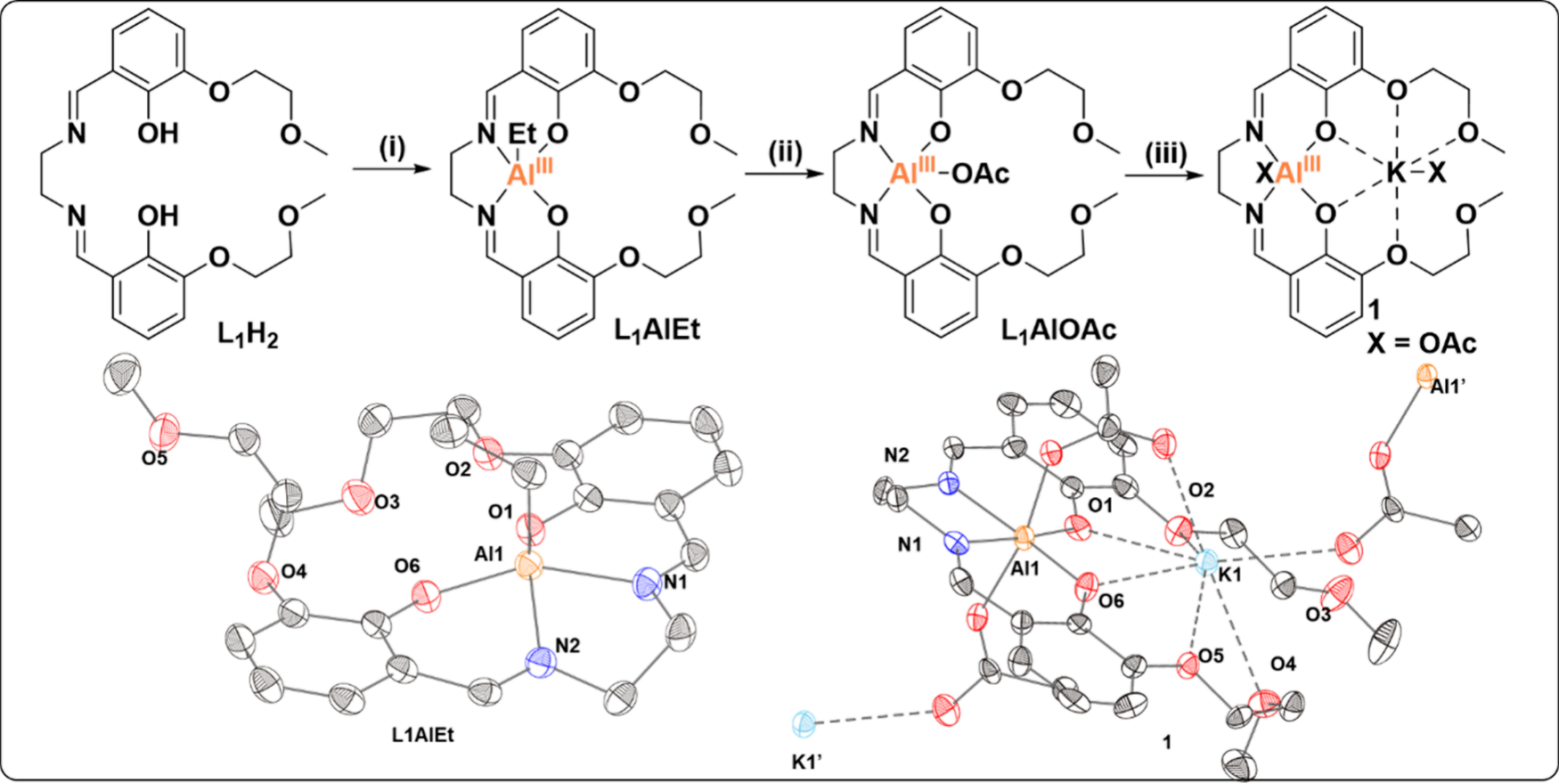
Synthesis of the Al(III)K(I) complex (**1**): (i) 1.5 equiv of AlEt_3_ in 2-MeTHF, −30 °C, then warm up to 25 °C, N_2_; (ii) AcOH, in 2-MeTHF, N_2_, for 16 h; (iii) 1 equiv of KOAc in 2-MeTHF, at 70 °C for 2 h, then 45 °C for 16 h. The solid-state structures of all complexes obtained by single crystal XRD are shown. Residual solvents and H atoms are omitted for clarity. Thermal ellipsoids are shown at a probability of 50% (Tables S7–9).

**Figure 3:**
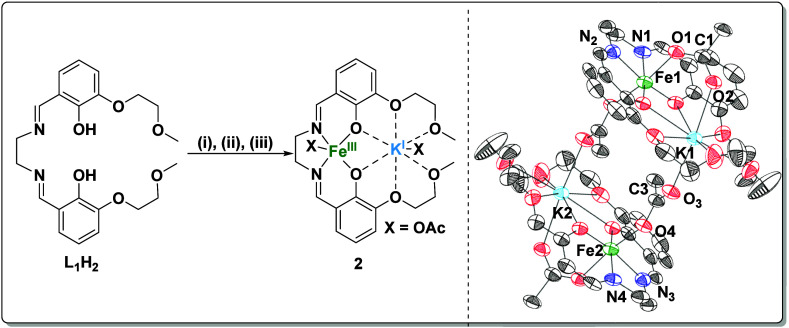
Synthesis of Fe(III)K(I) complex (**2**): (i) Fe(OAc)_2_, 2-MeTHF, N_2_, 2 h; (ii) air, 16 h, 2 equiv of AcOH; (iii) KOAc, 16 h. The solid-state structure obtained by single crystal XRD is shown. Residual solvents and H atoms are omitted for clarity. Thermal ellipsoids are shown at a probability of 30% (Tables S7–9).

The pro-ligand **H**_**2**_**L**_**1**_ features two distinctive metal coordination environments: a Schiff base binding pocket for M(III) and a K(I) binding pocket, featuring two ether “arms” (Figure 1). **H**_**2**_**L**_**1**_ was synthesized in 90% yield, following the reported procedure, by the reaction between benzaldehyde and ethylenediamine in methanol (Figure S3 and Figure S4).^16^ New complexes (**1** and **2**) were synthesized by **H**_**2**_**L**_**1**_ metalation (Figure 2 and 3). Initially, the Al(III)K(I) complex (**1**) was synthesized in a stepwise procedure from **H**_**2**_**L**_**1**_, AlEt_3_, and KOAc (Figure 2). Following routes to other Al(III)M(I) catalysts, the first synthesis was performed by using tetrahydrofuran (THF) and dichloromethane (DCM) as solvents. A solution of **H**_**2**_**L**_**1**_ and a solution of AlEt_3_ in THF were cooled to −30 °C, combined under an inert N_2_ atmosphere while cold, stirred, and slowly warmed to room temperature. The novel **L**_**1**_**AlEt** complex was isolated in >95% yield and fully characterized using NMR spectroscopy (Figures S5–9), infrared spectroscopy (IR) (Figure S10), and single crystal X-ray diffractometry (XRD, Figure 2). The NMR data show the disappearance of the pro-ligand phenol resonance (∼11 ppm) after the formation of **L**_**1**_**AlEt**, as well as the new Al-ethyl peak (−0.5 ppm) (Figures S3 and Figure S5).

The X-ray diffraction data further confirm the complex connectivity, with the aluminum center in a square pyramidal conformation (Figure 2). Addition of acetic acid to a stirred solution of **L**_**1**_**AlEt**, in THF under an inert N_2_ atmosphere, led to the formation of **L**_**1**_**AlOAc**, which was isolated in 90% yield. Complex formation was monitored by ^1^H NMR spectroscopy; the spectrum for **L**_**1**_**AlOAc** showed the disappearance of the Al-ethyl signals and the appearance of an acetate signal at 1.6 ppm (Figure S5 and Figure S11). Subsequently, complex **1** was synthesized from **L**_**1**_**AlOAc** by the addition of KOAc to a stirred solution of **L**_**1**_**AlOAc**, in DCM, and characterized by NMR spectroscopy (Figures S16–S20), IR spectroscopy (Figure S10), and single crystal XRD. The coordination of KOAc into the ether binding pocket of **L**_**1**_**AlOAc** was confirmed by changes to resonances for the ether protons in the ^1^H NMR spectrum (Figure S16). Single crystal XRD confirmed the expected connectivity, with Al(III) coordinated by the Schiff base donors and K(I) coordinated by the ethers. The aluminum center adopts an octahedral geometry and coordinates two acetate ligands. One acetate bridges the two metals within the same molecule. The second acetate ligand bridges from the aluminum center to a potassium metal (K1′) coordinated in a second molecule of ligand (Figure 2). Interestingly, in the solid-state structure of (**1**), one ether oxygen (O3) does not coordinate to K(I). The solution-state NMR spectra of **1** show two signals for the C2 ether linkages, indicative of either a high-symmetry coordination environment around K(I) with all ether oxygens coordinated or a fluxional process of coordination and decoordination of the ether “arms”, faster than the NMR time scale. In contrast to the observed polymeric solid-state structure, solution-state nuclearity was confirmed as monomeric using diffusion order spectroscopy (DOSY in CDCl_3_, Figure S21). The purity of **L**_**1**_**AlOAc** and **1** was confirmed by elemental analyses.

Fe(III)K(I) (**2**) was also synthesized by the metalation of **H**_**2**_**L**_**1**_, in THF (Figure 3).^32^ Fe(II)OAc_2_ was added to a stirred solution of **H**_**2**_**L**_**1**__,_ in THF, under an inert atmosphere. After 2 h, the reaction was opened to air, and two equivalents of acetic acid were added in order to oxidize the Fe(II) center. The Fe(III)K(I) complex was formed by the addition of KOAc, which was subsequently stirred for 16 h.

Complex **2** was isolated in 73% yield and fully characterized using IR spectroscopy (Figure S22), Super Conducting Quantum Interference Device (SQUID) magnetometry (Figure S23, Table S1), XRD (Figure 3) and cyclic voltammetry (CV) (Figure S24 and Figure S25). The solid-state structure of **2** confirms it is a heterodinuclear complex, with the iron center coordinated by the Schiff base donors and the potassium coordinated by the ethers (Figure 3). Fe(III)K(I) is dimeric in the solid state, and each Fe(III) center exhibits an octahedral geometry and coordinates two acetate ligands. One acetate bridges between the iron and the potassium center bound within the same ligand. The second acetate bridges between the iron center and a second potassium, bound in a second molecule of ligand (Figure 3). The oxygen–carbon bonds in the second acetate show two different bond lengths (i.e., O3–C3 = 1.231(5) vs O4–C3 = 1.282(4)), indicating the complex adopts a ferrate structure. Both CV and SQUID magnetometry support the successful oxidation from Fe(II) to Fe(III) through the presence of an Fe(III)/II) reduction in the first cyclic voltammogram and an observed μ_eff_ of 5.46. These data are consistent with the complex having a high-spin d^5^ Fe(III) center (Figure S23, Table S1). The bulk purity of **2** was confirmed by elemental analysis. The Co(III)K(I) complex (**3**) was synthesized in 75% yield following the reported literature procedure.^16^ It was characterized by multinuclear NMR spectroscopy, cyclic voltammetry, and elemental analysis (Figures S26–28).

Conventional solvents, especially chlorinated solvents as used in the initial syntheses of Al(III)K(I) and Fe(III)K(I), have environmental, health and safety, and disposal concerns.^42−44^ It is, therefore, desirable to replace the THF and DCM used in the catalyst syntheses.^44^ 2-MeTHF has been identified as a greener replacement for these solvents.^44^ In the synthesis of both novel complexes (**1** and **2**), THF was successfully replaced with 2-MeTHF (Figure 2, 3). Previously, chlorinated solvents were necessary in the synthesis of Al(III)K(I) complexes to overcome the low solubility of the monometallic Al(III) acetate complex in most hydrocarbon solvents.^27,40^

Indeed, at room temperature the Al(III) acetate complex is insoluble in 2-MeTHF; however, at 70 °C it showed sufficient solubility to perform the K(I) metalation reaction (Figure 2). The yield of Al(III)K(I) (**1**) was improved from 55% in THF to 80% in 2-MeTHF. The yield of Fe(III)K(I) (**2**) (73%) was unaffected by the change in the solvent. The successful formation of Al(III)K(I) (**1**), using the novel synthetic route, was confirmed by multinuclear NMR and IR spectroscopy (Figure S31). In the synthesis of Fe(III)K(I) (**2**), the successful formation of **2** was confirmed by comparison of the IR and cyclic voltammogram data to that obtained for catalyst (**2**) synthesized in THF (Figure S29 and Figure S30).

### Epoxide/CO_2_ and Epoxide/PA ROCOP Catalysis

The activity and selectivity of Al(III)K(I) (**1**) and Fe(III)K(I) (**2**) were tested for CHO/CO_2,_ PO/CO_2_, CHO/PA, and PO/PA ROCOP. In order be able to compare the activity and selectivity of catalysts **1**–**3**, all reactions were performed at the same catalyst:CTA:epoxide loading of 1:20:4000 at a CO_2_ pressure of 20 bar or PA loading of 1:400 ([cat]_0_/[PA]_0_). Compared to many previous reports, the low catalyst loading employed here renders these very demanding reaction conditions. A high tolerance to low catalyst loading is important, for example, as catalysts need to tolerate monomer impurities. All reactions were performed using 20 equiv of 1,2-trans-cyclohexanediol (CHD) as a chain transfer agent (CTA). CHD is proposed to undergo chain transfer (CT) reactions with the alkoxide chain intermediate, thereby end-capping the polymer chain.^45,46^ CT is assumed to be faster than the propagation of the polymer chain; hence, the overall molar mass of the polymer can be controlled by the amount of CTA added. The addition of CTA is therefore essential in the synthesis of well-defined, monodisperse polymers with target DPs. Accounting for the difference in boiling point of the epoxides, all CHO reactions were performed at 100 °C, and all reactions with PO were conducted at 60 °C. The thermal stability of all complexes at the chosen polymerization temperatures was confirmed by thermogravimetric analysis and IR spectroscopy (Figure S32 and Figure S33). All epoxide/CO_2_ ROCOP reactions were performed in a high-pressure autoclave fitted with a ReactIR probe allowing for in situ monitoring of poly(carbonate) formation (peak at 1750 cm^–1^). All epoxide/PA ROCOP reactions were conducted in a finger Schlenk tube fitted with a ReactIR probe, allowing for in situ monitoring of the disappearance of the PA peak and appearance of the poly(ester) (peaks at 1778 and 1733 cm^–1^, respectively).

The rate laws for related M(III)K(I) catalysts for both epoxide/PA and epoxide/CO_2_ ROCOP reactions are each second order, with a first-order dependence in both catalyst and epoxide concentrations.^27,32,37,47^ Using the kinetic modeling software COPASI, the conversion vs time data were fitted using the rate laws. This modeling was conducted for **1**–**3** in both CHO/CO_2_ and CHO/PA ROCOP (Figure S34 and Figure S35, Table S2).^27,32,48^ The modeled kinetic data were in excellent agreement with the experimentally observed rates (Figure S34 and Figure S35). This finding substantiates the validity of those rate laws and allows for comparisons of rate constants, which tend to be more reliable measures of rates than point kinetic TOF values. The activity for epoxide/CO_2_ ROCOP for all three catalysts was compared by using pseudo-first-order rate coefficients (*k*_obs_), which were obtained from the gradient of the semi logarithmic plot of ln([epoxide]/[epoxide]_0_) vs time (Figure S36) between 5% and 20% epoxide conversion. For epoxide/PA ROCOPs, the pseudo-zero-order rate coefficients were obtained from the slope of the linear plot of [PA] vs time between 10% and 60% PA conversion and used to compare catalyst activity (Figure S36).

In CHO/CO_2_ ROCOP, the aluminum catalyst **1** shows an excellent activity, comparable to that of cobalt catalyst **3** (Table 1, #1 and #5). Both catalysts **1** and **3** exhibited excellent polymer selectivity (97 ± 0.01% and 96 ± 0.01%), and fast rates of *k*_obs_ = 12 ± 0.006 × 10^–5^ s^–1^ and *k*_obs_ = 18 ± 0.18 × 10^–5^ s^–1^, respectively. The resulting polymers showed narrow dispersities of *Đ* < 1.10. In comparison, the iron catalyst **2** exhibited a poor activity of *k*_obs_ < 2 × 10^–5^ s^–1^ and low polymer selectivity. Despite the high activity and selectivity of Al(III)K(I) **1** in the CHO/CO_2_ ROCOP, it showed a very low polymer selectivity and activity (TOF = 6 ± 0.7 h^–1^) in PO/CO_2_ ROCOP. Indeed, its performance was the same as that for the Fe(III)K(I) **2** catalyst (Table 1, #2 and #4). Co(III)K(I) **3** showed a high activity and selectivity in PO/CO_2_ ROCOP.^16^

**Table 1:**
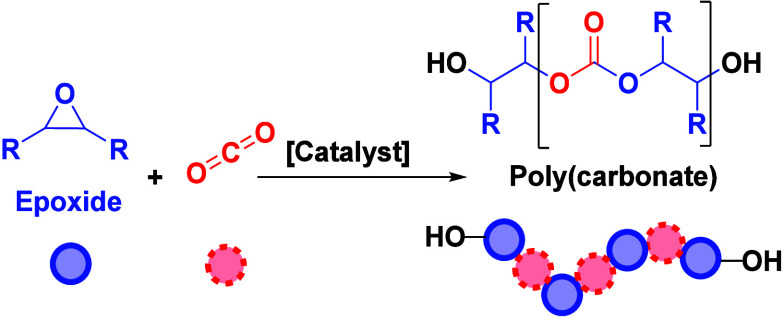
Epoxide/CO_2_ ROCOP using M(III)K(I) Catalysts^a^

#	catalyst	epoxide	time (h)	carbonate linkages (CO_2_ select.) (%)^d^	poly. select. (%)^e^	productivity TON^f^	TOF (h^–1^)^g^	rate constant *k*_obs_ x 10^–5^ (s^–1^)^h^	*M*_n_ [*Đ*] (g mol^–1^)^I^
1	Al(III)K(I) (**1**)	CHO^b^	1.5	>99	97 ± 0.01	1405 ± 180	1083 ± 68	12.0 ± 0.006	5100 [1.04]
2		PO^c^	50	98 ± 0.01	4 ± 0.01	314 ± 13	6 ± 0.7		
3	Fe(III)K(I) (**2**)	CHO^b^	26	>99	43 ± 4	471 ± 10	19 ± 2	0.05 ± 0.007	900 [1.18]
4		PO^c^,^j^	23	>99	0	44 ± 8	1.5 ± 0.4		
5	Co(III)K(I) (**3**)	CHO^b^	0.75	98 ± 0.01	96 ± 0.01	1023 ± 81	1226 ± 11	18.0 ± 0.18	3200 [1.08]
6		PO^c^,^7^	1.5	>99	99 ± 0.4		833 ± 76	8.33 ± 0.7	3800 [1.06]

a Where M(III) = Al(III), Fe(III), Co(III).

b Reaction conditions: [cat]_0_/[*trans*-1,2-cyclohexanediol]_0_/[CHO]_0_ = 1:20:4000, in 6 mL, neat CHO (9.9 M), under 20 bar CO_2_ pressure, 100 °C.

c [cat]_0_/[*trans*-1,2-cyclohexanediol]_0_/[PO]_0_ = 1:20:4000, in 6 mL neat PO (14.3 M), under 20 bar CO_2_ pressure, 60 °C.

d CO_2_ uptake was calculated by dividing the sum of integrals for polycarbonate (PPC 4.9 ppm, 1H; PCHC 4.6 ppm, 2H) and cyclic carbonate (PC 4.8 ppm, 1H; CC 4.8 ppm 2H) against the sum of integrals for polycarbonate, cyclic carbonate and polyether.

e Polymer selectivity was determined by dividing the sum of integrals for polycarbonate and polyether against the sum of integrals for polycarbonate, cyclic carbonate and polyether.

f TON was determined by dividing the moles of epoxide consumed by the moles of the catalyst (PO or CHO conversion: determined by comparison of the sum of integrals by ^1^H NMR spectroscopy of polycarbonate (PPC 4.9 ppm, 1H; PCHC 4.6 ppm, 2H), cyclic carbonate (PC 4.8 ppm, 1H; CC 4.8 ppm 2H), and polyether (3.46–3.64 ppm, 3H for PPO and 4H for PCHO)) against mesitylene (6.7 ppm) as an internal standard. Conversion data are listed in Table S4.

g Turnover frequency (TOF) was calculated by dividing the turnover number (TON) by time.

h *k*_obs_ was determined as the gradient of the plot of ln[epoxide]_*t*_/[epoxide]_0_ vs time.

I Determined by GPC in THF using narrow dispersity polystyrene standards. Representative values are shown, and representative GPC traces are shown in Figure S38.

j [cat]_0_/[*trans*-1,2-cyclohexanediol]_0_/[PO]_0_ = 1:20:2000, in 6 mL neat PO (14.3 M), under 20 bar CO_2_ pressure, 60 °C. All values are reported as an average of *n* = 2 runs, with an error of ±Δ*x* = σ/√*n* of <50% in the CHO/CO_2_ ROCOP (Table 1, #3

For CHO/PA and PO/PA ROCOP, all catalysts showed an excellent polyester selectivity of >95% (Table 2). In the CHO/PA ROCOP, Co(III)K(I) (**3**) showed the highest activity with a rate double that observed for the iron catalyst (**2**) (*k*_obs_ = 3.35 ± 0.26 × 10^–5^ M s^–1^ vs 1.50 ± 0.010 × 10^–5^ M s^–1^). The aluminum catalyst **1** was significantly slower than **2** and **3** (*k*_obs_ = 0.48 ± 0.73 × 10^–5^ M s^–1^, Table 2, #1). In PO/PA ROCOP, Co(III)K(I) (**3**) was an order of magnitude faster than either Al(III)K(I) (**1**) or Fe(III)K(I) (**2**) (Table 2, #2, #4, and #6).

**Table 2:**
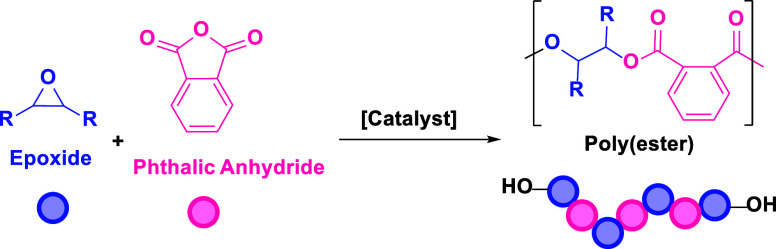
Epoxide/PA ROCOP using M(III)K(I) Catalysts^a^

#	catalyst	epoxide	time (h)	polyester selectivity (%)^d^	productivity TON^e^	TOF (h^–1^)^f^	rate constant *k*_obs_ × 10^–4^ (M s^–1^)^g^	poly(ester) *M*_n_ [*Đ*] (g mol^–1^)^h^
1	Al(III)K(I) (**1**)	CHO^b^	4	>99	330 ± 24	78 ± 5	0.48 ± 0.73	2700 [1.08]
2		PO^c^	17	>99	204 ± 11	12 ± 0.7	0.14 ± 0.008	1800 [1.11]
3	Fe(III)K(I) (**2**)	CHO^b^	2	98 ± 0.01	410 ± 7	196 ± 10	1.50 ± 0.010	3100 [1.15]
4		PO^c^	11	>99	99 ± 9	9 ± 1	0.09 ± 0.003	1400 [1.13]
5	Co(III)K(I) (**3**)	CHO^b^	1	99 ± 0.1	404 ± 2	428 ± 15	3.35 ± 0.26	7500 [1.33]
6		PO^c^	7	99 ± 0.004	401 ± 1	66 ± 7	0.80 ± 0.20	3300 [1.25]

a Where M(III) = Al(III), Fe(III), Co(III).

b Reaction conditions: [cat]_0_/[*trans*-1,2-cyclohexanediol]_0_/[PA]_0_/[CHO]_0_ = 1:20:400:4000, in 3 mL, neat CHO (9.9 M), 100 °C.

c [cat]_0_/[*trans*-1,2-cyclohexanediol]_0_/[PA]_0_;[PO]_0_ = 1:20:400:4000, in 3 mL neat PO (14.3 M), 60 °C.

d Selectivity for polyester over polyether, determined by comparison of ^1^H NMR integrals corresponding to poly(ester) PCHPE or PPE (5.22–5.06 ppm) and PCHO or PPO (3.60–3.20 ppm).

e Turnover number (TON) for polyester formation equal to the number of moles of PA consumed divided by the number of moles of the catalyst. Determined by ^1^H NMR through comparison of resonances associated with PA (8.10–7.85 ppm) and PCHPE or PPE (7.65–7.30 ppm), conversion data are listed in Table S5.

f Turnover frequency (TOF) was calculated by dividing the turnover number (TON) by time.

g *k*_obs_ was determined as the gradient of the plot of [PA] vs time.

h Determined by GPC in THF using narrow dispersity polystyrene standards. Representative values are shown, and representative GPC traces are shown in Figure S39. All values are reported as an average of *n* = 2 runs, with an error of ±Δ*x* = σ/√*n*.

The promising performance of Al(III)K(I) (**1**) in the CHO/CO_2_ ROCOP and that of Fe(III)K(I) (**2**) in the CHO/PA ROCOP prompted further investigation of the catalysis under a range of different conditions. In CHO/CO_2_ ROCOP, the aluminum catalyst (**1**) exhibited an excellent loading tolerance and retained a high TOF of 755 ± 19 h^–1^ even at a catalyst loading of 1:10 000 [cat]_0_:[CHO]_0_ (Table 3, #2). At higher temperature (120 °C), it showed an excellent activity of 1781 ± 56 h^–1^ and a good selectivity of 84%. Even at 1 bar pressure, **1** shows an activity of 71 ± 3 h^–1^ and 95% selectivity. Compared to prior catalysts, the activities and selectivity for Al(III)K(I) (**1**) stand out. At 1 bar CO_2_ pressure, **1** is the most active and selective catalyst yet reported.^27^

**Table 3:** CHO/CO_2_ ROCOP using the Al(III)K(I) Catalysts (**1**)^a^

#	*P* (bar)	*T* (°C)	time (h)	PCHC (%)^c^	productivity TON^d^	TOF (h^–1^)^e^	rate constant *k*_obs_ × 10^–5^ (s^–1^)^f^	poly(carbonate) *M*_n_ [*Đ*] (g mol^–1^)^g^
1	20	100	1.5	97 ± 0.01	1405 ± 180	1083 ± 68	12.0 ± 0.06	5100 [1.04]
2	20	100^b^	3	98 ± 0.01	2484 ± 39	755 ± 19	2.4 ± 0.05	6301 [1.18]
3	20	120	1	84 ± 0.001	1551 ± 40	1781 ± 56	16.0 ± 0.6	3900 [1.08]
4	1	70	16.5	95 ± 0	695 ± 80	43 ± 5	0.36 ± 0.04	4500 [1.08]
5	1	80	16	95 ± 0.002	1131 ± 52	71 ± 3	0.65 ± 0.08	3600 [1.13]

a Reactions: [cat]_0_/[*trans*-1,2-cyclohexanediol]_0_/[CHO]_0_ = 1:20:4000 in neat CHO (9.9 M) (6 mL for #1–3, 3 mL for #4–6).

b [cat]_0_/[*trans*-1,2-cyclohexanediol]_0_/[CHO]_0_ = 1:20:10,000, in 6 mL of neat CHO (9.9 M).

c All catalyst show >96% CO_2_ uptake. Polymer selectivity was determined by dividing the sum of integrals for polycarbonate and polyether against the sum of integrals for polycarbonate, cyclic carbonate, and polyether.

d TON was determined by dividing the moles of epoxide consumed by the moles of catalyst (CHO conversion: determined by comparison of the sum of integrals by ^1^H NMR spectroscopy of PCPC (4.92 ppm, 2H), CC (4.77 ppm, 2H), PCO (3.46–3.64 ppm, 2H) against mesitylene (6.70 ppm) as an internal standard).

e Turnover frequency (TOF) was calculated by dividing the turnover number (TON) by time.

f *k*_obs_ was determined as the gradient of the plot of ln[epoxide]_*t*_/[epoxide]_0_ vs time.

g Determined by GPC in THF using narrow dispersity polystyrene standards. Representative values are shown. All values are reported as an average of *n* = 2 runs, with an error of ±Δ*x* = σ/√*n*.

Even at higher CO_2_ pressures, Al(III)K(I) (**1**) outperforms all previously reported catalysts in CHO/CO_2_ ROCOP, except for the tethered aluminum porphyrin catalyst reported by Nozaki and co-workers (TOF = 10 000 h^–1^ (120 °C, 1:40 000 catalyst/CHO, 20 bar CO_2_)).^26^ For the CHO/PA ROCOP, Fe(III)K(I) (**2**) remained highly selective (>99%) even at low loadings and high temperatures (Table 4). An excellent activity of 1960 ± 96 h^–1^ was observed at 140 °C (Table 4, #3 and #4). Compared with previously reported iron-based catalysts, Fe(III)K(I) (**2**) outperformed most other catalysts for CHO/PA. A Fe(III)(salen)(X)/PPNX catalyst showed a TOF of only 3 h^–1^ (100 °C, 1:100 [cat]_0_/[PA]_0_).^35^ A di-iron catalyst system showed good TOF of 588 h^–1^ (100 °C, 1:100 [cat]_0_/[PA]_0_); however, four-times the catalyst loading compared to that used in this work was required.^36^

**Table 4:** CHO/PA ROCOP using Fe(III)K(I) Catalysts (**2**)^a^

#	[cat/[PA]/[CHO]^a^	*T* (°C)	TOF (h^–1^)^d^	*k*_obs_ × 10^–4^ (M s^–1^)^e^	*M*_n_ [*Đ*] (g mol^–1^)^f^
1^b^	1:400:4000	100	196 ± 10	1.50 ± 0.010	3100 [1.15]
2^c^	1:400:4000	140	1960 ± 96	15.4 ± 0.97	2500 [1.11]
3^c^	1:2000:10 000	140	1800 ± 88	2.1 ± 0.13	9600 [1.19]
4^c^	1:4000:20 000	140	1487 ± 72	1.2 ± 0.07	12000 [1.19]

a General conditions: [**2**]/[PA]/[CHO] = 1:*x*:*y*, given in table (e.g., #1: [**2**]/[PA]/[CHO] = 1:400:4000), all reaction are run in neat CHO (3 mL, 9.9 M). All catalyst show >98% selectivity for polyester over polyether, as determined by comparison of ^1^H NMR integrals corresponding to PCHPE (5.22–5.06 ppm) and PCHO (3.60–3.20 ppm).

b Values are reported as an average of *n* = 2 runs, with an error of ±Δ*x* = σ/√*n*.

c Values are reported with a standard error determined from #1, as 5% error in TOF and 6% error in *k*_obs._

d Turnover frequency (TOF) was calculated by dividing the turnover number (TON) by time, where the turnover number (TON) for polyester formation is equal to the number of moles of PA consumed divided by the number of moles of the catalyst. Moles of PA consumed was determined by ^1^H NMR spectroscopy through comparison of resonances associated with PA (8.10–7.85 ppm) and PCHPE (7.65–7.30 ppm).

e *k*_obs_ was determined as the gradient of the plot of [PA] vs time.

f Determined by GPC, in THF, using narrow dispersity polystyrene standards. Representative values are shown.

### Structure–Performance Relationships in Heterodinuclear Catalysts

Compared to the previously reported heterodinuclar catalysts (Figure 1a), both novel catalysts **1** and **2** outperform the previously reported Al(III)K(I) and Fe(III)K(I) bound in ligand framework **L3** in the CHO/CO_2_ ROCOP.^27,32,37,38,47^ The previously reported Al(III)K(I) catalyst showed a TOF of 58 h^–1^ and a selectivity of 50% at 1 bar CO_2_ pressure (1:2000 [catalyst]:[epoxide], Figure S1).^27^ In contrast, the novel Al(III)K(I) (**1**) is 95% selective and more active (TOF = 71 ± 3 h^–1^), even at a lower temperature (80 °C) and half the catalyst loading (1:4000 [catalyst]/[epoxide]). In contrast, for the CHO/PA ROCOP, the previously reported heterodinuclear Al(III)K(I) and Fe(III)K(I) catalysts utilizing **L3** and **L4** (Figure 1a) outperform catalysts **1**–**3** in CHO/PA ROCOP (TOFs > 4000 h^–1^).^27,32^ These results highlight the importance of the ligand structure in heterodinuclear catalysts: when applied with the same metal combination, **L1** is better suited to epoxide/CO_2_ ROCOP, and **L3** and **L4** are better suited to CHO/PA ROCOP.

In addition to the effect of the ancillary ligand, it is apparent that the M(III) choice also directly affects catalytic performance. Comparison of the novel Al(III)K(I) and Fe(III)K(I) catalysts bound in **L1** and **L3** shows that in both ligand frameworks Al(III)K(I) catalysts outperform the Fe(III)K(I) catalysts in epoxide/CO_2_ ROCOP, while Fe(III)K(I) catalysts show a higher activity for CHO/PA ROCOP. These observations should inform future cobalt-free catalyst design: catalysts for the epoxide/CO_2_ ROCOP should feature Al(III) centers bound in ligands similar to **L1**, while catalysts for epoxide/anhydride ROCOP should utilize Fe(III) centers bound in ligands comparable to **L3.** Unfortunately, the Co(III)K(I) complex of **L3** is synthetically inaccessible, limiting the comparison to the Al(III)K(I) and Fe(III)K(I) complexes.

While comparison of the Al(III)K(I) and Fe(III)K(I) catalysts bound in **L1** and **L3** informs some future selection principles for metals and ligands for epoxide/CO_2_ and epoxide/anhydride ROCOP catalysts, the activity of Al(III)K(I) (**1**) and Fe(III)K(I) (**2**) remains lower than that observed for the cobalt catalyst (**3**). In order to accelerate the discovery of the next generation of high-performance Earth-abundant ROCOP catalysts, it is important to try to rationalize this performance data within the series of Al(III)K(I) (**1**), Fe(III)K(I) (**2**), and Co(III)K(I) (**3**) bound in the **L1** ligand framework.

Careful examination of the characterization data for catalysts **1**–**3** illuminates some of the ground-state electronic properties of the three M(III) centers. In particular, the ligand imine bond stretching frequencies, obtained from the IR spectra, for Al(III)K(I) (**1**) and Co(III)K(I) (**3**) are the same wavenumber (1638 cm^–1^), indicating a similar imine bond strength. In contrast, the imine bond stretch for Fe(III)K(I) (**2**) is at a lower wavenumber (1624 cm^–1^). The data could be rationalized if there were some π-backbonding from the high-spin Fe(III) center. In contrast, no π-backbonding is expected to occur from the low-spin Co(III) center. These data could also indicate that the Fe(III) center is more effective at deforming the electron density around the coordinated nitrogen atom, leading to more electron density (from the nitrogen) being localized on the Fe(III) center compared to the Co(III) center.

**Figure 4:**
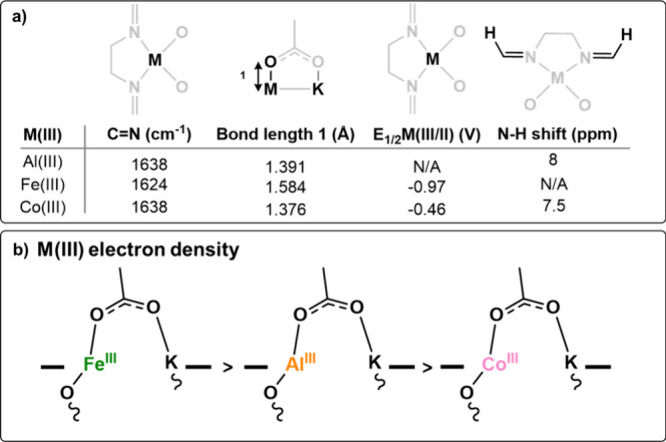
(a) Overview of the characterization data for catalysts **1**–**3** and (b) proposed increase in electron density from Fe(III) to Al(III) to Co(III).

In line with this observation, the Fe(III)–O bond length of the acetate initiator, as characterized by single crystal XRD, is significantly weaker than the equivalent bond in the Al(III)K(I) or Co(III)K(I) catalysts (Figure 4a). This bond length data is consistent with a more electron rich Fe(III) center compared to Al(III) and Co(III). Cyclic voltammetry further underlines the observation, as *E*_1/2_Fe(III/II) is lower than *E*_1/2_Co(III/II) (−0.97 vs −0.46 V). The chemical shifts of the imine and acetate signals, in the ^1^H NMR spectra, of Al(III)K(I) (**1**) and Co(III)K(I) (**3**) indicate that the Co(III) is more electropositive than Al(III) (Figure 4). Overall, the characterization data suggest that the iron center is more electron dense and hence less Lewis acidic than the aluminum and the cobalt centers (Figure 4).

**Figure 5:**
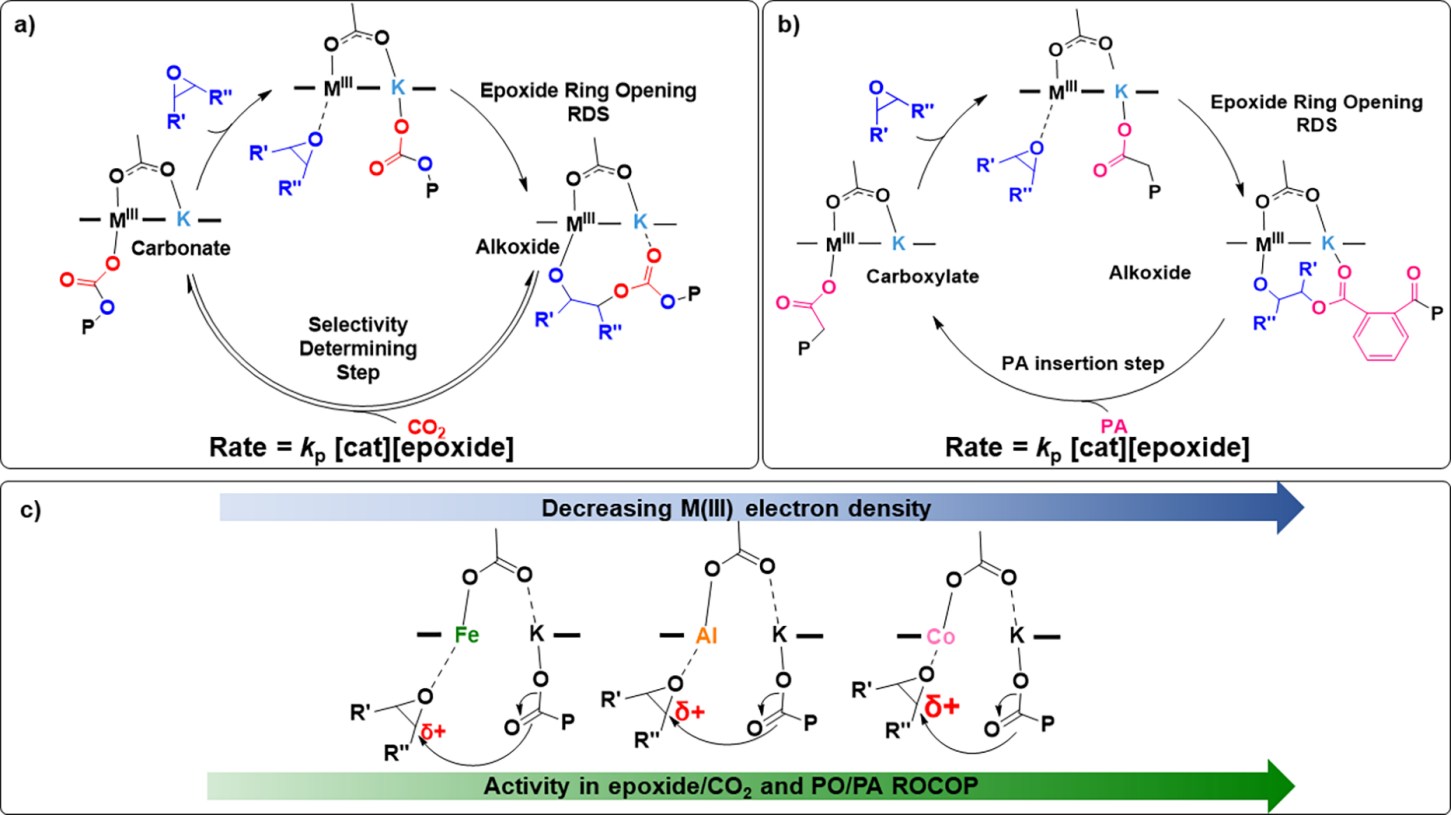
(a) Previously reported catalytic cycle for the epoxide/CO_2_ ROCOP.^16^ (b) Previously reported catalytic cycle for the epoxide/PA ROCOP.^27^ (c) Effect of decreasing M(III) Lewis acidity on the rate-determining epoxide ring-opening step.

In line with the modeled rate laws for all three catalysts, demonstrating a first-order rate dependence on [epoxide], the epoxide ring opening is assumed to be the rate-determining step (RDS) in all four epoxide/CO_2_ and epoxide/PA ROCOPs examined (Figure 5). In the RDS, the epoxide is proposed to coordinate to the M(III) center and undergo ring opening through a nucleophilic attack from the carbonate (epoxide/CO_2_ ROCOP) or carboxylate chain (epoxide/PA ROCOP) (Figure 5a and b).

Upon epoxide coordination to the Lewis acidic M(III) center, the C–O bonds in the epoxide molecule are expected to be polarized, with stronger polarization favoring the nucleophilic attack by the K(I) bound chain end (Figure 5c). It is proposed that a decreasing Lewis acidity of the M(III) center as observed from Co(III) to Al(III) and Fe(III) might lead to weaker polarization of the epoxide in the rate-determining epoxide ring opening step and hence a slower rate of epoxide ring opening.

Indeed, in CHO/CO_2_ and PO/CO_2_ ROCOP the activity and selectivity of catalysts **1**–**3** appear to inversely correlate with the proposed M(III) electron density and decreasing Lewis acidity of the M(III). Accordingly Co(III)K(I) (**3**) shows the highest activity (1226 ± 11 h^–1^ CHO/CO; 833 ± 76 h^–1^ PO/CO_2_). Next, Al(III)K(I) (**2**) shows intermediate activity (1083 ± 68 h^–1^ CHO/CO; 6 ± 0.7 h^–1^ PO/CO_2_), while Fe(III)K(I) (**3**) is least active (19 ± 2 h^–1^ CHO/CO; 1.5 ± 0.4 h^–1^ PO/CO_2_). A similar correlation to that observed for epoxide/CO_2_ ROCOP was observed for PO/PA ROCOP. Interestingly, for CHO/PA ROCOP the more electron-dense Fe(III) catalyst exhibits a higher activity (TOF = 196 ± 10 h^–1^) than Al(III) (TOF = 78 ± 5 h^–1^), but the Co(III) catalyst is most active (TOF = 428 ± 15 h^–1^). Using these monomers, previous work showed a higher rate for a related Fe(III)K(I) compared with its Al(III)K(I) analogue.^27,32^

The observed trends in epoxide/heterocumulene ROCOP activity and M(III) electron density in the heterodinuclear catalysts might suggest there is an optimum M(III) electron density for the best rate, which depends upon both the M(III) and the selected monomer combination. It may be that the correlation between M(III) electron density and catalyst activity can be used to improve the performances of other ROCOP catalysis. One future strategy to improve the performances of iron- and aluminum-based catalysts for epoxide/carbon dioxide polymerizations is to develop ligands that reduce the M(III) electron density. As such, it may be possible to modify the coordination environments of these more Earth-abundant metals so that they behave more comparably to cobalt in the catalysis.

### Estimating Catalyst Activity Per Cost

It is often claimed that using more abundant and inexpensive metals, such as iron and aluminum, is desirable. Nonetheless, less abundant metals are still used in many catalytic processes because it is essential to balance performance against cost.^16,49,50^ Having identified potential strategies to improve the performance of iron and aluminum catalysts in the future, we wanted to estimate by what factor, if any, the activity of aluminum and iron catalysts needs to improve to balance out the high cost/high performance of cobalt catalysts.

To probe this issue, we undertook a comparison guided by the approximate cost per gram of catalysts against performances in CHO/CO_2_ and CHO/PA ROCOP (Figure S40). The increasing demand for cobalt metal (batteries), geopolitical challenges associated with its mining, and the naturally lower abundance of cobalt (27 ppm) compared to aluminum (84000 ppm) and iron (52000 ppm) are reflected in the current price of the metal precursors. As a first approximation, the metal precursors (Co(II)(OAc)_2_, Fe(II)(OAc)_2_, AlEt_3_) were priced from chemical catalogues (Figure S40(i)). Second, the synthetic yield was used to quantify the amount of precursors necessary to make 1 g of each catalyst (Figure S40(ii)). Lastly, the catalogue cost of the solvents was applied (Table S6). This approximation considers only the single use of the catalyst in a batch process. However, catalysts can be efficiently removed from the polymer using various routes, such as liquid–liquid phase separation developed by Nozaki and co-workers.^51^ Therefore, in the future catalysts may be recyclable.^51−53^ The cost approximation indicates that the TOF/price (h^–1^ £^–1^ g^–1^) for the novel aluminum and iron catalysts is already higher than that for the cobalt catalyst. The results suggests that iron catalysts need to only achieve around half the rate of cobalt catalysts to become viable. This indicates that despite the lower activities of novel catalysts **1** and **2** compared to **3**, these catalysts might already be (economically) viable alternatives to the cobalt containing catalysts.

## Conclusions

Three M(III)K(I) heterodinuclear polymerization catalysts were prepared and compared using key monomer combinations so as to evaluate the different M(III) properties using cobalt, aluminum, and iron centers. Catalysts **1**–**3** were tested for four different epoxide/heterocumulene ROCOP reactions (CHO/CO_2_, PO/CO_2_, CHO/PA, and PO/PA). The novel Al(III)K(I) catalyst is the most active Al(III)-based catalyst for the CHO/CO_2_ ROCOP at 1 bar of CO_2_ pressure reported to date. A cost against performance approximation showed that the here-achieved performance of Al(III)K(I) and Fe(III)K(I) is already high enough to (economically) balance the high price of cobalt.

Systematic comparison of Al(III), Fe(III), and Co(III) bound in the same ligand framework revealed a trend in activity with M(III) electron density and informed on future design principles for high-performance, high-abundance Al(III) and Fe(III) catalysts: Al(III) catalysts should be investigated for the epoxide/CO_2_ ROCOP, while Fe(III) complexes should be prioritized for epoxide/anhydride ROCOP. Further, ligand design should focus on mediating the M(III) electron density, and different ligands should be employed for epoxide/CO_2_ and epoxide/anhydride ROCOP.

## Experimental Section

All experimental manipulations were performed using a dual-manifold nitrogen-vacuum Schlenk line or in a nitrogen-filled glovebox. All solvents and reagents were obtained from commercial sources and used as received unless stated otherwise. Acetonitrile, pentane, toluene, and THF were obtained from an SPS system, degassed by several freeze–pump–thaw cycles, further dried with 3 Å molecular sieves, and stored under N_2_. Research-grade CO_2_ (BOC, CP grade, 99.995%) was dried by passing it through two drying columns (VICI Metronics carbon dioxide purifier) in series at 50 bar pressure before use at lower pressures in the copolymerizations.

NMR analysis was performed using a Bruker AV 400 MHz spectrometer, at 298 K, unless stated. FT-IR measurements were performed using a Shimadzu IRSpirit spectrometer (installed inside the glovebox) using a single reflection ATR accessory. Gel permeation chromatography (GPC) was performed by using a Shimadzu LC-20AD instrument with two mixed bed PSS SDV linear S columns in series at 40 °C. THF was used as an eluent at a flow rate of 1 mL min^–1^. Molar mass calibration was performed using a narrow molar mass polystyrene standard. Cyclic voltammetry (CV) was carried out using a PalmSens EmStat Blue potentiostat. Cyclic voltammetry experiments were performed in a N_2_ glovebox using a three-electrode configuration, with an Au disc (2.0 mm^2^) as the working electrode, a glassy carbon electrode (2.0 mm^2^) as the counter electrode, and an Ag wire as the pseudoreference electrode. Experiments were performed using a sample solution containing 0.1 M tetrabutylammonium hexafluorophosphate as the supporting electrolyte and the analyte (ca. 5 mM) in dry, degassed acetonitrile. Experiments were performed using a 100 mV s^–1^ scan rate unless otherwise stated. Ferrocene (ca. 1 mg) was added to the sample solution as at the end of the experiment, and the measured redox potential was used as the internal standard. Elemental analysis was carried out by the London Metropolitan University (166-220 Holloway Road, London N7 8DB, United Kingdom).

### General High-Pressure Epoxide/CO_2_ Polymerization Procedure

In a nitrogen-filled glovebox, a solution of catalyst, *trans*-1,2-cyclohexene diol, and mesitylene (internal standard) in neat epoxide was prepared. The solution was then injected into a 100 mL Parr reactor fitted with a DiComp sentinel probe and attached to an ATR-IR spectrometer under a stream of dry CO_2_. The reactor was pressurized with CO_2_ to 20 bar and heated to the desired temperature. The reaction was monitored by following an increase in the polymer IR signal at 1750 cm^–1^. Upon reaction completion, the reactor vessel was cooled to room temperature and depressurized. The catalyst was quenched by the addition of a 1 M solution of benzoic acid in CHCl_3_. A sample of the crude reaction mixture was removed for NMR and GPC analysis.

### General 1 bar Epoxide/CO_2_ Polymerization Procedure

In a nitrogen-filled glovebox, a solution of catalyst, *trans*-1,2-cyclohexene diol and mesitylene (internal standard) in neat epoxide was prepared in a finger Schlenk tube. The reaction was then carefully degassed in vacuo (3×), and the Schlenk tube was backfilled with dry CO_2_ each time. Under a flow of dry CO_2_ (1 bar pressure), the Schlenk tube was then fitted with a DiComp sentinel probe, which was attached to an ATR-IR spectrometer. The reaction was monitored by following increase in polymer IR signal at 1750 cm^–1^. Upon completion of the reaction, the reactor vessel was cooled to room temperature, and the catalyst was quenched by the addition of a 1 M solution of benzoic acid, in CHCl_3_. A sample of the crude reaction mixture was removed for NMR and GPC analysis.

### General Epoxide/PA Polymerization Procedure

In a nitrogen-filled glovebox, a solution of catalyst, *trans*-1,2-cyclohexene diol and mesitylene (internal standard) in neat epoxide was prepared in a finger Schlenktube. The Schlenk tube was then moved to a Schlenk line and, under a continuous flow of N_2_, fitted with an ATR-IR spectrometer. The Schlenk tube, fitted with the ATR-IR spectrometer, was then transferred into a preheated oil bath. The reaction was monitored by following increase in polymer IR signal at 1779 cm^–1^ and the disappearance of the phthalic anhydride peak at 1733 cm^–1^. Upon reaction completion, the Schlenk tube was removed from the oil bath and cooled to room temperature. The catalyst was quenched by the addition of a 1 M solution of benzoic acid in CHCl_3_. A sample of the crude reaction mixture was removed for NMR and GPC analysis.

### Synthesis of **H**_**2**_**L**_**1**_

The ligand **H**_**2**_**L**_**1**_ was prepared according to a previously reported literature procedure.^16^ Ethylenediamine (166 μL, 2.6 mmol) was added to a stirred solution of 2-hydroxy-3-(2-methoxyethoxy)benzaldehyde (1 g, 5.2 mmol) in a minimum amount of MeOH (200 mL). The solution was left to stir for 2 h until precipitation of a yellow solid was observed. The precipitate was isolated by vacuum filtration, and the resulting bright yellow solid was dried under high vacuum for 16 h (900 mg, 80%). The obtained NMR spectra are in good agreement with the literature, supporting the successful formation of **H**_**2**_**L**_**1**_. ^1^H NMR (500 MHz, CDCl_3_) δ 13.56 (s, 2H, O–H, (i), 8.32 (s, 2H, N=C—H, b), 6.96 (dd, *J* = 7.9, 1.5 Hz, 2H, Ar–H_m,_ c/e), 6.87 (dd, *J* = 7.8, 1.5 Hz, 2H, Ar–H_m,_ c/e), 6.76 (t, *J* = 7.9 Hz, 2H, Ar–H_p,_ d), 4.19 (dd, *J* = 5.8, 4.2 Hz, 4H, CH_3_–O–CH_2_, g), 3.94 (s, 4H, CH_2_–O–CH_2_, a), 3.79 (dd, *J* = 5.7, 4.3 Hz, 4H, N–CH_2_, f), 3.45 (s, 6H, CH_3_, h). ^13^C NMR (126 MHz, CDCl_3_) δ 166.7 (N=C, b), 152.1 (Ar–C–OH, h), 147.4 (Ar–C_ortho_, c), 124.1 (Ar–C_meta_, d/f), 118.8 (Ar–C_ortho_–O–CH_2_, g) 118.0 (Ar–C_para_–H, e), 117.3 (Ar–C_meta_, d/f), 71.1 (Ar–O–CH_2_, (i), 68.8 (CH_2_–CH_2_–O, j), 59.6 (N–CH_2_, a), 59.2 (CH_3_, k)

### **L**_**1**_**Al(III)Et** Synthesis

The **L**_**1**_**Al(III)Et** was synthesized by metalation of **H**_**2**_**L**_**1**_. **H**_**2**_**L**_**1**_ (500 mg, 1.2 mmol) was dissolved in THF or 2-MeTHF (5 mL), and AlEt_3_ (164.5 μL, 1.2 mmol) was added to THF or 2-MeTHF (1 mL). Both solutions were cooled to −30 °C in a freezer. Both solutions were combined, left to warm to room temperature, and stirred for 2 h. **L**_**1**_**Al(III)Et** was isolated as a bright yellow, free-flowing solid by removal of the solvent in vacuo (560 mg, >99%). ^1^H NMR (500 MHz, CDCl_3_) δ 8.12 (s, 2H, N=C—H, b), 7.01 (dd, *J* = 7.7, 1.7 Hz, 2H, Ar–H_m,_ c/e), 6.69 (dd, *J* = 7.8, 1.7 Hz, 2H, Ar–H_m,_ c/e), 6.49 (t, *J* = 7.7 Hz, 2H, Ar–H_p,_ d), 4.44–4.35 (m, 2H, N–CH_2_, a/a′), 4.32–4.25 (m, 2H, N–CH_2_, a/a′), 3.88 (qd, *J* = 8.4, 3.5 Hz, 2H, CH_2_-O–CH_2_, f or CH_3_–O–CH_2_, g), 3.76–3.64 (m, 6H CH_2_–O–CH_2_, f or CH_3_–O–CH_2_, g), 3.39 (s, 6H, CH_3_, h)), 0.64 (t, *J* = 8.1 Hz, 3H, Al–CH_2_, i), −0.48 (q, *J* = 8.1 Hz, 2H, Al–CH_2_–CH_3_, j). ^13^C NMR (126 MHz, CDCl_3_) δ 168.35 (N=C), 158.26 (Ar–C–OH), 150.21 (Ar–C_ortho_–O–CH_2)_, 126.69 (Ar–C_meta_), 123.58 (Ar–C_meta)_, 119.77 (Ar–C_ortho_–O–CH_2_), 115.49 (Ar–C_para_), 71.58 (CH_2_–CH_2_–O or Ar–O–CH_2_), 69.82 (N–CH_2_), 58.94 (CH_2_–CH_2_–O or Ar–O–CH_2_), 54.53 (CH_3_), 9.91 (Al–CH_2_–CH_3_). *v*_max_ (cm^–1^) 2907 (C(sp^2^)–H, 1617 (C=N).

### **L**_**1**_**Al(III)OAc** Synthesis

**L**_**1**_**Al(III)OAc** was synthesized by ligand exchange of **L**_**1**_**Al(III)Et** using AcOH. **L**_**1**_**Al(III)Et** (560 mg, 1.2 mmol) was redissolved in THF or 2-MeTHF (10 mL), and acetic acid (67.5 μL, 1.2 mmol) was added to the solution. The resultant yellow solution was stirred for 16 h. After 16 h, **L**_**1**_**Al(III)OAc** formed as a bright yellow precipitate, which was isolated by cannular filtration. **L**_**1**_**Al(III)OAc** was dried in vacuo and was isolated in a good yield (540 mg, 90%). Found: C, 57.93; H, 5.46; N, 5.30. Calcd.: C, 57.60; H, 5.84; N, 5.60. *v*_max_ (cm^–1^) 2910 (C(sp^2^)–H, 1634 (C=N), 1566 (acetate)^1^H NMR (500 MHz, CDCl_3_) δ 8.18 (s, 2H, N=C–H, b), 6.95 (d, *J* = 7.8 Hz, 2H, Ar–H_m,_ c/e), 6.66 (d, *J* = 7.7 Hz, 2H, Ar–H_m,_ c/e), 6.51 (t, *J* = 7.8 Hz, 2H, Ar–H_p,_ d), 4.29 (s, 4H, CH_2_-O–CH_2_, f or CH_3_–O–CH_2_, g), 4.16 (s, 4H, N–CH_2_, a), 3.73 (s, 4H, CH_2_-O–CH_2_, f or CH_3_–O–CH_2_, g), 3.45 (s, 6H, CH_3_, h). ^13^C NMR (126 MHz, CDCl_3_) δ 168.24(N=C), 156.09 (Ar–C–OH or Ar–C_ortho_), 150.43 (Ar–C–OH or Ar–C_ortho_), 127.02 (Ar–C_meta_), 122.67 (Ar–C_meta_), 121.87 (Ar–C_ortho_–O–CH_2_), 117.13 (Ar–C_para_), 71.87 (CH_2_–CH_2_–O or CH_2_–CH_2_–O), 70.06 (CH_2_–CH_2_–O or CH_2_–CH_2_–O), 59.36 (CH_3_), 54.78 (N–CH_2_), 23.52 (H_3_C—C=O (OAc).

### Al(III)K(I) (**1**) Synthesis

Complex **1** was prepared by the addition of KOAc (58.9 mg, 0.6 mmol) to a stirred solution of **L**_**1**_**Al(III)OAc** (300 mg, 0.6 mmol) in DCM (10 mL). The solution was stirred for 16 h, and complex **1** was isolated by the removal of the solvent in vacuo. Complex **1** was subsequently purified by azeotropic washes (3× toluene (10 mL × 3) and then 3× pentane (10 mL × 3)). Subsequently, complex **1** was dried in vacuo for 16 h and isolated in a good yield (200 mg, 55%). Alternatively, complex **1** was prepared by adding KOAc (58.9 mg, 0.6 mmol) to a stirred solution of **L**_**1**_**Al(III)OAc** (300 mg, 0.6 mmol) in 2-MeTHF (20 mL), and the solution was heated to 70 °C for 2 h and then stirred at 45 °C for 16 h. Upon cooling, complex **1** precipitated from the solution, was isolated by vacuum filtration, and dried in vacuo for 16 h. Complex **1** was isolated in good yield (291 mg, 80%). Found: C, 52.58; H, 5.00; N, 4.97. The obtained EA data correspond to Al(III)K(I)(OAc)_2_, which is calculated as C, 52.17; H, 5.39; N, 4.68. *v*_max_ (cm^–1^) 2909 (C(sp^2^)–H, 1638 (C=N), 1607 (acetate) ^1^H NMR (500 MHz, CDCl_3_) δ 7.98 (s, 2H, N=C—H, b), 6.85–6.79 (m, 4H, Ar–H_m,_ c + e), 6.47 (t, *J* = 7.8 Hz, 2H, Ar–H_p,_ d), 4.04 (t, *J* = 4.4 Hz, 4H, N–CH_2_, a), 3.74–3.68 (m, 4H, CH_3_–O–CH_2_, g or Ar–O–CH_2_), 3.37 (s, 6H, CH_3_, h), 1.57 (s, 6H, OAc, h). ^13^C NMR (126 MHz, CDCl_3_) δ 174.49 (C=O (OAc, m), 164.00 (N=C, j), 155.82 (Ar–C_ortho_, k), 149.97 (Ar–C–O^–^), 126.29 (Ar–C_meta_, c or e), 119.61 (Ar–C_ortho_–O–CH_2_), 116.14 (Ar–C_meta_, c or e), 114.00 (Ar–C_para_, d), 70.74 (CH_2_–CH_2_–O, g or CH_2_–CH_2_–O, f), 66.46 (CH_2_–CH_2_–O, g or CH_2_–CH_2_–O, f), 58.01 (CH_3_), 54.34 (N–CH_2_), 24.30 CH_3_ (OAc), l).

### Fe(III)K(I) (**2**) Synthesis

Complex **2** was synthesized following a modified literature procedure.^16,32^ To a stirred solution of **H**_**2**_**L**_**1**_ (400 mg, 0.96 mmol) in THF or 2-MeTHF (7 mL) was added Fe(II)(OAc)_2_ (167 mg, 0.96 mmol). The resulting dark red solution was stirred in an inert atmosphere for 2 h and then opened to air and stirred in air for 16 h. KOAc (94 mg, 0.96 mmol) was subsequently added to the reaction mixture, and the solution was stirred for another 16 h. The solvent was removed in vacuo to afford a dark purple crystalline solid. Six azeotropic washes were performed (3× toluene (10 mL × 3) and 3× pentane (10 mL × 3)). Complex **2** was dried in vacuo for 16 h, and the dark purple solid product was isolated in good yield (460 mg, 73% from THF and 474 mg from 2-MeTHF). *v*_max_ (cm^–1^) 2893 (C(sp^2^)–H), 1624 (C=N), and 1595 (acetate). Found: C, 50.74; H, 5.16; N, 4.32. This corresponds to a Fe(III)K(I)(OAc)_2_·THF_0.25_ adduct, which is calculated as C, 50.43; H, 5.36; N, 4.32.

### Co(III)K(I) (**3**) Synthesis

Complex **3** was synthesized according to a previously reported procedure.^16^**H**_**2**_**L**_**1**_ (300 mg, 0.72 mmol), K(OAc) (71 mg, 0.72 mmol), and Co(OAc)_2_ (128 mg, 0.72 mmol) were stirred in dry acetonitrile (100 mL) for 16 h under an inert N_2_ atmosphere. The solution was then opened to air, and acetic acid (82.4 μL, 1.44 mmol) was added. The solution was stirred for 48 h in air. The solvent was removed in vacuo, and six azeotropic washes (toluene (3 × 10 mL), pentane (3 × 10 mL)) were performed. The complex was reprecipitated from DCM/pentane (75 mL). The resulting brown fluffy material was dried under high vacuum for 24 h to afford **3** in a good yield (225 mg, 75%). The obtained NMR spectra are in good agreement with the literature, confirming the successful formation of **3**. ^1^H NMR (500 MHz, CDCl_3_) δ 7.64 (s, 2H, N=C—H, b), 6.80 (d, *J* = 7.8 Hz, 2H, Ar–H_m,_ c/e), 6.71 (d, *J* = 7.5 Hz, 2H, Ar–H_m,_ c/e), 6.34 (t, *J* = 7.7 Hz, 1H, Ar–H_p,_ d), 4.26 (s, 4H, N–CH_2_, a), 4.14–4.09 (m, 4H, CH_2_-O–CH_2_, f), 3.80–3.75 (m, 4H, CH_3_–O–CH_2_, g), 3.41 (s, 6H, CH_3_, h), 1.38 (s, 6H, O–Ac, i). ^13^C NMR (126 MHz, CDCl_3_) δ 179.74 (C=O (OAc)), 165.02 (N=C), 157.46 (Ar–C–OH), 152.23 (Ar–C_ortho_–O–CH_2_), 126.66 (Ar–C_meta_), 119.16 (Ar–C_ortho_), 114.62 (Ar–C_meta_), 112.61 (Ar–C_para_), 70.72 (CH_2_–CH_2_–O), 66.75 (Ar–O–CH_2_), 59.10 (N–CH_2_), 58.28 (CH_3_), 24.58 (H_3_C—C=O (OAc)). *v*_max_ (cm^–1^) 2914 (C(sp^2^)–H). Found: C, 49.19; H, 4.85; N, 4.33. Calcd. for C_26_H_32_N_2_O_10_CoK: C, 49.52%; H, 5.12%; N, 4.44%.

## Supporting Information Available

Characterization data of catalysts (1D and 2D NMR spectra, infrared spectra, cyclic voltammetry spectra, and X-ray crystallography) and COPASI modeling details (PDF)

## Abbreviations

ROCOP: ring-opening copolymerization
PO: propene oxide
CHO: cyclohexane oxide
PA: phthalic anhydride
RDS: rate-determining step
