# Supplementary material for: Understanding the Effect of M(III) Choice in Heterodinuclear Polymerization Catalysts
Source: Inorg Chem. 2024 Nov 19;63(49):23438–49. doi: 10.1021/acs.inorgchem.4c04430 (PMC11632767; doi:10.1021/acs.inorgchem.4c04430)
Supplement: Supplementary file 1 — ic4c04430_si_001.pdf [file ic4c04430_si_001.pdf]

Understanding the effect of M(III) choice in heterodinuclear polymerization catalysts

Katharina H.S. Eisenhardt, [a] Francesca Fiorentini [a] and Charlotte K. Williams [*a]

[a]Department Chemistry, University of Oxford, Chemistry Research Laboratory, 12 Mansfield Road, Oxford, OX1 3TA UK

Table of Contents

Additional Information ... 4

Fig. S1 Previously reported Al(III)K(I) catalyst for the CHO/CO2 ROCOP ... 4

Fig. S2 Previously reported Fe(III)K(I) catalyst for the CHO/PA ROCOP ... 5

Fig. S3 ^{1}H NMR spectrum of H_2L_1 in $CDCl_3$. ... 6

Fig. S4 ^{13}C NMR spectrum of H_2L_1 in $CDCl_3$. ... 7

Fig. S5 ^{1}H NMR spectrum of L_1AlEt in $CDCl_3$. ... 8

Fig. S6 ^{13}C NMR spectrum of L_1AlEt in $CDCl_3$. ... 9

Fig. S7COSY NMR spectrum of L_1AlEt in $CDCl_3$. ... 10

Fig. S8 HSQC NMR spectrum of L_1AlEt in $CDCl_3$. ... 11

Fig. S9 HMBC NMR spectrum of L_1AlEt in $CDCl_3$. ... 12

Fig. S10 IR spectra of a) L_1AlEt, b) L_1AlOAc, c) Al(III)K(I) (1). ... 13

Fig. S11 ^{1}H NMR spectrum of L_1AlOAc in $CDCl_3$. ... 14

Fig. S12 ^{13}C NMR spectrum of L_1AlOAc in $CDCl_3$. ... 15

Fig. S13 COSY NMR spectrum of L_1AlOAc in $CDCl_3$. ... 16

Fig. S14 HSQC NMR spectrum of L_1AlOAc in $CDCl_3$. ... 17

Fig. S15 HMBC NMR spectrum of L_1AlOAc in $CDCl_3$. ... 18

Fig. S16 ^{1}H NMR spectrum of 1 in $CDCl_3$. ... 19

Fig. S17 ^{13}C NMR spectrum of 1 in $CDCl_3$. ... 20

Fig. S18 COSY NMR spectrum of 1 in $CDCl_3$. ... 21

Fig. S19 HSQC NMR spectrum of 1 in $CDCl_3$. ... 22

Fig. S20 HMBC NMR spectrum of 1 in $CDCl_3$. ... 23

Fig. S21 DOSY spectrum of 1 in $CDCl_3$ with Tetrakis(trimethylsilyl)silane. ... 24

Fig. S22 IR spectra of complexes a) Al(III)K(I) (1), b) Fe(III)K(I) (2) and c) Co(III)K(I) (3). ... 25

Fig. S23 Plot of χ mol^{-1} *vs*. temperature for complexes 2 obtained via SQUID magnetometry. ... 26

Table S1. Magnetometry data for complexes 2. ... 26

Fig. S24 Cyclic Voltammogram of 2 in MeCN, ... 27

Fig. S25 a) Overlay of cyclic voltammograms of (2) obtained at different scan rates, b) Linear relationship between the peak current and square root of the scan rate. ... 27

Fig. S26 ^{1}H NMR spectrum of 3 in $CDCl_3$. ... 28

Fig S27 ^{13}C NMR spectrum of 3 in $CDCl_3$. ... 29

Fig. S28 Cyclic Voltammogram of 3 in MeCN, ... 30

'Greener' Synthesis Design ... 31

Fig. S29 Overlay of IR spectra of Fe(III)K(I) (2) synthesised in THF and 2-MeTHF. ... 31

Fig. S30 Comparison CV spectra (1.6 mM Fe(III)K(I) in MeCN, v = 0.1 V) for Fe(III)K(I) (2) synthesised a) in THF, ... 31

Fig. S31 Comparison of ^{1}H NMR spectra of Al(III)K(I) in $CDCl_3$ synthesised in 2-MeTHF (top spectrum) and synthesised in THF and DCM (bottom spectrum). 32

Thermal stability of catalyst 1-3 33

Fig. S32 Heat stability of 1-3 33

Fig. S33 Heat stability of 1-3 34

Table S2 Key IR stretches before and after heating experiments 34

COPASI models 35

Fig. S34 Concentration vs time data modelled using the previously reported rate law for CHO/CO2 ROCOP in COPASI 35

Fig. S35 Concentration vs time data modelled using the previously reported rate law for CHO/PA ROCOP in COPASI 35

Table S3 Parameters and rate equations used for COPASI models. 36

Polymerisation data 37

Fig. S36 a) Exemplary, linear semilogarithmic plot of ln([CHO]/[CHO]$_0$) vs time between 5-20 % epoxide conversion, used to determine k_{obs} for CHO/CO_2, b) a) Exemplary, linear plot of [PA] vs time between 10-60% PA conversion, used to determine k_{obs} for CHO/PA. 37

Fig. S37 Conversion over time data for the polymerisation of PO/CO_2 ROCOP using Co(III)K(I) (3), ... 37

Table S4 Conversion data for the epoxide/CO2 ROCOP reactions reported in Table 1 38

Table S5 Conversion data for the epoxide/PA ROCOP reactions reported in Table 2 38

Fig. S38 Representative GPC traces for Table 1, #1, #3, #4 39

Fig. S39 Representative GPC traces for Table 2, #1-#6. 39

Approximating catalyst activity per cost 40

Table S6 Activities and prices considered in the cost estimate (Figure 4). 40

Figure S40 Plots showing the TOF / price (h^{-1} £$^{-1}$ g^{-1}) 40

Crystallographic Details 41

Table S7 Selected bond lengths for Al(III)Et , Al(III)K(I) (1) and Fe(III)K(I) (2). 41

Table S8 Selected bond angles for Al(III)Et , Al(III)K(I) (1) and Fe(III)K(I) (2). 42

Table S9 Summary of crystallographic refinement data for complexes Al(III)-Et, Al(III)K(I) (1) and Fe(III)K(I) (2). 43

Additional Information

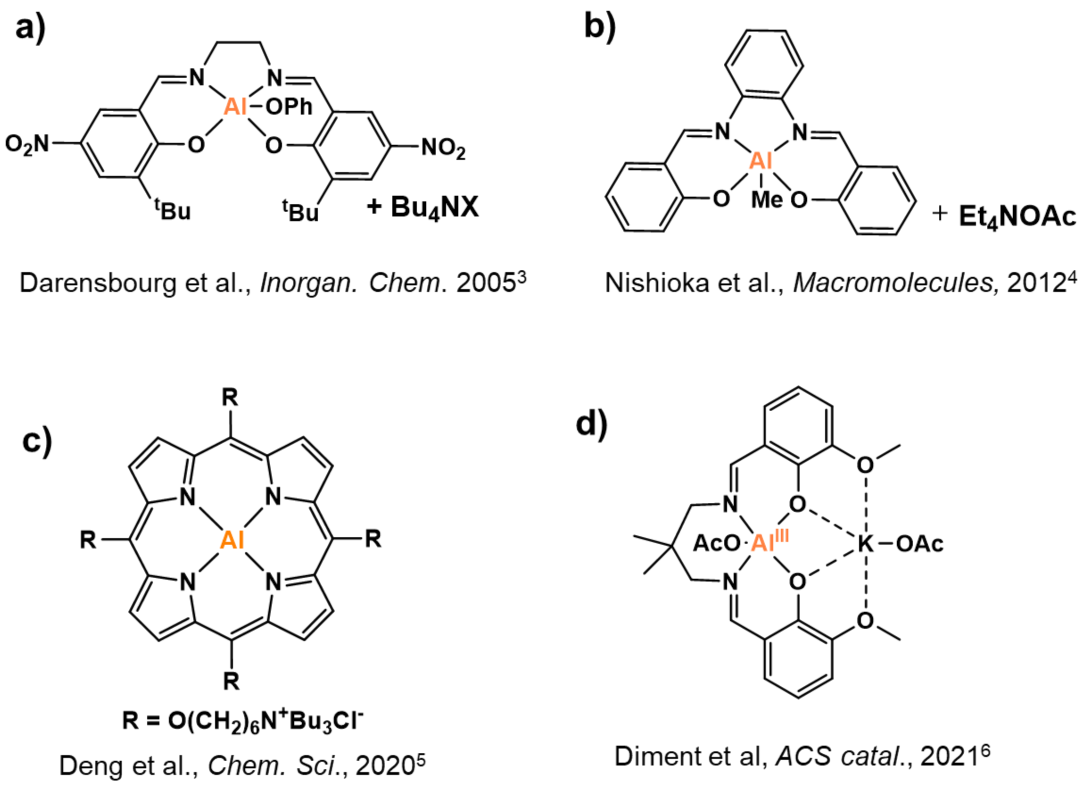

Fig. S1 Previously reported Al(III)K(I) catalyst for the CHO/CO2 ROCOP a) Monometallic aluminium salen catalyst reported by Darensbourg and co-workers.[1] b) Monometallic aluminium salen catalyst reported by Nishioka et al.[2], c) Monometallic aluminium porphyrin reported by Deng et al.[3] d) Heterodinuclear aluminium potassium catalyst reported by Diment et al.[4]

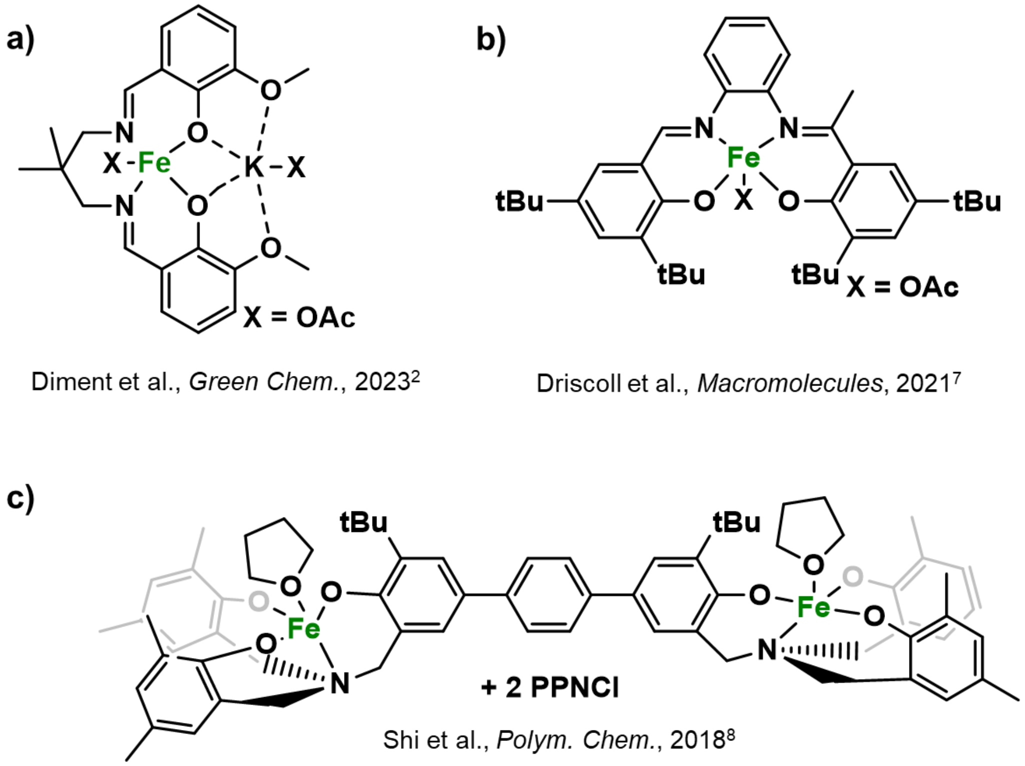

Fig. S2 Previously reported Fe(III)K(I) catalyst for the CHO/PA ROCOP a) Heterodinuclear iron potassium catalyst reported by Diment et al.[2] b) Monometallic iron salen catalyst reported by Driscoll et al.[7] c) Homodinuclear iron catalyst reported by Shi et al.[8]

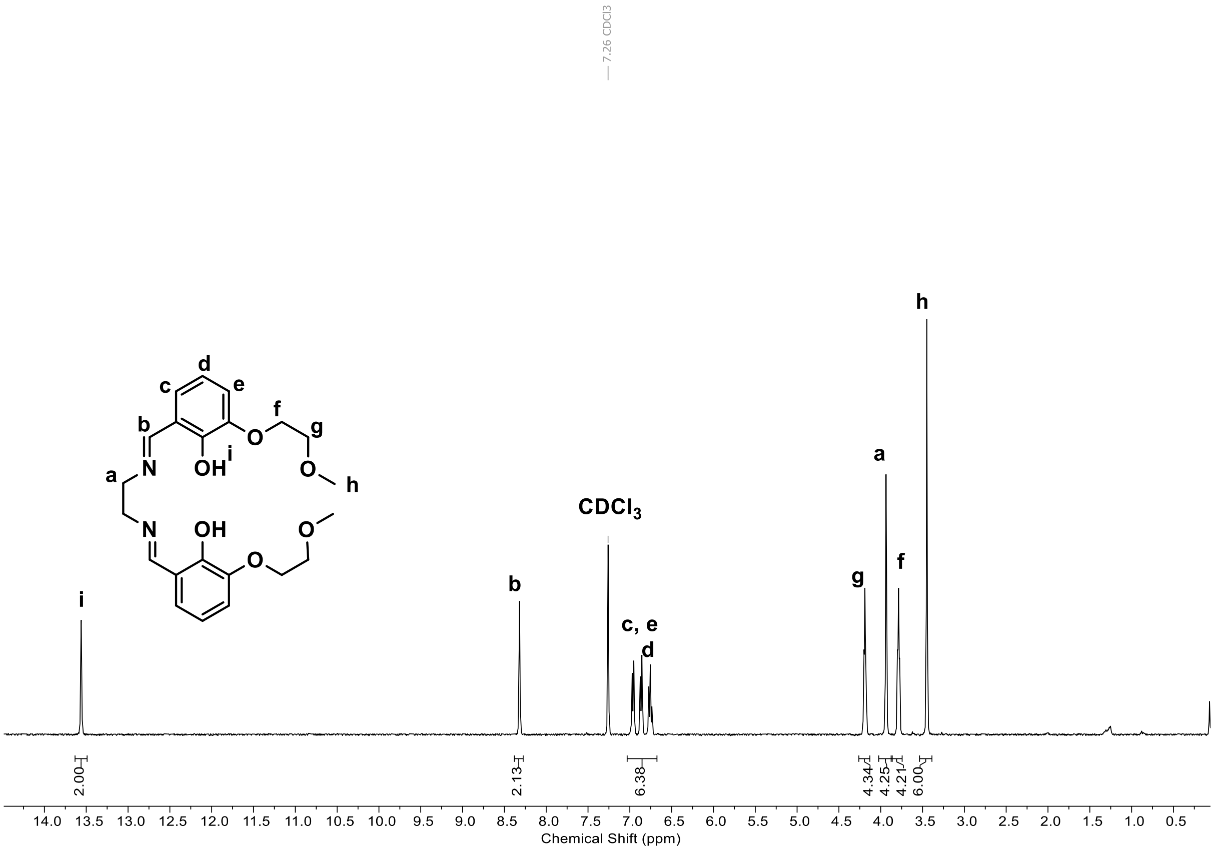

Fig. S3 ^{1}H NMR spectrum of H_2L_1 in $CDCl_3$.

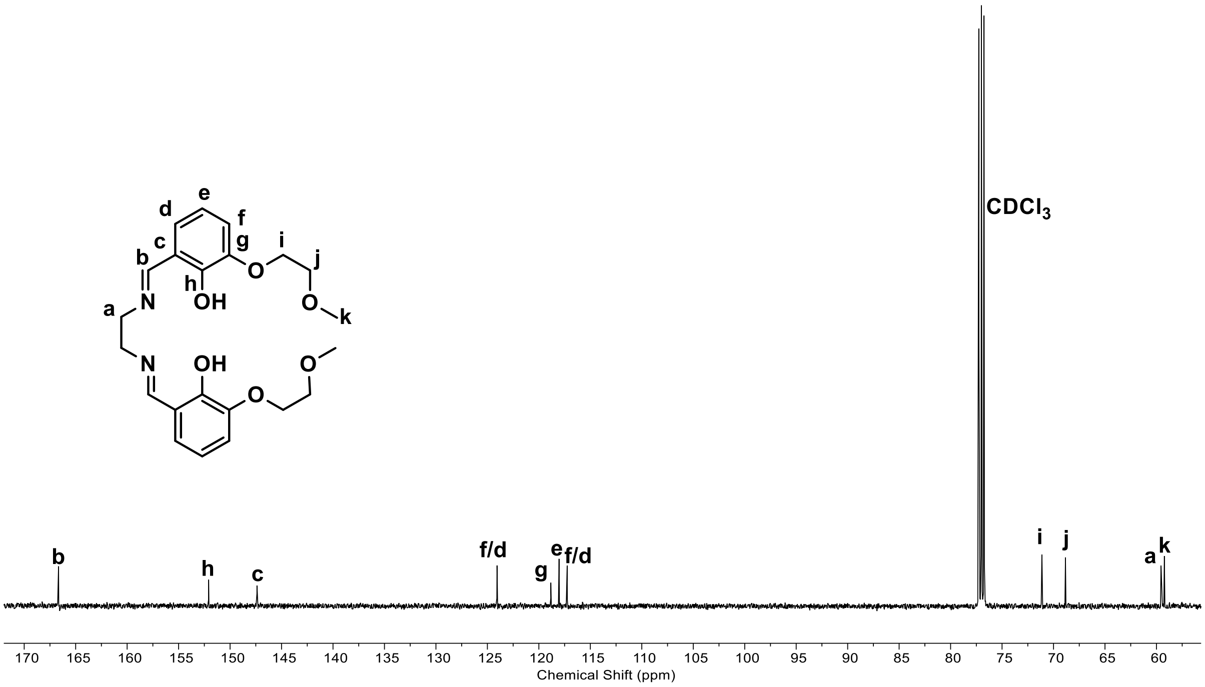

Fig. S4 ^{13}C NMR spectrum of H_2L_1 in $CDCl_3$.

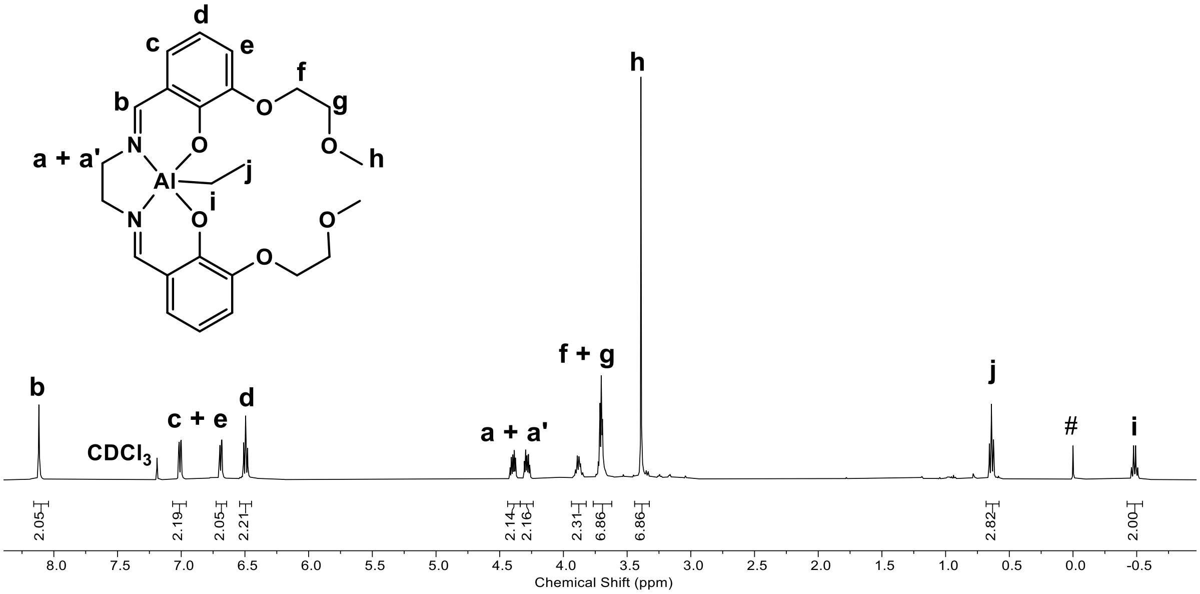

Fig. S5 ^{1}H NMR spectrum of L_1AlEt in $CDCl_3$. (# Denotes vacuum grease)

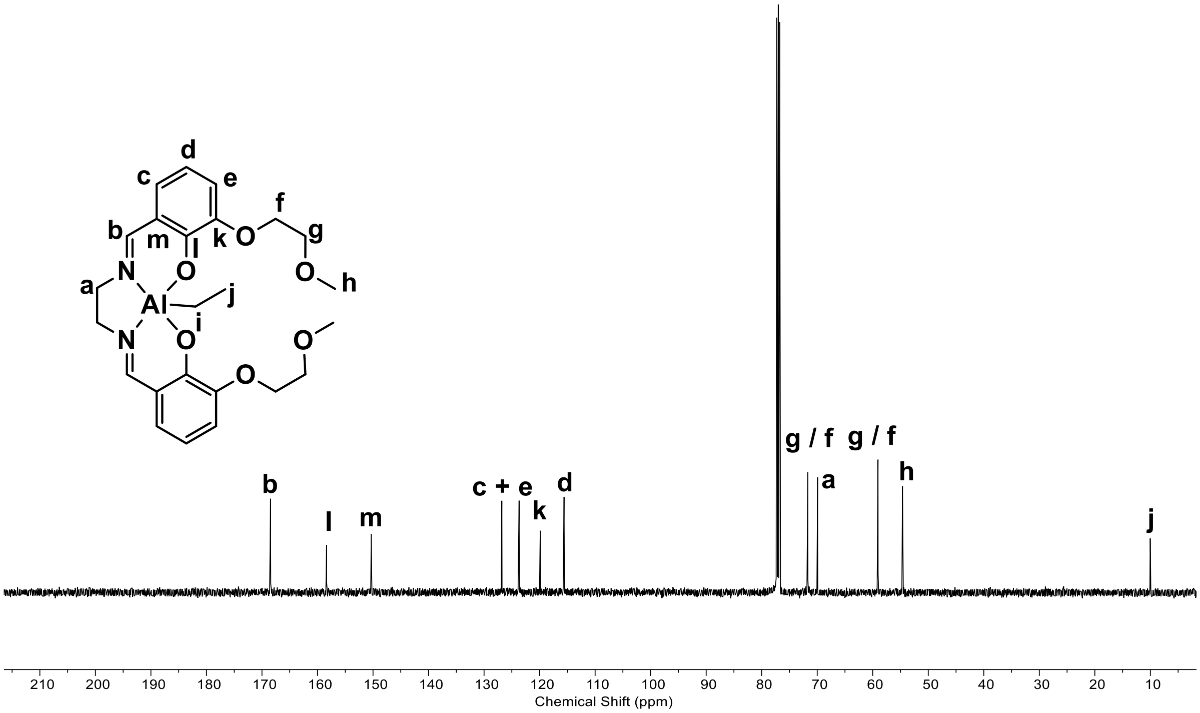

Fig. S6 ^{13}C NMR spectrum of L_1AlEt in $CDCl_3$.

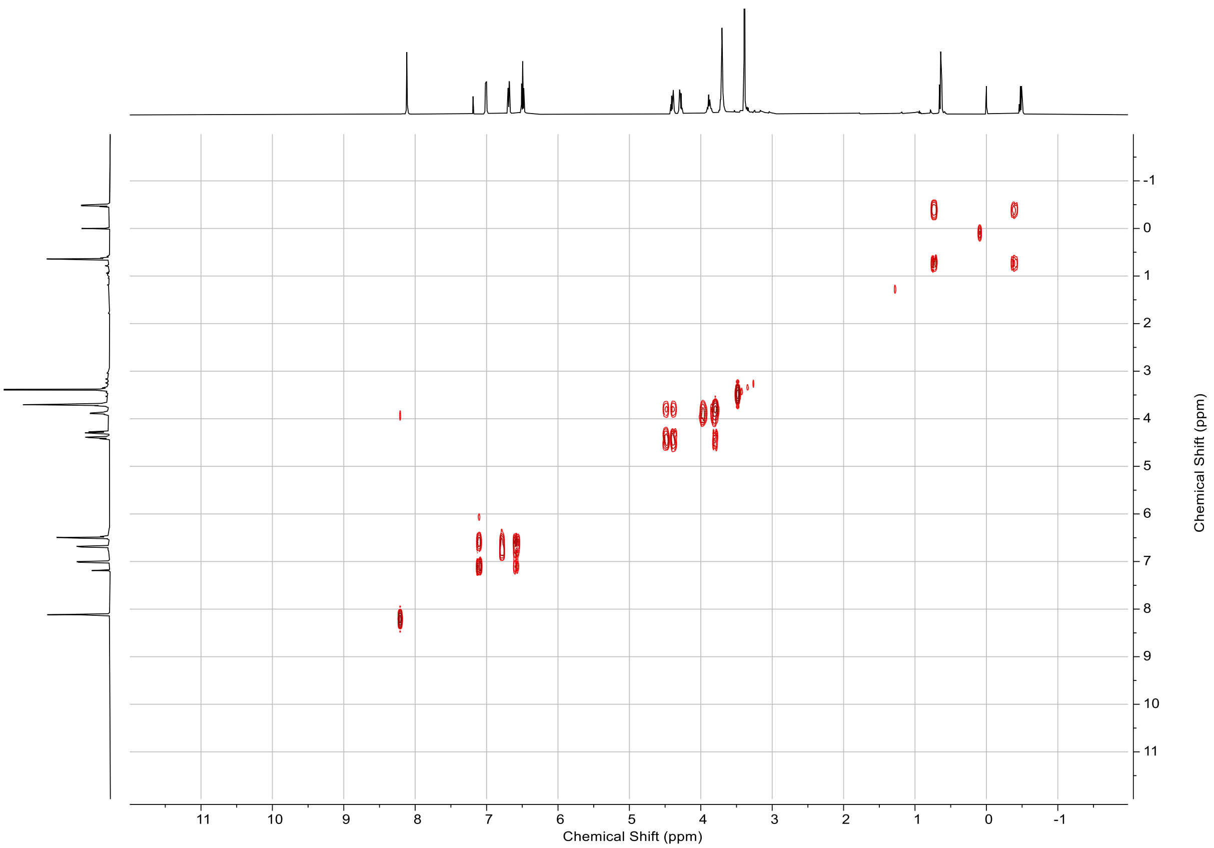

Fig. S7COSY NMR spectrum of L_1AlEt in $CDCl_3$.

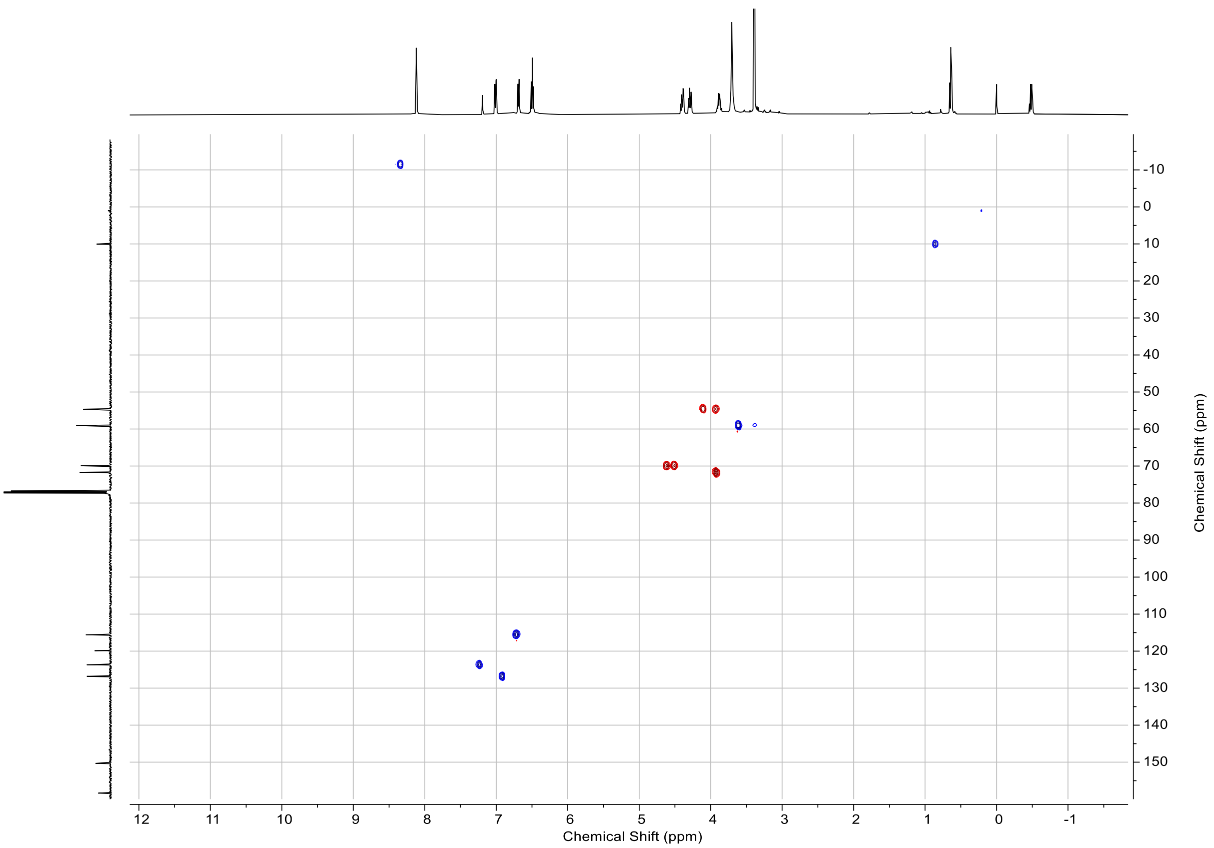

Fig. S8 HSQC NMR spectrum of L_1AlEt in $CDCl_3$.

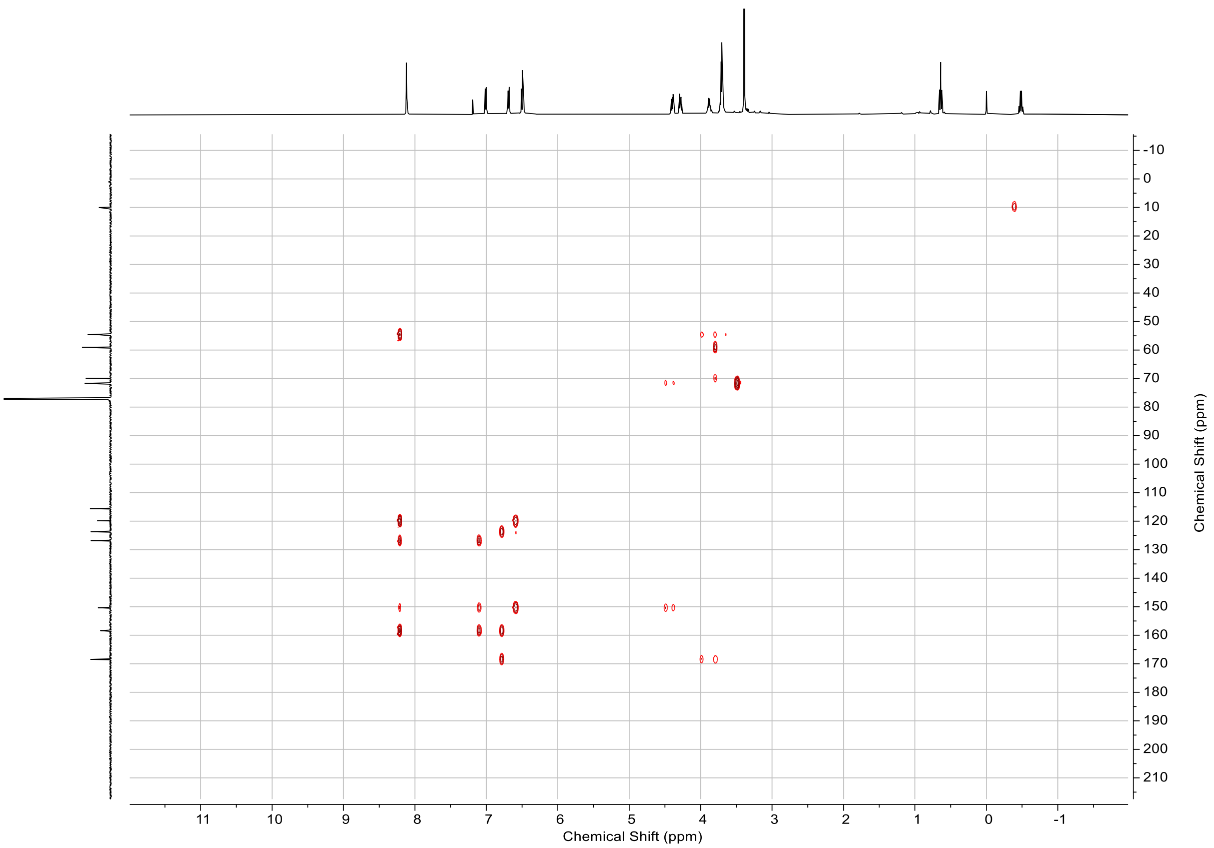

Fig. S9 HMBC NMR spectrum of L_1AlEt in $CDCl_3$.

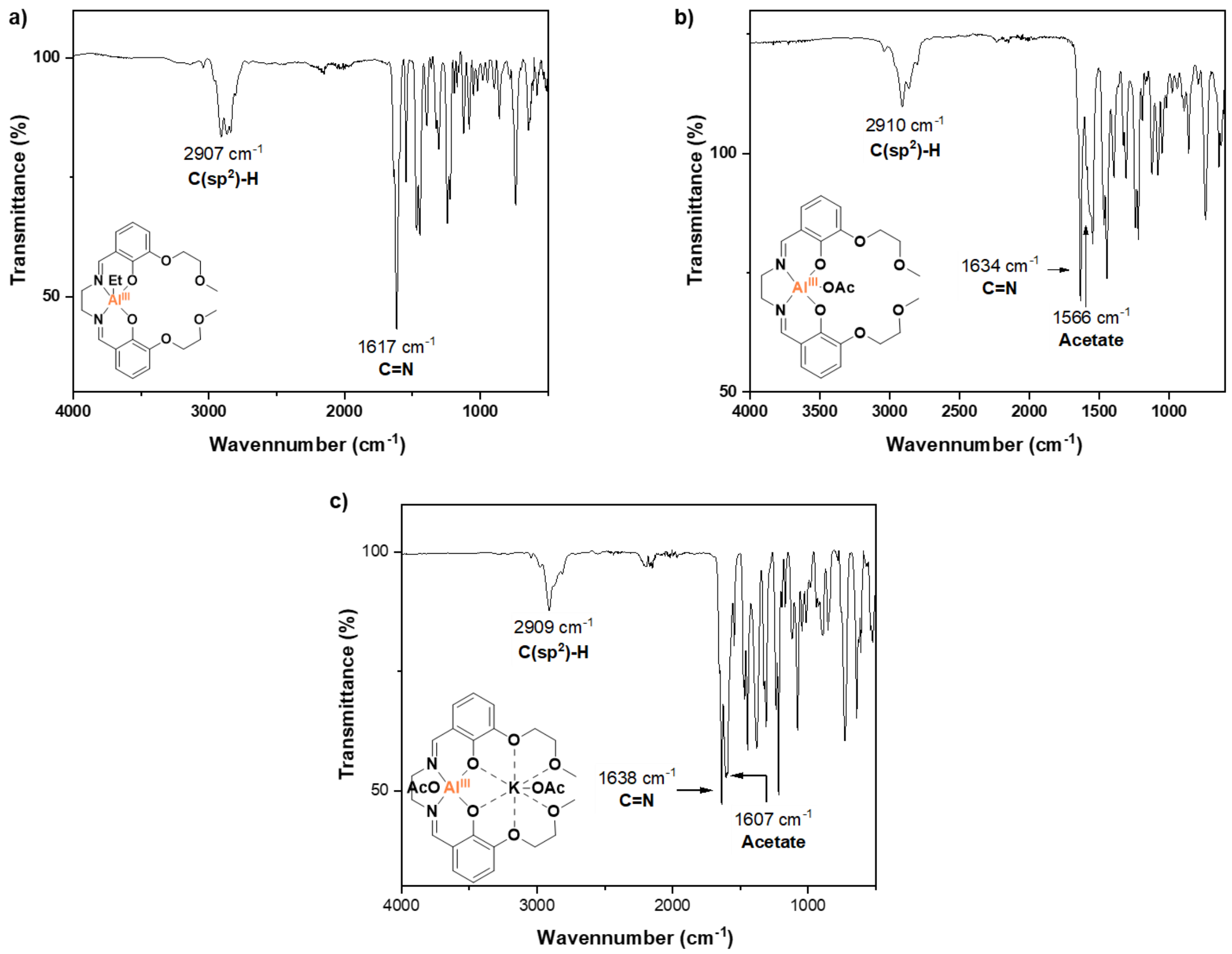

Fig. S10 IR spectra of a) L_1AlEt, b) L_1AlOAc, c) Al(III)K(I) (1).

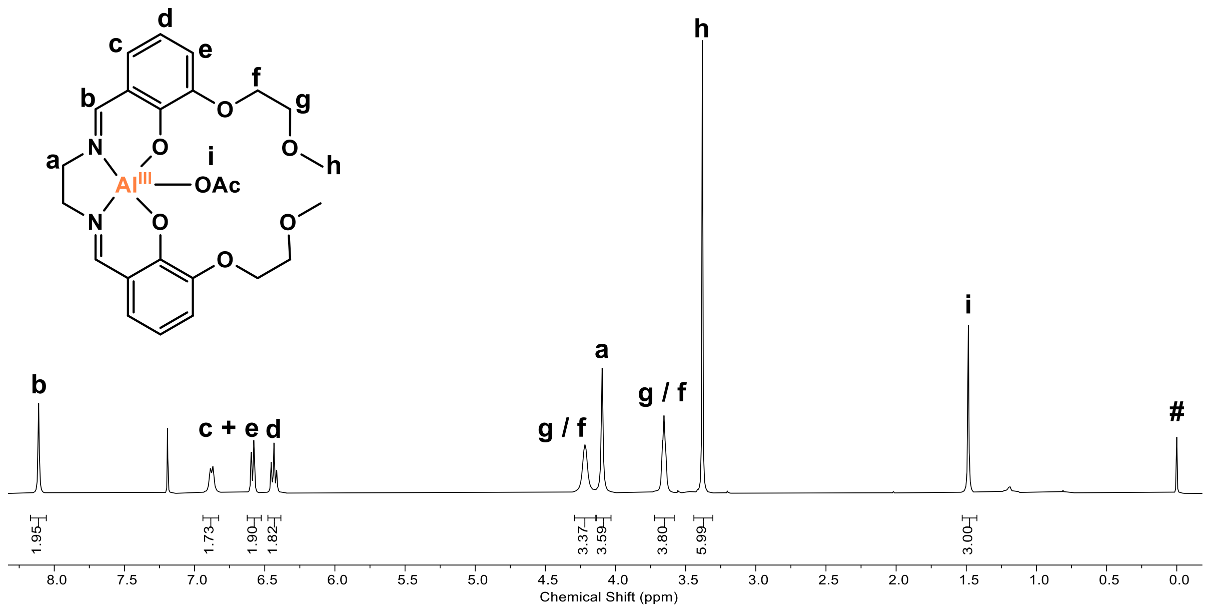

Fig. S11 ^{1}H NMR spectrum of L_1AlOAc in $CDCl_3$. (# Denotes vacuum grease)

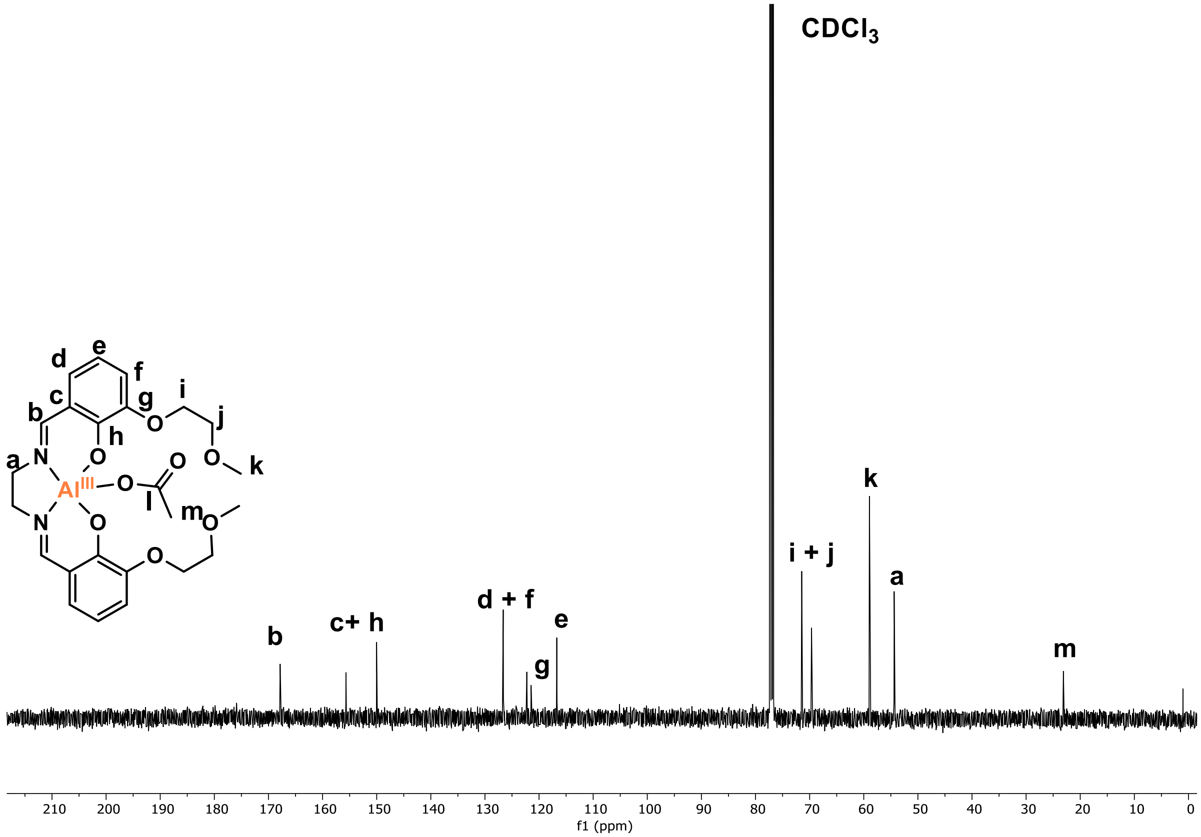

Fig. S12 ^{13}C NMR spectrum of L_1AlOAc in $CDCl_3$.

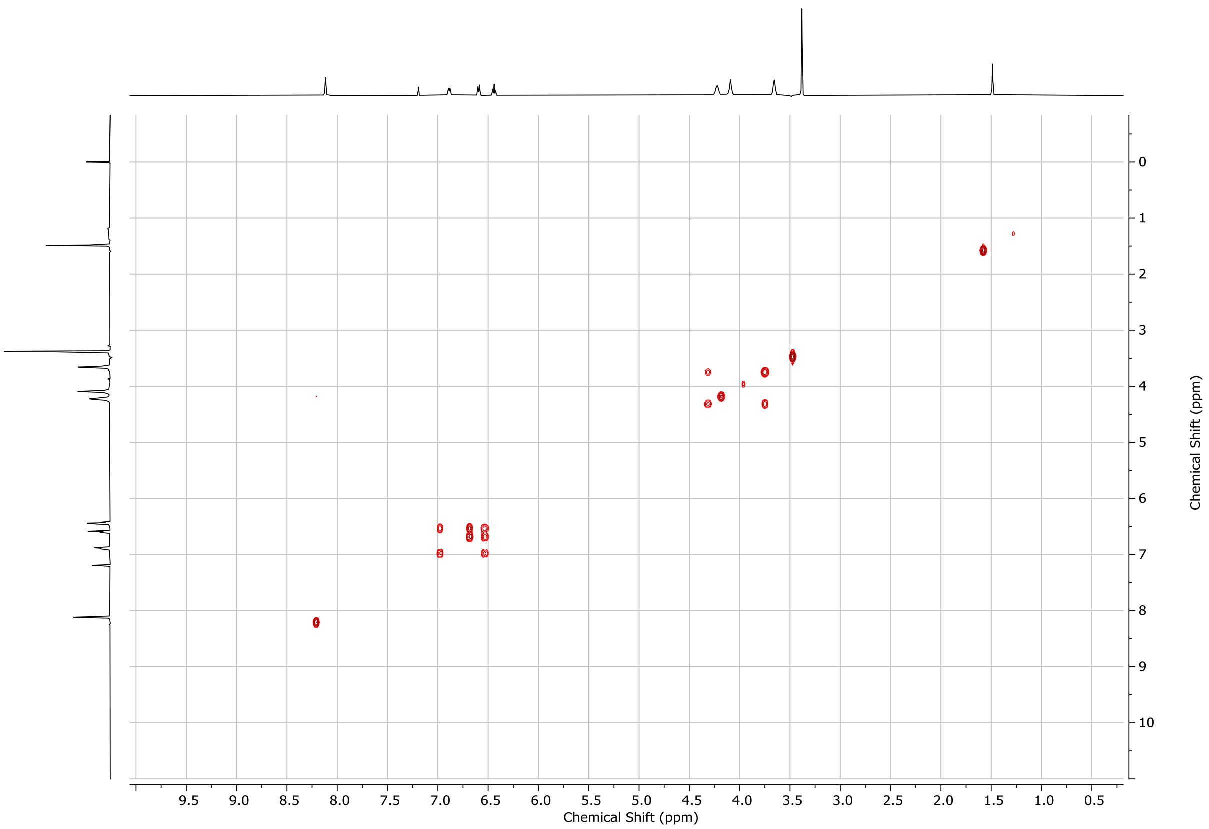

Fig. S13 COSY NMR spectrum of L_1AlOAc in $CDCl_3$.

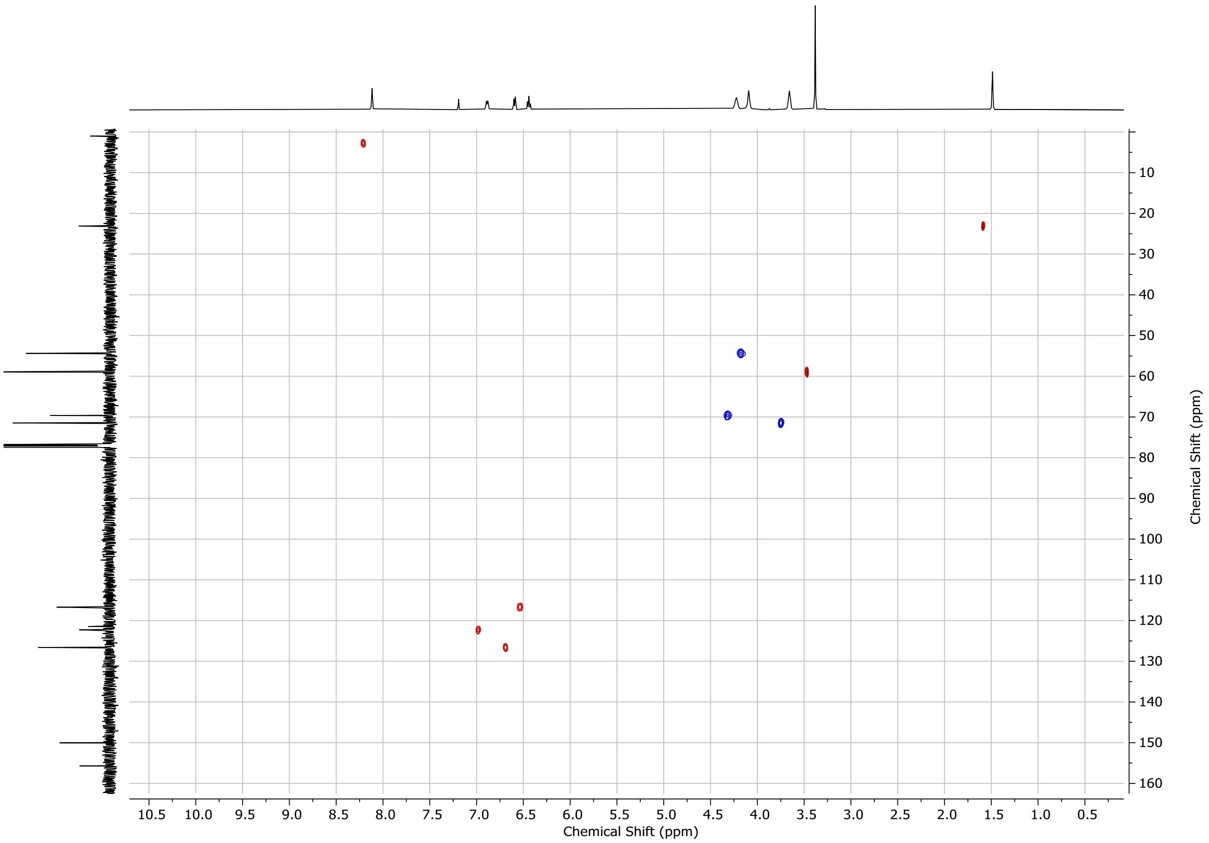

Fig. S14 HSQC NMR spectrum of L_1AlOAc in $CDCl_3$.

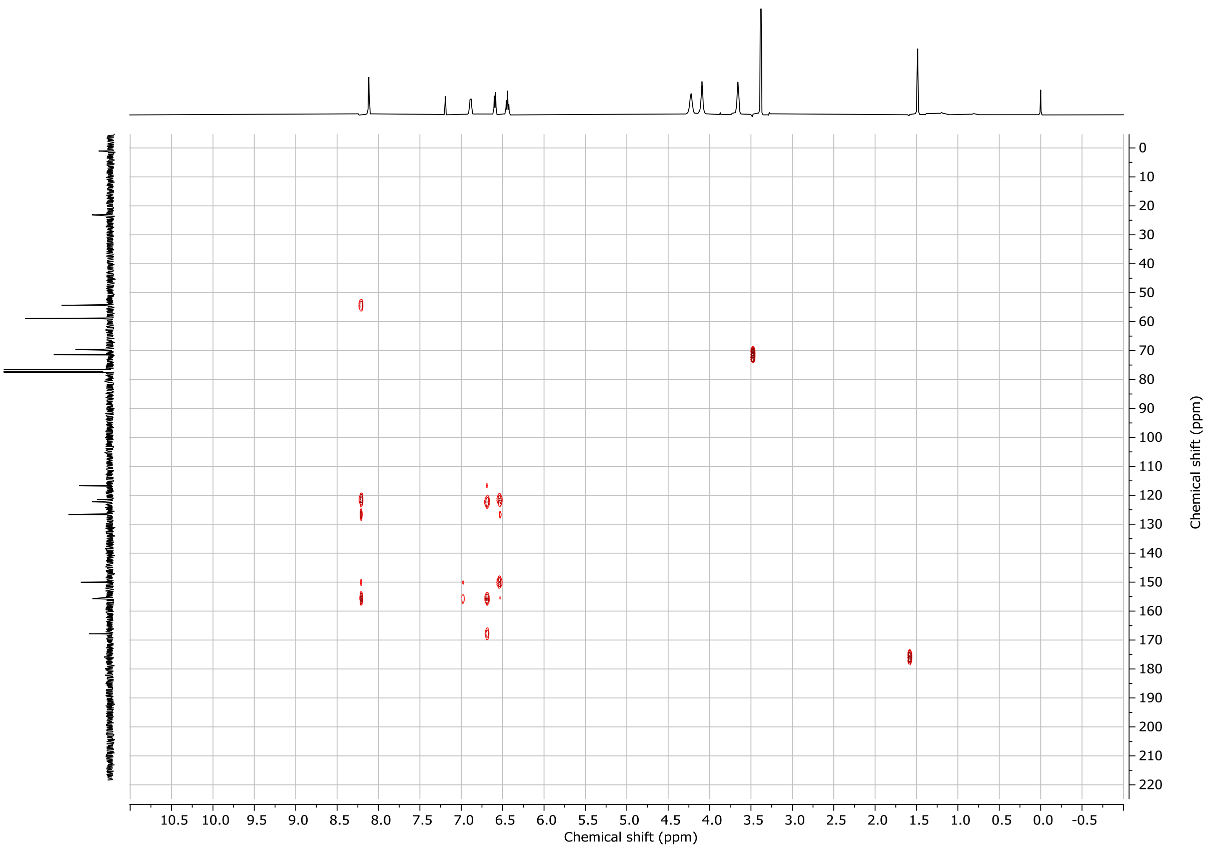

Fig. S15 HMBC NMR spectrum of L_1AlOAc in $CDCl_3$.

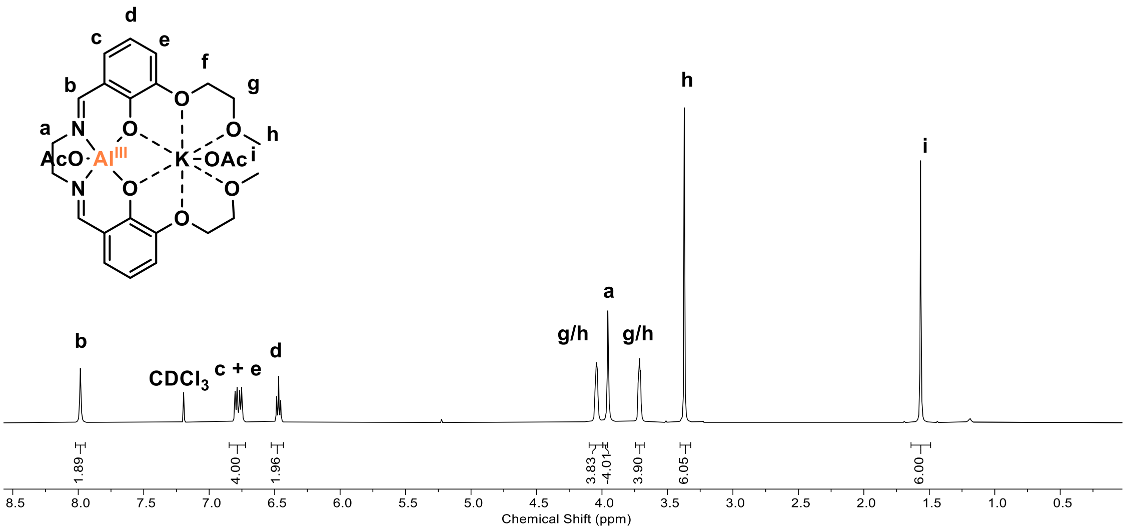

Fig. S16 ^{1}H NMR spectrum of 1 in $CDCl_3$.

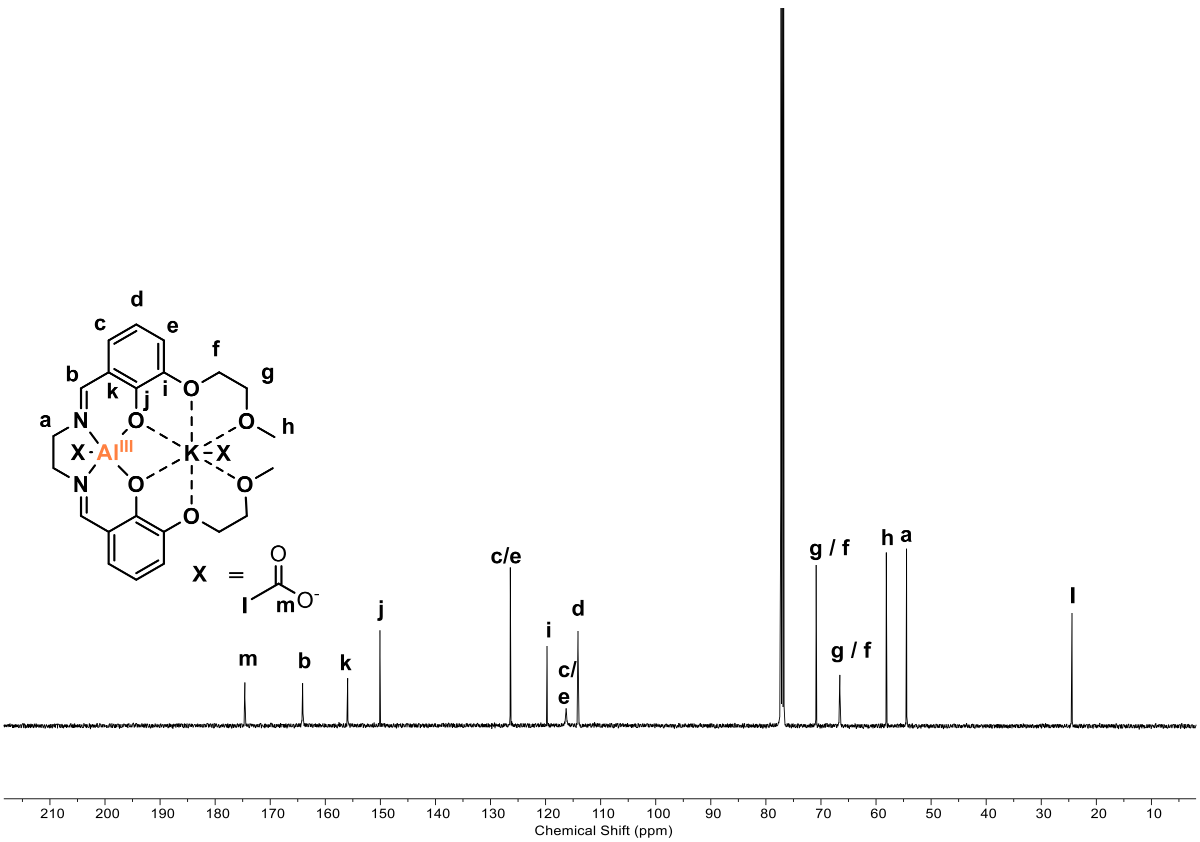

Fig. S17 ^{13}C NMR spectrum of 1 in $CDCl_3$.

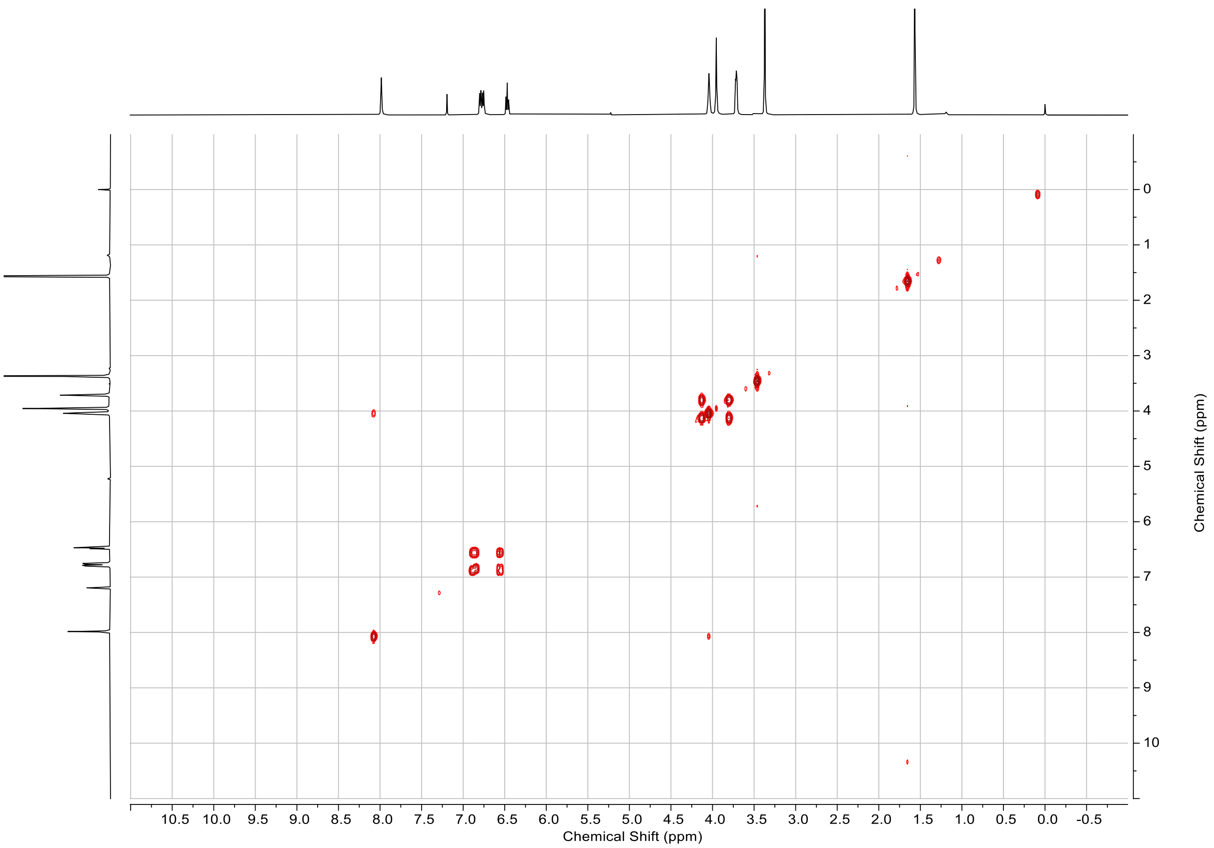

Fig. S18 COSY NMR spectrum of 1 in $CDCl_3$.

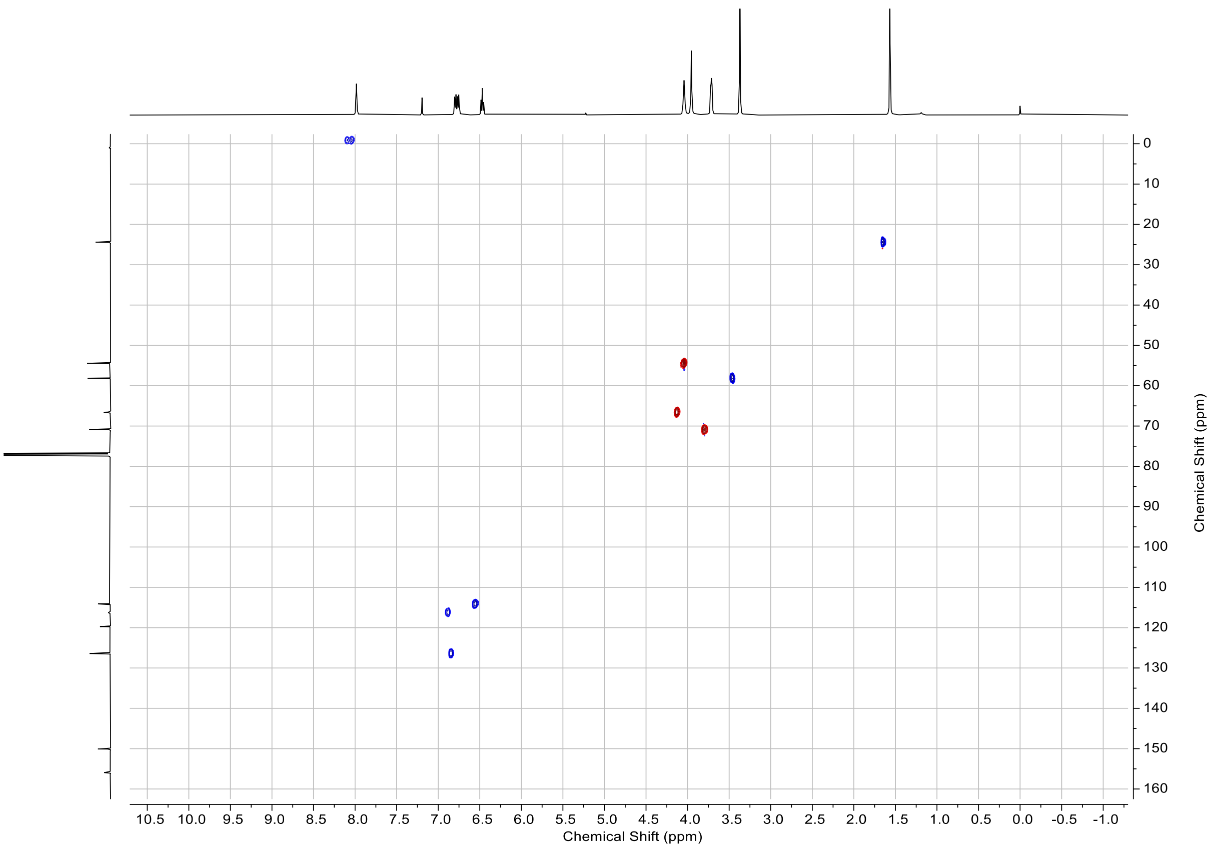

Fig. S19 HSQC NMR spectrum of 1 in $CDCl_3$.

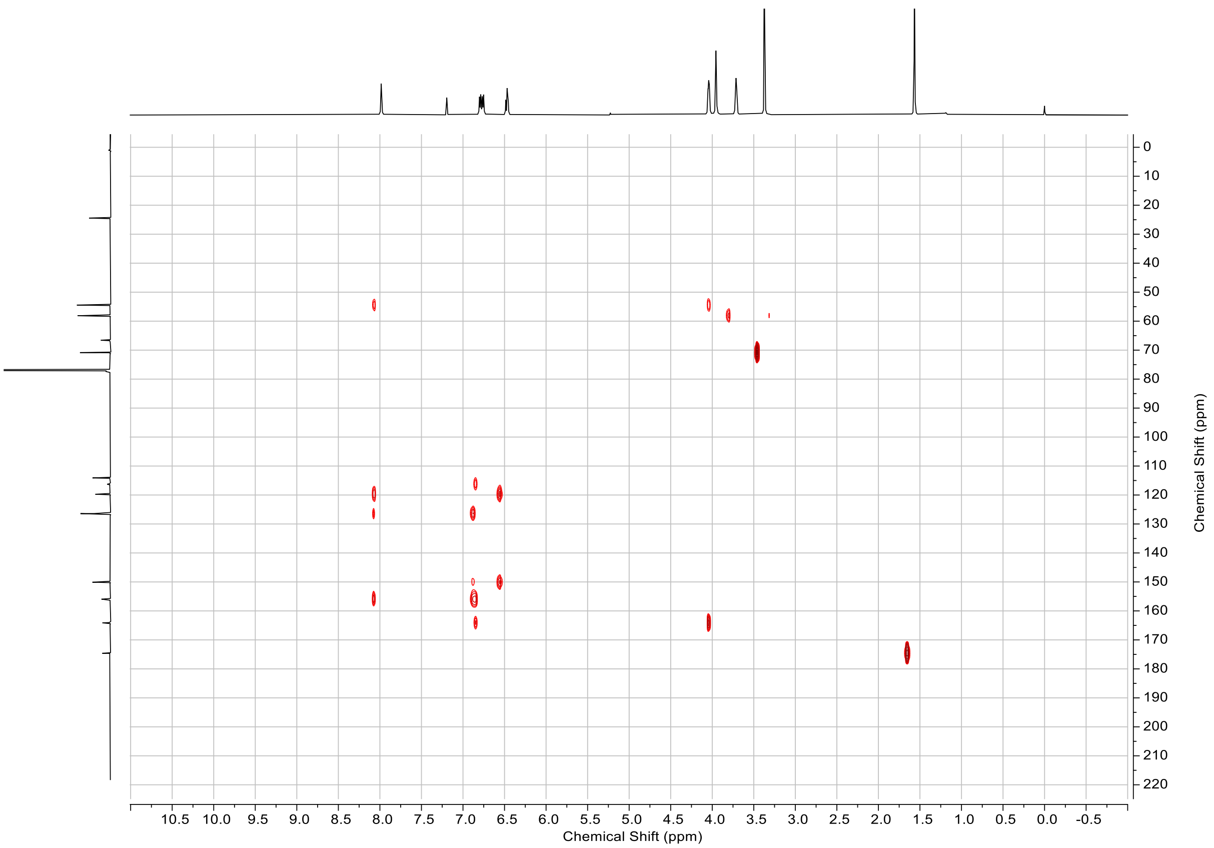

Fig. S20 HMBC NMR spectrum of 1 in $CDCl_3$.

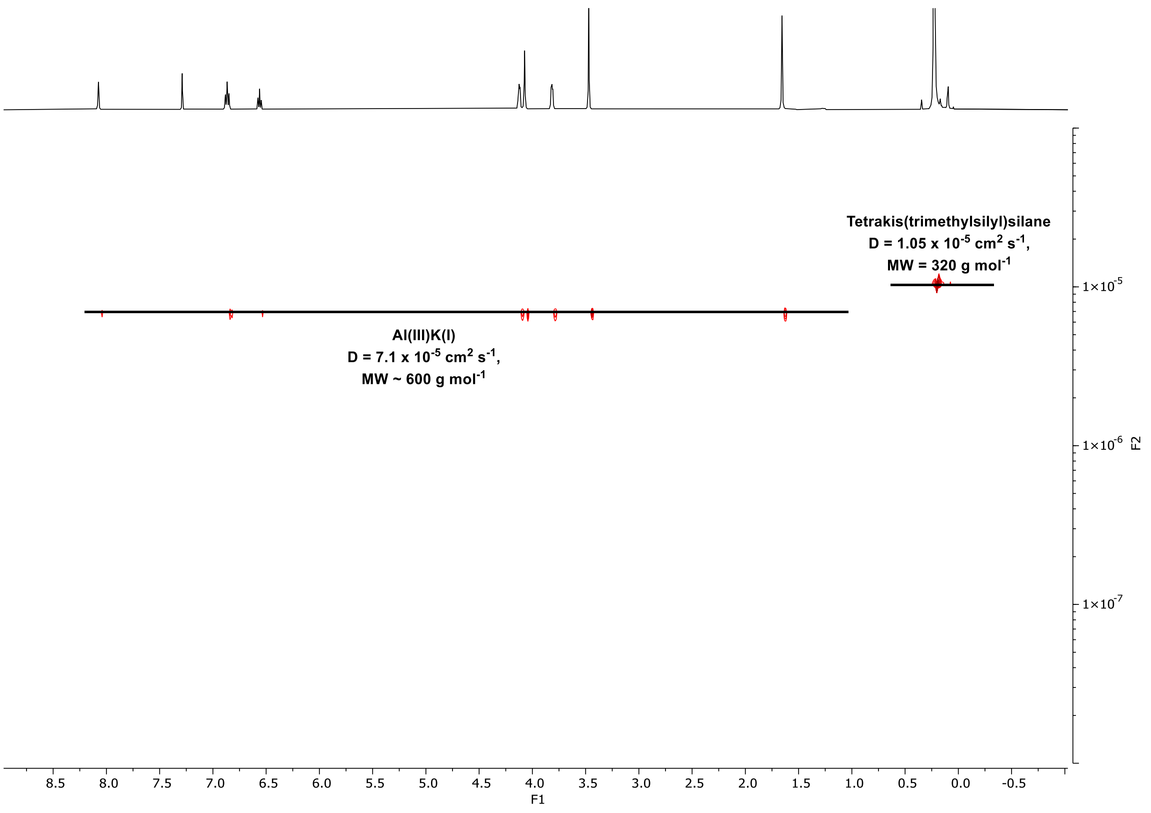

Fig. S21 DOSY spectrum of 1 in $CDCl_3$ with Tetrakis(trimethylsilyl)silane.

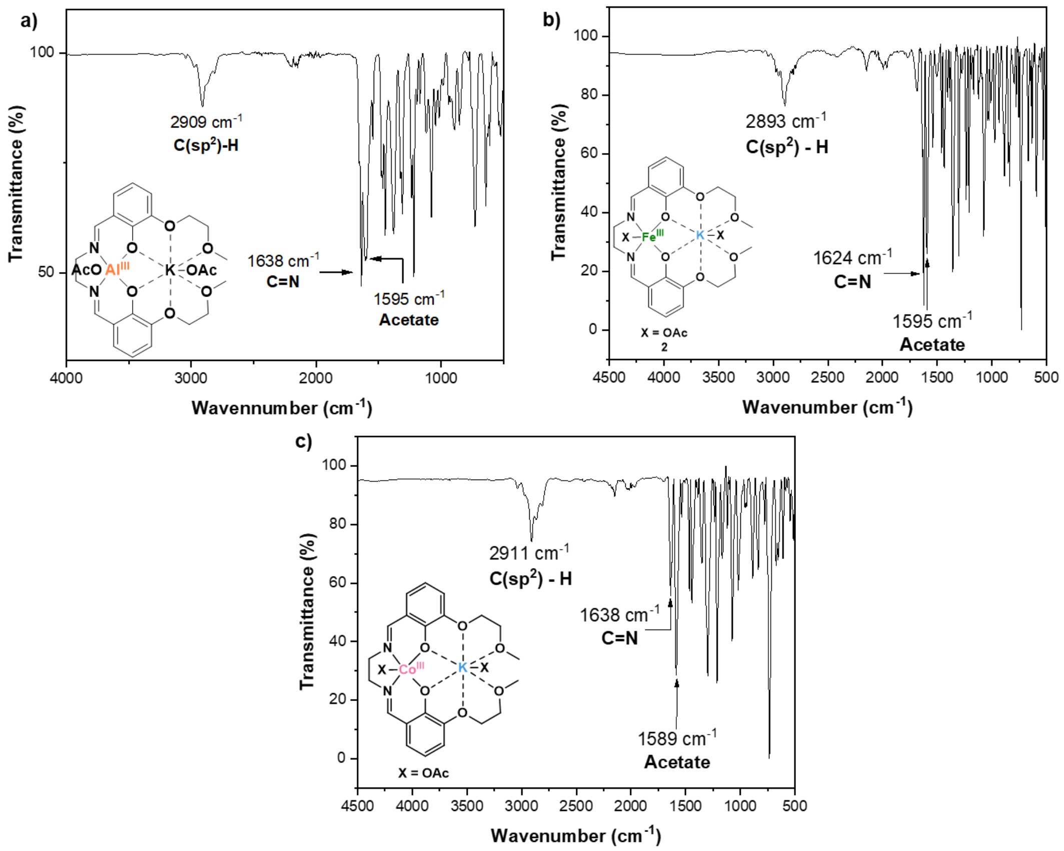

Fig. S22 IR spectra of complexes a) Al(III)K(I) (1), b) Fe(III)K(I) (2) and c) Co(III)K(I) (3).

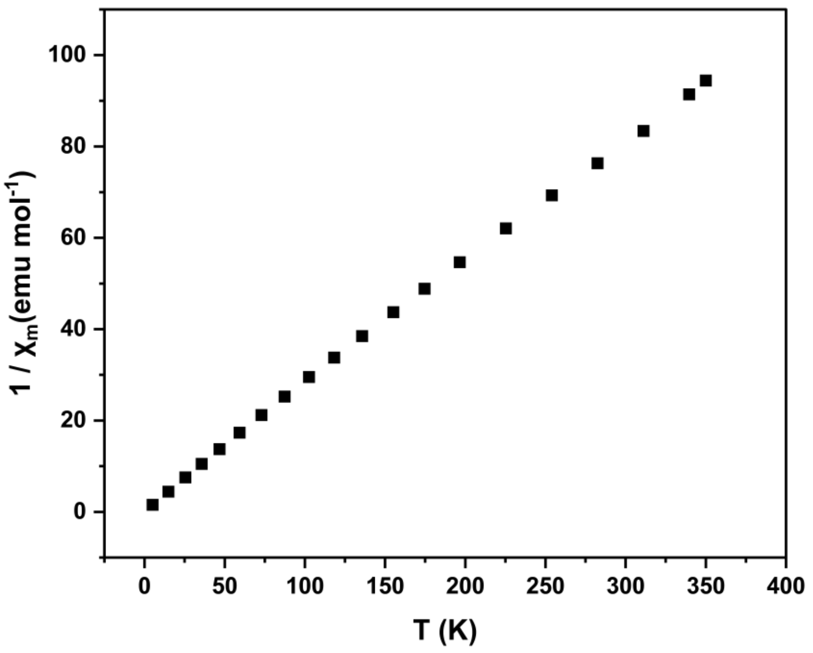

Fig. S23 Plot of χ mol^{-1} *vs*. temperature for complexes 2 obtained via SQUID magnetometry.

Table S1. Magnetometry data for complexes 2.

**Complex**	**C[a] (emu K mol-1)**	**Θ[b] (K)**	**μ_{eff}[c]**	**S[d]**	**μ_{SO}[e]**
2	3.75	5.72	5.46	5/2	5.92

[a] Curie constant is extracted from a plot of χ mol^{-1} vs. temperature (Figure S20). From a linear fit of the data $\chi\ mol^{-1} = \alpha T + \beta,\ C = 1/\alpha$. [b] Weiss constant is extracted from a plot of χ mol^{-1} vs. temperature (Figure S20). From a linear
fit of the data, $\chi\ mol^{-1} = \alpha T + \beta,\ \theta = x$ intercept. [c]Experimentally determined effective magnetic moment; where
$\mu\ e_{ff}$ = 2.828 √(χmT), where $\chi = \frac{C}{T-\theta}$, T = 350 K. [d]Total spin quantum number, assuming high spin M(III) complexes.[e] Theoretical magnetic moment calculated from spin angular momentum only $\mu_{SO} = 2\sqrt{S(S+1)}$, which assumes complete quenching of d orbital angular momentum.

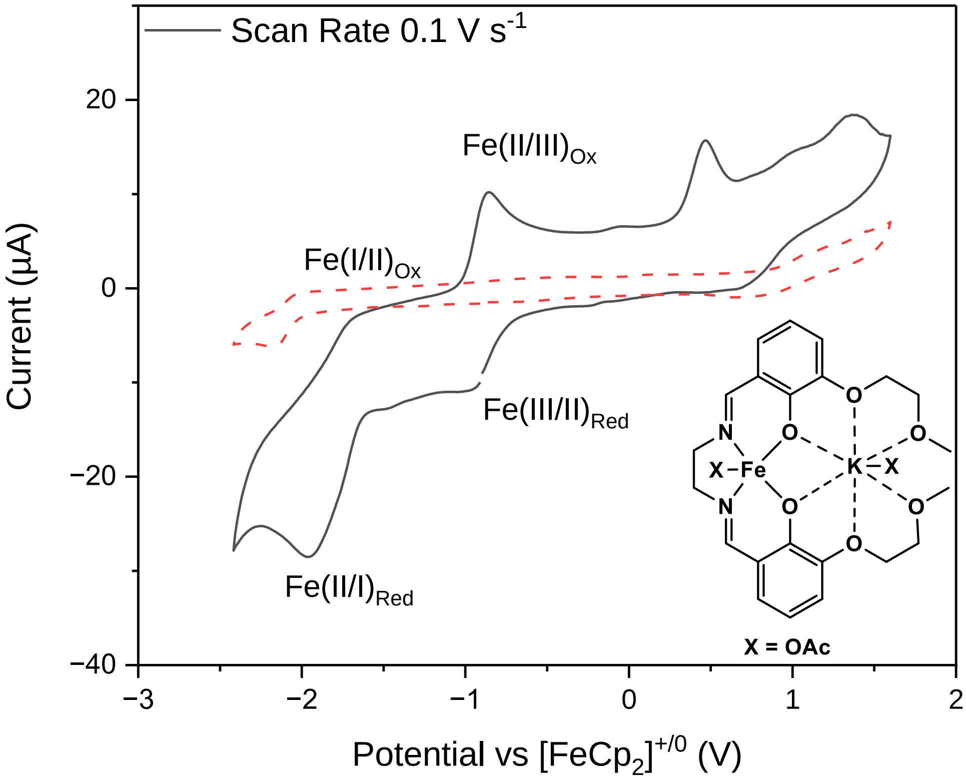

Fig. S24 Cyclic Voltammogram of 2 in MeCN, where Fe(III/II)$_{1/2}$ = - 0.94 V. The red trace shown is the electrolyte (0.1 M of tetrabutylammonium hexafluorophosphate) only.

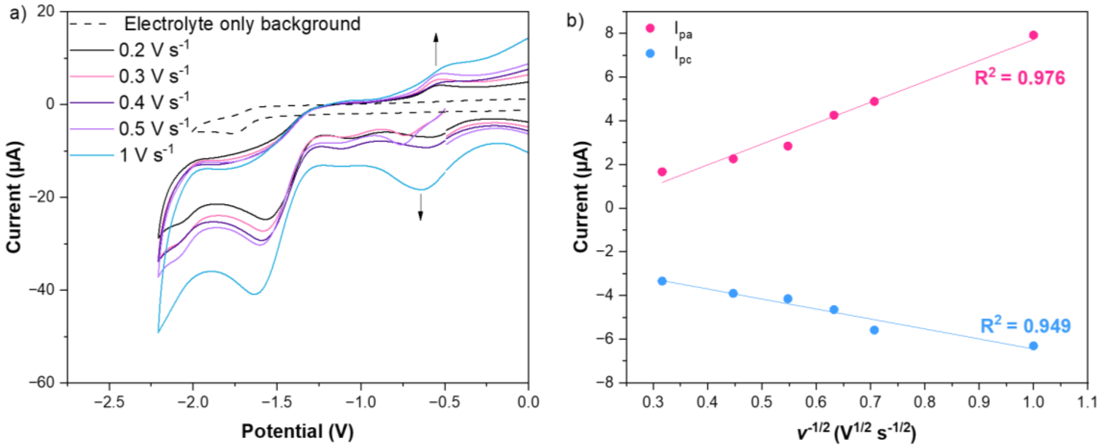

Fig. S25 a) Overlay of cyclic voltammograms of (2) obtained at different scan rates, b) Linear relationship between the peak current and square root of the scan rate.

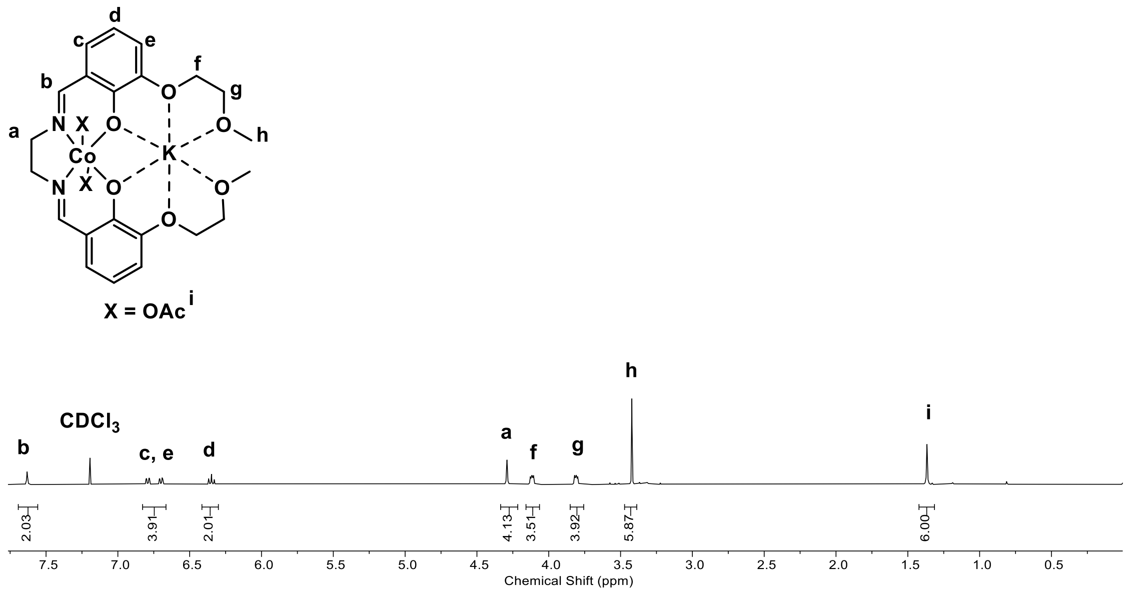

Fig. S26 ^{1}H NMR spectrum of 3 in $CDCl_3$.

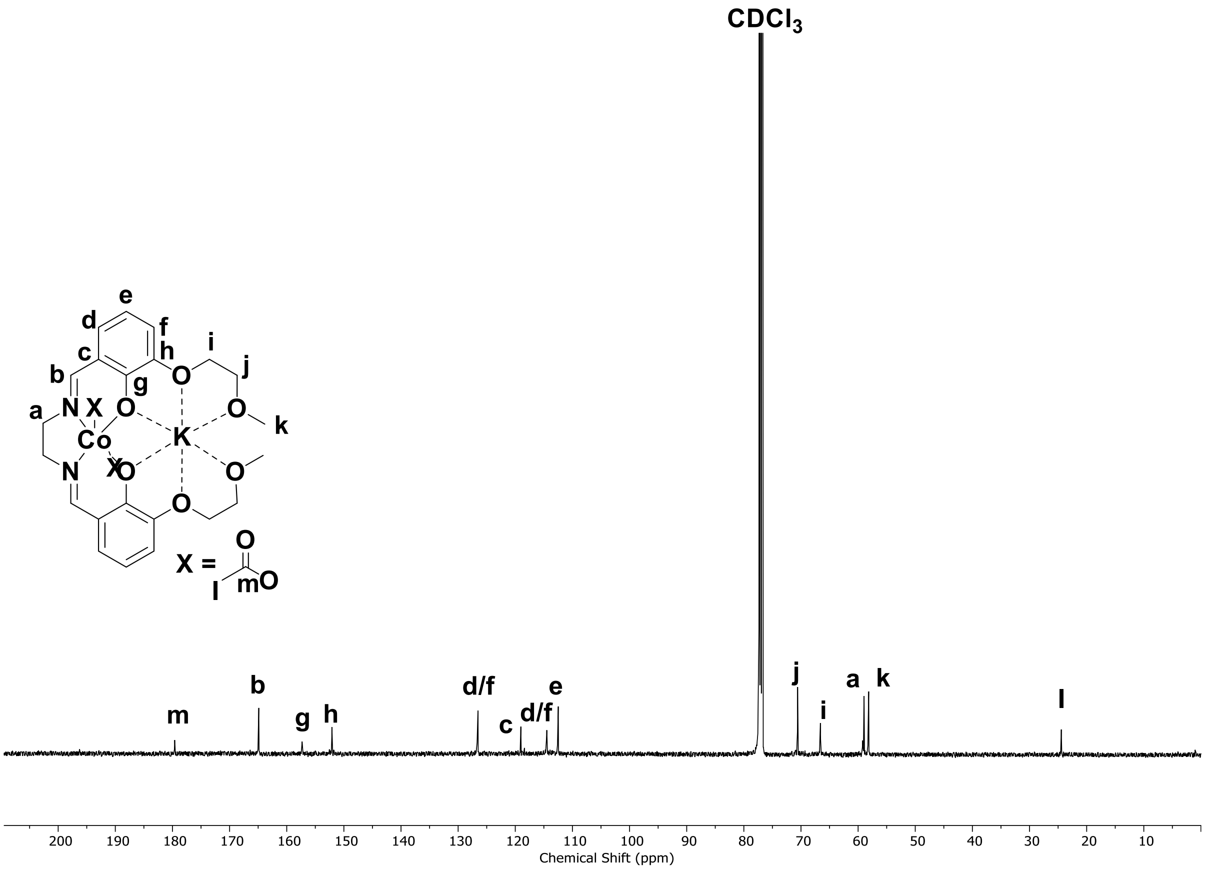

Fig S27 ^{13}C NMR spectrum of 3 in $CDCl_3$.

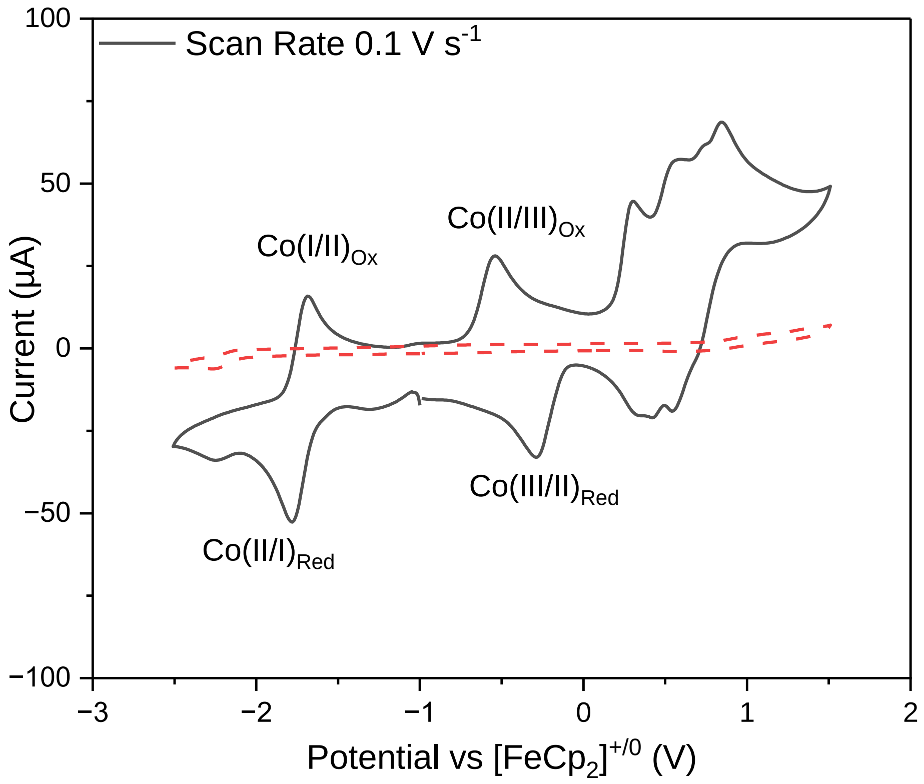

Fig. S28 Cyclic Voltammogram of 3 in MeCN, where Co(III/II)$_{1/2}$ = - 0.46 V. The red trace shown is the electrolyte (0.1 M of tetrabutylammonium hexafluorophosphate) only.

'Greener' Synthesis Design

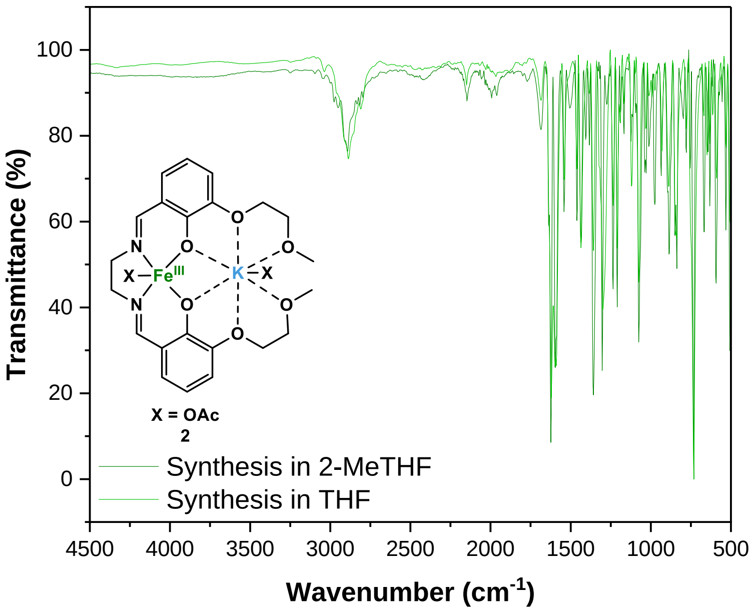

Fig. S29 Overlay of IR spectra of Fe(III)K(I) (2) synthesised in THF and 2-MeTHF.

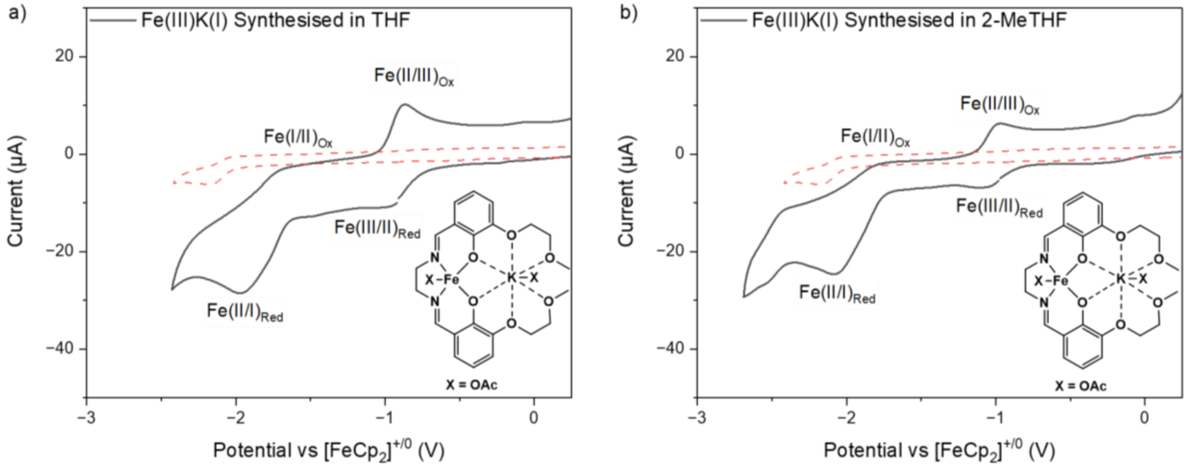

Fig. S30 Comparison CV spectra (1.6 mM Fe(III)K(I) in MeCN, v = 0.1 V) for Fe(III)K(I) (2) synthesised a) in THF, where E1/2 Fe(III/II) = - 0.94 V, and b) in 2-MeTHF, where E1/2 Fe(III/II) = - 0.97 V. The red trace shown is the electrolyte (0.1 M of tetrabutylammonium hexafluorophosphate) only.

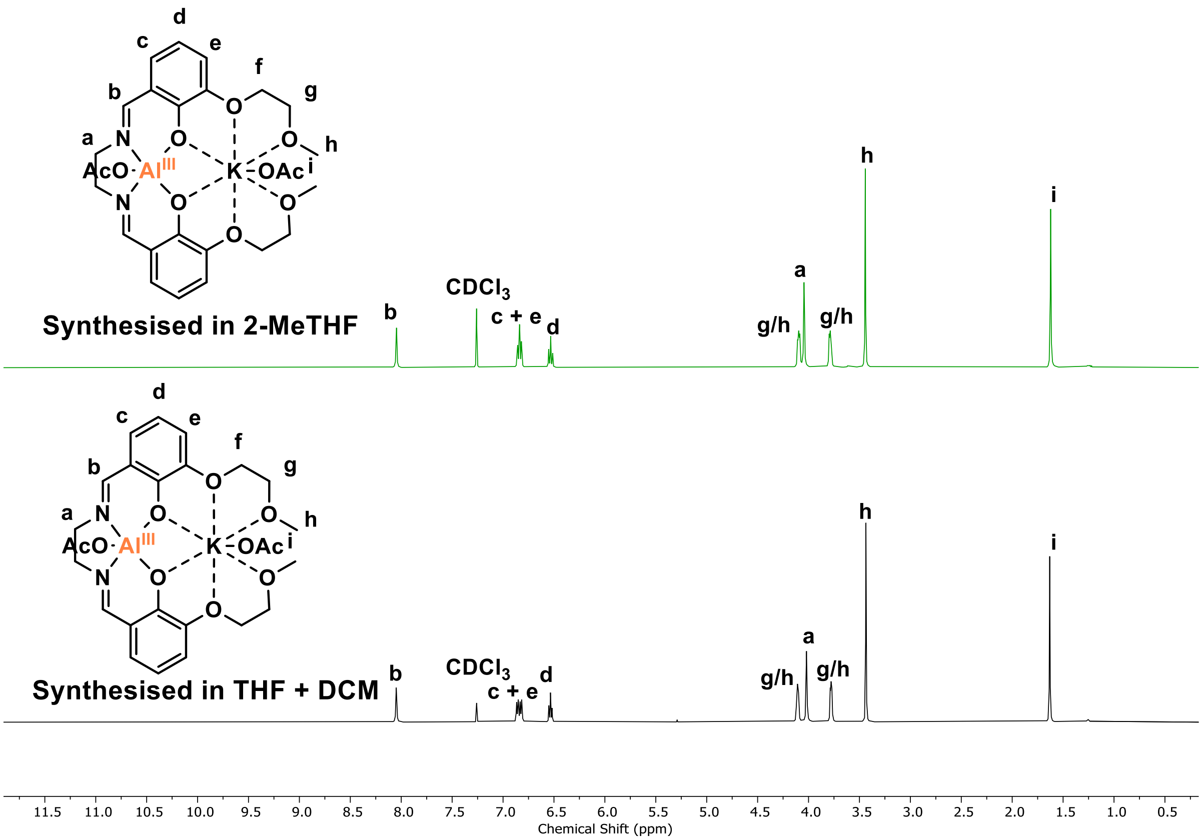

Fig. S31 Comparison of ^{1}H NMR spectra of Al(III)K(I) in $CDCl_3$ synthesised in 2-MeTHF (top spectrum) and synthesised in THF and DCM (bottom spectrum).

Thermal stability of catalyst 1-3

The thermal stability of catalyst 1-3 was confirmed using thermogravimetric analysis and IR spectroscopy. The stability of each catalyst was tested by under the highest temperature used in any polymerisation and for the duration of the longest polymerisation run. The structural stability of each catalyst was confirmed by comparison of the IR spectrum before and after heating.

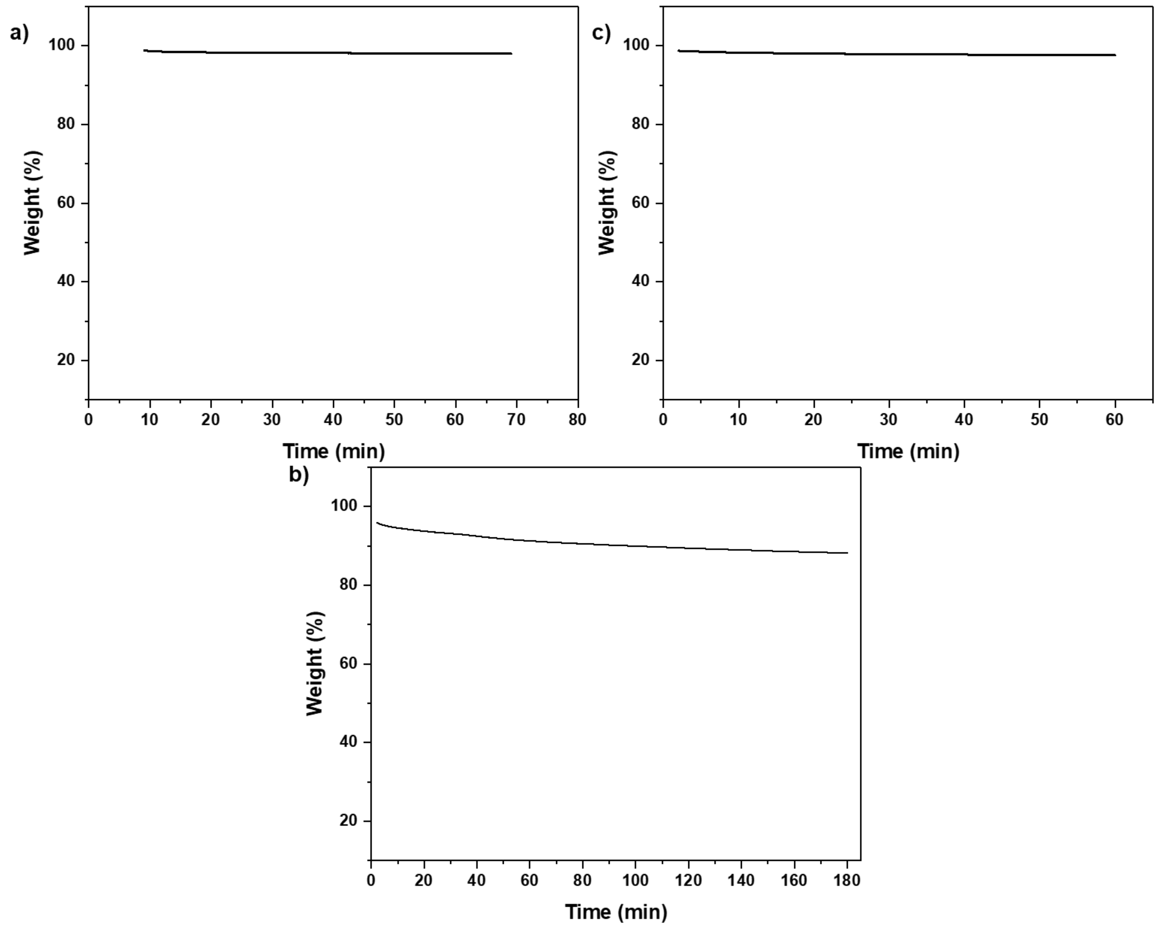

Fig. S32 Heat stability of 1-3 a) TGA plot of Al(III)K(I) (1) mass over time, obtain by holding an isotherm at 120 °C for 1 h. c) TGA plot of Fe(IIIK(I) (2) mass over time, obtain by holding an isotherm at 140 °C for 3 h. e) TGA plot of Co(III)K(I) (3) mass over time, obtain by holding an isotherm at 100 °C for 1 h.

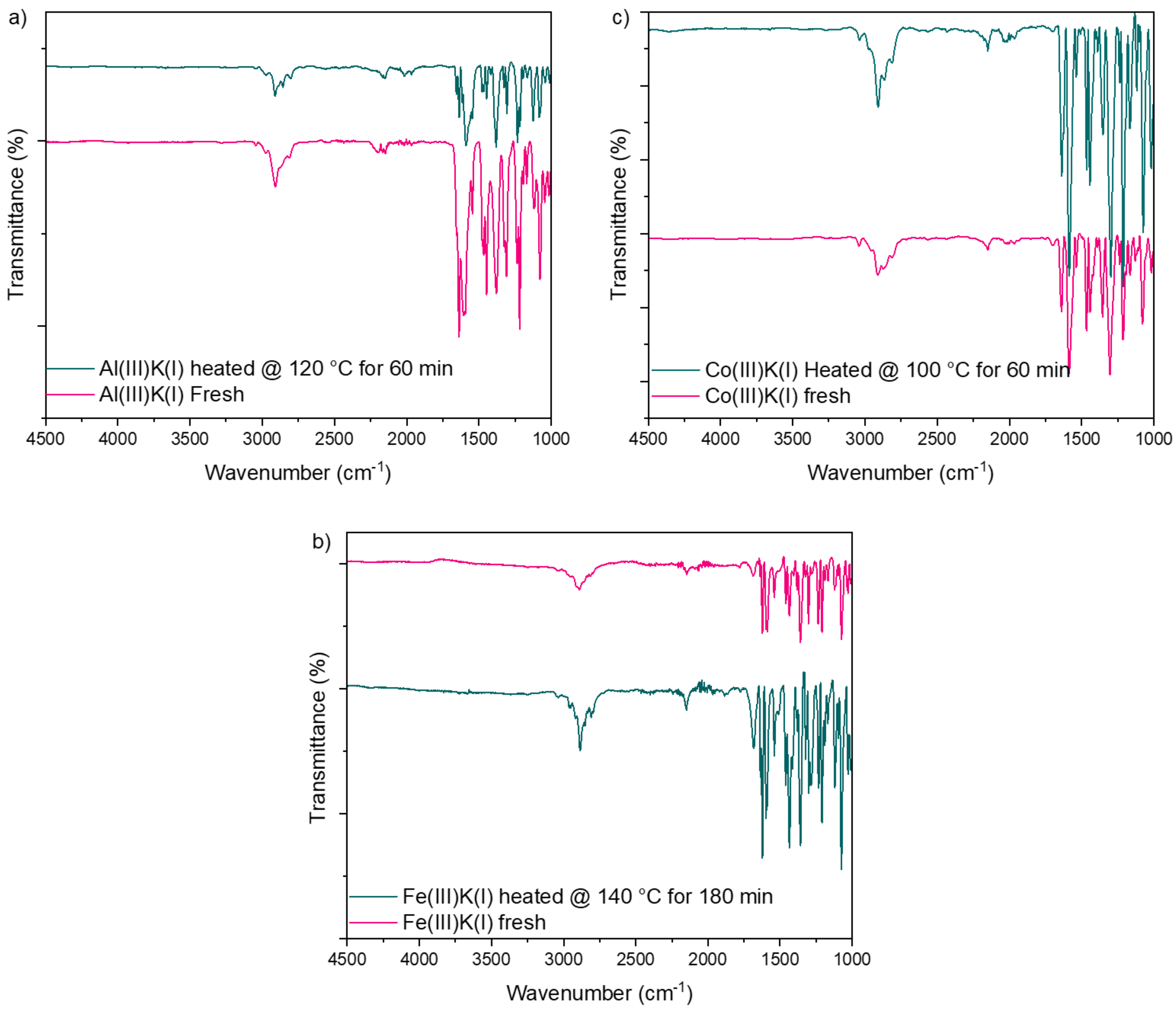

Fig. S33 Heat stability of 1-3 IR spectra a) Overlay of the IR spectrum of Al(III)K(I) before and after heating at 120 °C for 1 h. b) Overlay of the IR spectrum of Fe(III)K(I) before and after heating at 140 °C for 3 h. c) Overlay of the IR spectrum of Co(III)K(I) before and after heating at 100 °C for 1 h.

Table S2 Key IR stretches before and after heating experiments (IR spectra shown in Fig. S33)

Complex	C(sp^2-H) stretch	C=N stretch	Acetate stretch
Co(III)K(I) fresh	2911	1638	1589
Co(III)K(I) heated	2911	1638	1589
Fe(III)K(I) fresh	2893	1624	1595
Fe(III)K(I) heated	2893	1624	1595
Al(III)K(I) fresh	2909	1638	1595
Al(III)K(I) heated	2910	1638	1590

COPASI models

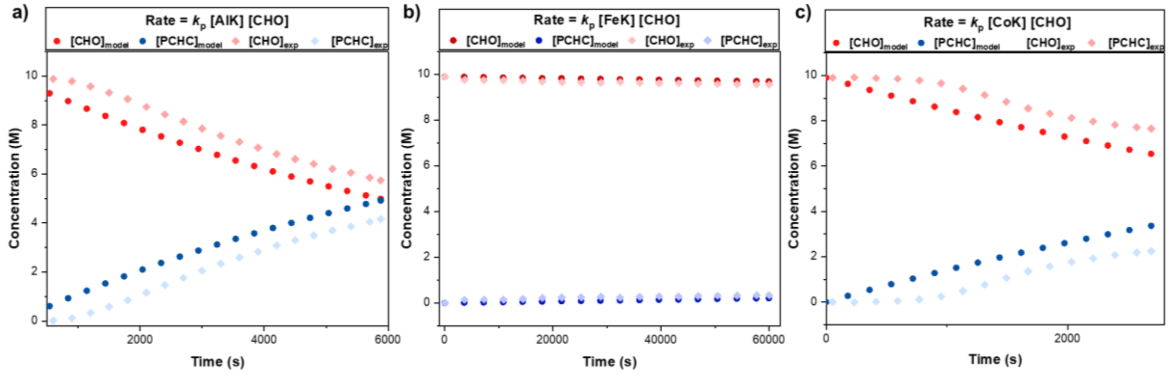

Fig. S34 Concentration vs time data modelled using the previously reported rate law for CHO/CO2 ROCOP in COPASI and comparison with experimentally obtained concentration vs time data for reactions performed with a) Al(III)K(I) (1) and b) Fe(III)K(I) (2) c) Co(III)K(I) (3) (Table 2).

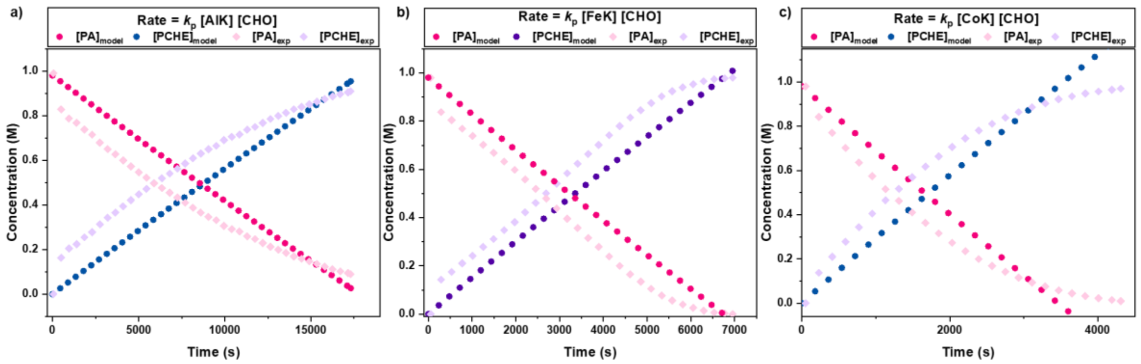

Fig. S35 Concentration vs time data modelled using the previously reported rate law for CHO/PA ROCOP in COPASI and comparison with experimentally obtained concentration vs time data for reactions performed with a) Al(III)K(I) (1) and b) Fe(III)K(I) (2) and c) Co(III)K(I) (3) (Table S2).

Table S3 Parameters and rate equations used for COPASI models.

Entry	Model	Reaction(s)	Rate Law(s)	Parameters
1	CHO/CO_2 ROCOP using Al(III)K(I) (1)	Catalyst + CHO → PCHC + Catalyst	k_p*Catalyst*CHO	Catalyst = 0.0025 mol l^{-1}, fixed; CHO =9.9 mol l^{-1}, reactions; PCHC = 0 mol l^{-1}, reactions; k_p = 0.0467 l^2 mol^{-2} s^{-1}, fixed
2	CHO/CO_2 ROCOP using Fe(III)K(I) (2)	Catalyst + CHO → PCHC + Catalyst	k_p*Catalyst*CHO	Catalyst = 0.0025 mol l^{-1}, fixed; CHO =9.9 mol l^{-1}, reactions, fixed; PCHC = 0 mol l^{-1}, reactions; k_p = 0.0467 l^2 mol^{-2} s^{-1}, fixed
3	CHO/CO_2 ROCOP using Co(III)K(I) (3)	Catalyst + CHO → PCHC + Catalyst	k_p*Catalyst*CHO	Catalyst = 0.0025 mol l^{-1}, fixed; CHO =9.9 mol l^{-1}, reactions, fixed; PCHC = 0 mol l^{-1}, reactions; k_p = 0.0616 l^2 mol^{-2} s^{-1}, fixed
4	CHO/PA ROCOP using Al(III)K(I) (1)	Catalyst + CHO + PA → PCHE + Catalyst	k_p*Catalyst*CHO	Catalyst = 0.00245 mol l^{-1}, fixed; CHO =9.9 mol l^{-1}, reactions, PA = 0.98 mol l^{-1}, fixed; PCHC = 0 mol l^{-1}, reactions; k_p = 0.0012 l^2 mol^{-2} s^{-1}, fixed
5	CHO/PA ROCOP using Fe(III)K(I) (2)	Catalyst + CHO + PA → PCHE + Catalyst	k_p*Catalyst*CHO	Catalyst = 0.00245 mol l^{-1}, fixed; CHO =9.9 mol l^{-1}, reactions, PA = 0.98 mol l^{-1}, fixed; PCHC = 0 mol l^{-1}, reactions; k_p = 0.00631 l^2 mol^{-2} s^{-1}, fixed
6	CHO/PA ROCOP using Co(III)K(I) (3)	Catalyst + CHO + PA → PCHE + Catalyst	k_p*Catalyst*CHO	Catalyst = 0.00245 mol l^{-1}, fixed; CHO =9.9 mol l^{-1}, reactions, PA = 0.98 mol l^{-1}, fixed; PCHC = 0 mol l^{-1}, reactions; k_p = 0.0123 l^2 mol^{-2} s^{-1}, fixed

Polymerisation data

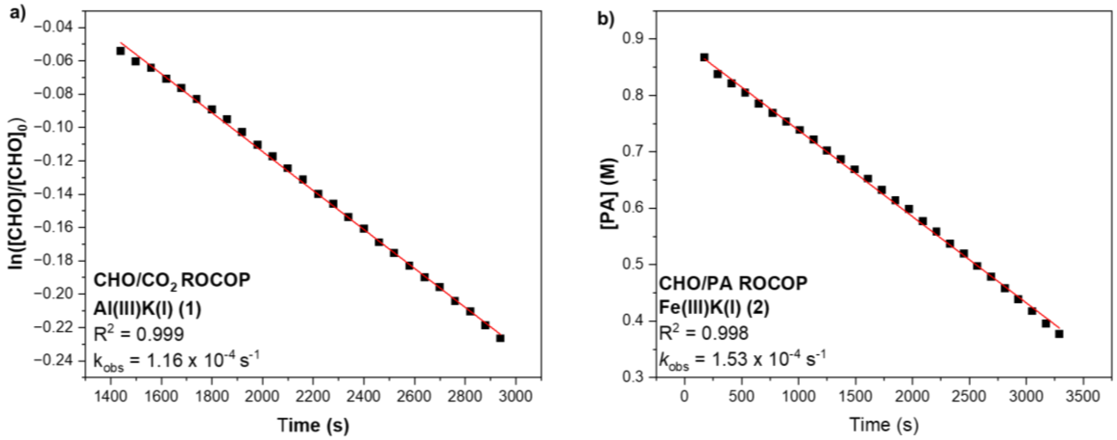

Fig. S36 a) Exemplary, linear semilogarithmic plot of ln([CHO]/[CHO]$_0$) vs time between 5-20 % epoxide conversion, used to determine k_{obs} for CHO/CO_2, b) a) Exemplary, linear plot of [PA] vs time between 10-60% PA conversion, used to determine k_{obs} for CHO/PA.

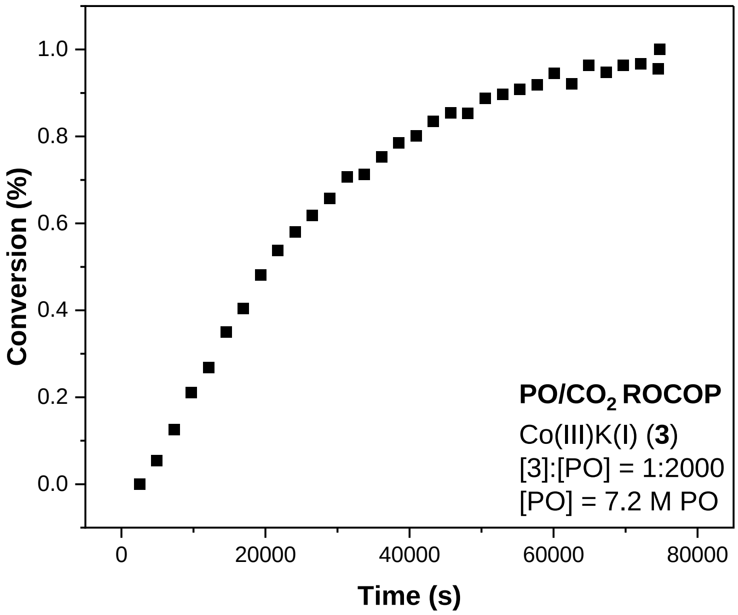

Fig. S37 Conversion over time data for the polymerisation of PO/CO_2 ROCOP using Co(III)K(I) (3), illustrating that full conversion can be reached when using dilute reaction conditions: 1:20:2000 = [cat]:[CHD]:[PO], 20 bar CO_2, 50 °C, in 6 mL, diluted with dimethyl carbonate to [PO] = 7.2 M.

Table S4 Conversion data for the epoxide/CO2 ROCOP reactions reported in Table 1, showing how the TON and TOF were calculated

#	Catalyst	Epoxide	Time / h	Conversion / %[c]	Productivity TON [d]	TOF (h^{-1})[f]
1	Al(III)K(I) (1)	CHO[a]	1.5	25, 24	1405 ± 180	1083 ± 68
2		PO[b]	50	7, 8	314 ± 13	6 ± 0.7
3	Fe(III)K(I) (2)	CHO[a]	26	12, 12	471 ± 10	19 ± 2
4		PO[b, i]	23	2, 1	44 ± 8	1.5 ± 0.4
5	Co(III)K(I) (3)	CHO[a]	0.75	23, 28	1023 ± 81	1226 ± 11
6		PO[b7]	1.5	-	-	833 ± 76

Reaction conditions: [a] $[cat]_0$:[*trans*-1,2-cyclohexanediol]$_0$:$[CHO]_0$ = 1:20:4000, in 6 mL, neat CHO (9.9 M), under 20 bar CO_2 pressure, 100 °C. $[cat]_0$:[*trans*-1,2-cyclohexanediol]$_0$:$[PO]_0$ = 1:20:4000, in 6 mL neat PO (14.3 M), under 20 bar CO_2 pressure, 60 °C. [c] Overall epoxide conversion (PO or CHO: determined by comparison of the sum of integrals by ^1H NMR spectroscopy of polycarbonate (PPC 4.9 ppm, 1 H; PCHC 4.6 ppm, 2 H) and cyclic carbonate (PC 4.8 ppm, 1H; CC 4.8 ppm 2H) and polyether (3.46-3.64 ppm, 3H for PPO and 4 H for PCHO) against mesitylene (6.7 ppm) as an internal standard, conversions for each run are reported, as conversion run 1, conversion run 2. [d]TON was determined by dividing the moles of epoxide consumed / moles of catalyst. [e]Turnover frequency (TOF) was calculated by dividing the Turnover number (TON) against time. All TON and TOF values are reported as an average of n =2 runs, with an error of $\pm\Delta x = \sigma/\sqrt{n}$, conversions are given for each individual run.

Table S5 Conversion data for the epoxide/PA ROCOP reactions reported in Table 2, showing how the TON and TOF were calculated.

#	Catalyst	Epoxide	Time / h	Conversion / %[c]	Productivity TON [d]	TOF /h^{-1}[e]
1	Al(III)K(I) (1)	CHO[a]	4	90, 71	330 ± 24	78 ± 5
2		PO[b]	17	55, 47	204 ± 11	12 ±0.7
3	Fe(III)K(I) (2)	CHO[a]	2	98, 100	410 ± 7	196 ± 10
4		PO[b]	11	43, 28	99 ± 9	9 ± 1
5	Co(III)K(I) (3)	CHO[a]	1	99, 100	404 ± 2	428 ± 15
6		PO[b]	7	100, 100	401 ± 1	66 ± 7

Reaction conditions: [a]$[cat]_0$:[*trans*-1,2-cyclohexanediol]$_0$:$[PA]_0$:$[CHO]_0$ = 1:20:400:4000, in 3 mL, neat CHO (9.9 M), 100 °C. $[cat]_0$:[*trans*-1,2-cyclohexanediol]$_0$:$[PA]_0$;$[PO]_0$ = 1:20:400:4000, in 3 mL neat PO (14.3 M), 60 °C. [c]Absolute conversion determined by ^1H NMR through comparison of resonances associated with PA (8.10–7.85 ppm) and PCHPE or PPE(7.65–7.30 ppm). Conversions for each run are reported, as conversion run 1, conversion run 2. [d] Turnover number (TON) for polyester formation = number of moles of PA consumed/number of moles of the catalyst. [e]Turnover frequency (TOF) was calculated by dividing the Turnover number (TON) against time,. [f]$k_p = k_{obs} / [cat]_0$, where k_{obs} was determined as the gradient of the plot of $\ln[epoxide]_t/[epoxide]_0$ *vs* time. All TON and TOF values are reported as an average of n =2 runs, with an error of $\pm\Delta x = \sigma/\sqrt{n}$.

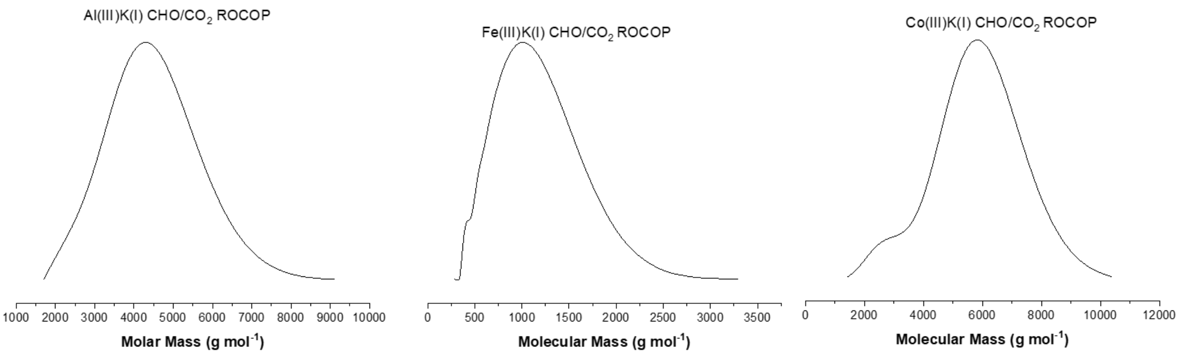

Fig. S38 Representative GPC traces for Table 1, #1, #3, #4.

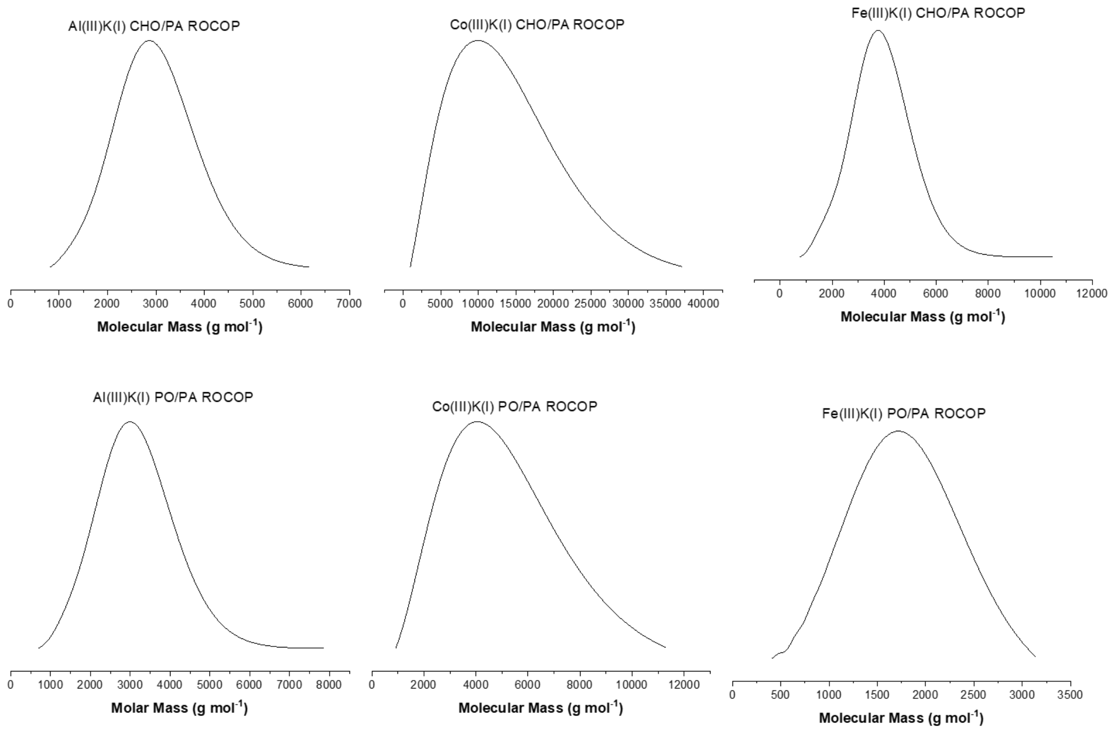

Fig. S39 Representative GPC traces for Table 2, #1-#6.

Approximating catalyst activity per cost

Table S6 Activities and prices considered in the cost estimate (Figure 4).

Catalyst	Monomers	TOF (h^{-1})[a]	Metal precursor price / 1g (£)[b]	Solvent price / 100 mL (£)[c]	Yield (%)[d]	(i) TOF / price (£)[e]	(ii) TOF / price (£)[f]	(iii) TOF / price (£)[g]
Al(III)K(I)	CHO/PA	78	2.04	1.7	80	38.2	31.9	18.8
Co(III)K(I)	CHO/PA	428	18.3	1.4	75	23.4	18.7	17.7
Fe(III)K(I)	CHO/PA	196	9.45	1.7	73	9.61	75.7	45.7
Al(III)K(I)	CHO/CO_2	1083	2.04	1.7	80	531	442	261
Co(III)K(I)	CHO/CO_2	1226	18.3	1.4	75	670	536	506
Fe(III)K(I)	CHO/CO_2	19	9.45	1.7	73	931	46.6	3.29

[a]TOF taken from Table 1-2; [b]Prices used are publicly available catalogue prices for the required metal precursors as follows: Al(III) precursor: $AlEt_3$, from Sigma Aldrich, SKU: 257168-100G, accessed 07/03/2024; Fe(II)OAc_2, , from Fisher Scientific, Thermo Scientific Acros305370100, accessed 07/03/2024; Co(II)OAc_2, from Sigma Aldrich, SKU: 399973-10G, accessed 07/03/2024.[c]Prices used are publicly available catalogue prices for the required solvents as follows: Acetonitrile: from Sigma Aldrich, SKU: 271004-1L, accessed 07/03/2024; 2-MeTHF: from Sigma Aldrich, SKU: 414247-1L, accessed 07/03/2024. [d]Yield from the 'greener' synthesis is considered for Al(III)K(I) and Fe(III)K(I). [e] TOF / price of the metal precursor: calculated as TOF / metal precursor price per 1 g.[f] TOF / price of metal precursor required to obtain 1 g of catalyst, considering the reaction yield, according to: $\frac{\text{TOF}}{\text{Metal precursor price} \times (1+(1-\text{yield}))}$; [g]TOF/ metal precursor price, considering yield and solvent, assuming 100 mL solvent per synthesis, according to: $\frac{\text{TOF}}{\text{Metal precursor price} \times (1+(1-\text{yield}))+\text{solvent price}}$.

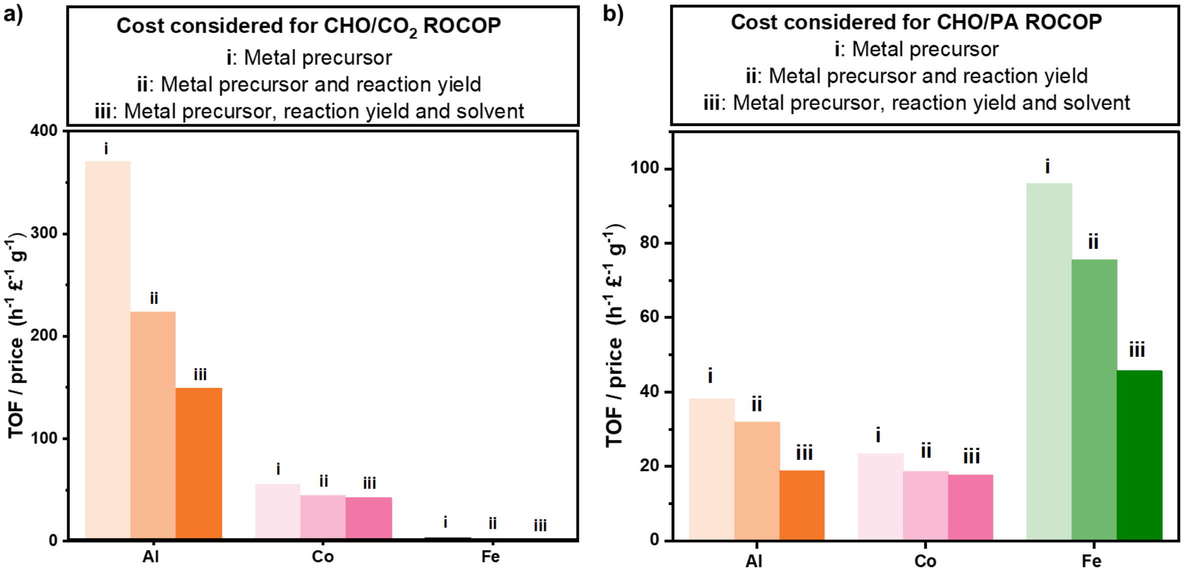

Figure S40 Plots showing the TOF / price (h^{-1} £$^{-1}$ g^{-1}) for (a) the CHO/CO_2 ROCOP and (b) CHO/PA ROCOP. Prices are considered as (1) price of metal precursor required to make one gram of catalyst, (2) Price calculated for (1), taking into account the reaction yield and (3) Price calculated for (2) and additionally taking into account the price of the required solve

Crystallographic Details

The measurement of crystals of Fe(III)K(I) (2) was attempted several times using different instruments and experimental set ups. However, a high level of background fluorescence was observed for all samples, leading to a higher atomic displacement parameter max/min ratio and a high U(eq) for some atoms in the here reported structure.

A solvent mask has been used in the refinement of the Fe(III)K(I) structure to remove residual electron density, which is proposed to be two disordered water molecules. The water molecules are not coordinated to the structure and are therefore removed.

Table S7 Selected bond lengths for Al(III)Et , Al(III)K(I) (1) and Fe(III)K(I) (2).

**Al(III)-Et (007ke23)**		**Al(III)K(I) (022ke23)**		**Fe(III)K(I) (006ke23)**	
**Bond**	**Length (Å)**	**Bond**	**Length (Å)**	**Bond**	**Length (Å)**
Al1—O1	1.8245 (10)	K1—Al1	3.6314 (4)	Fe1—O10	1.933 (12)
Al1—O6	1.8113 (10)	K1—O1	2.7201 (8)	Fe1—O1	2.208 (3)
Al1—N1	2.0172 (12)	K1—O2	2.8069 (8)	Fe1—O5	1.915 (6)
Al1—N2	2.0135 (12)	K1—O6	2.8187 (8)	Fe1—O10	1.933 (12)
Al1—C1	1.9970 (14)	K1—O5	2.9334 (8)	Fe1—O1	2.208 (3)
O1—C9	1.3200 (16)	K1—O8	2.6704 (8)	Fe1—O5	1.915 (6)
O6—C21	1.3155 (16)	K1—O3	2.9801 (8)	Fe1—O4	1.884 (3)
N1—C3	1.2818 (18)	Al1—O9	1.9160 (8)	O10—C13	1.324 (14)
N1—C24	1.4651 (17)	Al1—O7	1.9204 (8)	O10—K1	2.857 (10)
N2—C22	1.2857 (18)	Al1—O1	1.8275 (8)	O1—C1	1.278 (6)
N2—C23	1.4693 (17)	Al1—O6	1.8207 (8)	O9—C12	1.373 (6)
C24—C23	1.524 (2)	Al1—N2	2.0008 (9)	O9—K1	3.160 (12)
		Al1—N1	1.9972 (9)	O5—K1	2.826 (8)
		N2—C25	1.4644 (13)	O4—C3	1.284 (3)
		N1—C24	1.4673 (14)	O2—C1	1.233 (6)
		C24—C25	1.5295 (15)	O2—K1	2.603 (4)
		O9—C3	1.2826 (13)	O3—C3	1.234 (4)
		O7—C1	1.2820 (13)	O3—K1	2.790 (4)
		O8—C1	1.2337 (14)	N2—C7	1.231 (16)
		C3—C4	1.5162 (16)	N2—C6	1.427 (7)

Table S8 Selected bond angles for Al(III)Et , Al(III)K(I) (1) and Fe(III)K(I) (2).

**Al(III)-Et (007ke23)**		**Al(III)K(I) (022ke23)**		**Fe(III)K(I) (006ke23)**	
**Bond**	**Angle (°)**	**Bond**	**Angle (°)**	**Bond**	**Angle (°)**
O1—Al1—N1	87.10 (5)	O1—K1—Al1	29.193 (16)	O10—Fe1—O1	89.0 (3)
O1—Al1—N2	151.43 (5)	O1—K1—O2	55.98 (2)	O10—Fe1—N2	90.0 (4)
O1—Al1—C1	108.32 (6)	O1—K1—O6	57.37 (2)	O10—Fe1—N1	166.4 (4)
O6—Al1—O1	88.04 (4)	O1—K1—O3	102.16 (2)	O10—Fe1—K1	52.3 (3)
O6—Al1—N1	144.31 (5)	O2—K1—Al1	84.681 (17)	O1—Fe1—K1	77.36 (12)
O6—Al1—N2	88.74 (5)	O2—K1—O6	109.26 (2)	O5—Fe1—O10	102.8 (4)
O6—Al1—C1	110.35 (5)	O2—K1—O3	57.22 (2)	O5—Fe1—O1	84.9 (3)
N2—Al1—N1	79.17 (5)	O6—K1—Al1	29.503 (16)	O5—Fe1—N2	166.7 (4)
C1—Al1—N1	104.73 (5)	O6—K1—O3	121.05 (2)	O5—Fe1—N1	90.3 (3)
C1—Al1—N2	99.41 (6)	O5—K1—Al1	83.008 (17)	O5—Fe1—K1	51.3 (2)
C3—N1—Al1	124.25 (9)	O5—K1—O3	99.35 (2)	O4—Fe1—O10	93.3 (3)
N1—C3—C4	123.74 (12)	O8—K1—Al1	62.538 (18)	O4—Fe1—O1	172.25 (18)
C4—C3—H3	118.1	O3—K1—Al1	120.450 (18)	O4—Fe1—O5	87.4 (3)
		O9—Al1—K1	108.80 (2)	O4—Fe1—N2	95.3 (3)
		O9—Al1—N2	84.99 (4)	O4—Fe1—N1	90.8 (3)

Table S9 Summary of crystallographic refinement data for complexes Al(III)-Et, Al(III)K(I) (1) and Fe(III)K(I) (2).

Complex	Al(III)-Et	Al(III)K(I)	Fe(III)K(I)
**Local Code**	**(007ke23)**	**(022ke23)**	**(006ke23-ke001)**
**CCDC Deposition Number**	2343091	2343090	2343089
**Crystal Data**			
Chemical formula	$C_{24}H_{31}AlN_2O_6$	$C_{26}H_{32}AlKN_2O_{10}$	$C_{26}H_{32}FeKN_2O_{10}$·[+solvents]
M_r	470.49	598.61	627.48
Crystal system, space group	Triclinic, *P*-1	Triclinic, *P*-1	Monoclinic, *C*2/*c*
Temperature (K)	150 ± 2	100 ± 2	150 ± 2
α, *β*, *γ* (Å)	10.1572 (5), 11.0501 (7), 11.5206 (6)	8.6851 (1), 9.0841 (2), 19.2955 (3)	29.1407 (5), 13.4808 (7), 14.6156 (3)
α, *β*, *γ* (°)	99.012 (5), 104.783 (5), 102.717 (5)	80.118 (1), 81.438 (1), 63.549 (2)	100.621 (2)
V (Å^3)	1188.13 (12)	1338.22 (4)	5643.2 (3)
Z	2	2	8
Radiation type	Cu *K*α	Cu *K*α	Cu *K*α
μ (mm^{-1})	1.11	2.60	6.12
Crystal size (mm)	0.30 × 0.17 × 0.08	0.3 × 0.08 × 0.02	0.14 × 0.10 × 0.06
**Data collection**			
Diffractometer	SuperNova, Dual, Cu at home/near, Atlas	XtaLAB Synergy R, DW system, HyPix-Arc 150	SuperNova, Dual, Cu at home/near, Atlas
Absorption correction	Gaussian *CrysAlis PRO* 1.171.40.53 (Rigaku Oxford Diffraction, 2019) Numerical absorption correction based on gaussian integration over a multifaceted crystal model Empirical absorption correction using spherical harmonics, implemented in SCALE3 ABSPACK scaling algorithm.	Multi-scan *CrysAlis PRO* 1.171.43.95a (Rigaku Oxford Diffraction, 2023) Empirical absorption correction using spherical harmonics, implemented in SCALE3 ABSPACK scaling algorithm.	Multi-scan *CrysAlis PRO* 1.171.42.72a (Rigaku Oxford Diffraction, 2022) Empirical absorption correction using spherical harmonics, implemented in SCALE3 ABSPACK scaling algorithm.
T_{min}, T_{max}	0.484, 1.000	0.722, 1.000	0.918, 1.000
No. of measured, independent and observed [*I* > 2s(*I*)] reflections	12258, 4892, 4158	50000, 5466, 5372	27706, 5852, 4260
R_{int}	0.027	0.021	0.045
(sin q/l)$_{max}$ (Å^{-1})	0.630	0.629	0.630
**Refinement**			
$R[F^2 > 2s(F^2)]$, $wR(F^2)$, S	0.034, 0.096, 1.04	0.024, 0.067, 1.07	0.047, 0.134, 1.03
No. of reflections	4892	5466	5852
No. of parameters	301	365	606
No. of restraints	0	0	1949
H-atom treatment	H-atom parameters constrained	H-atom parameters constrained	H-atom parameters constrained
Dρ$_{max}$, Dρ$_{min}$ (e Å^{-3})	0.27, -0.23	0.27, -0.23	0.35, -0.36

Computer programs: *CrysAlis PRO* 1.171.43.95a (Rigaku OD, 2023)[5], *SHELXT* 2018/2 (Sheldrick, 2018)[6], *SHELXL* 2018/3 (Sheldrick, 2015)[7, 8], Olex2 1.5 (Dolomanov *et al.*, 2009)[9]

References

(1) Darensbourg, D. J.; Billodeaux, D. R. Aluminum Salen Complexes and Tetrabutylammonium Salts: A Binary Catalytic System for Production of Polycarbonates from CO2 and Cyclohexene Oxide. *Inorganic Chemistry* **2005**, *44* (5), 1433-1442. DOI: 10.1021/ic048508g.

(2) Nishioka, K.; Goto, H.; Sugimoto, H. Dual Catalyst System for Asymmetric Alternating Copolymerization of Carbon Dioxide and Cyclohexene Oxide with Chiral Aluminum Complexes: Lewis Base as Catalyst Activator and Lewis Acid as Monomer Activator. *Macromolecules* **2012**, *45* (20), 8172-8192. DOI: 10.1021/ma301696d.

(3) Deng, J.; Ratanasak, M.; Sako, Y.; Tokuda, H.; Maeda, C.; Hasegawa, J.-y.; Nozaki, K.; Ema, T. Aluminum porphyrins with quaternary ammonium halides as catalysts for copolymerization of cyclohexene oxide and CO2: metal–ligand cooperative catalysis. *Chemical Science* **2020**, *11* (22), 5669-5675, 10.1039/D0SC01609H. DOI: 10.1039/D0SC01609H.

(4) Diment, W. T.; Gregory, G. L.; Kerr, R. W. F.; Phanopoulos, A.; Buchard, A.; Williams, C. K. Catalytic Synergy Using Al(III) and Group 1 Metals to Accelerate Epoxide and Anhydride Ring-Opening Copolymerizations. *ACS Catalysis* **2021**, *11* (20), 12532-12542. DOI: 10.1021/acscatal.1c04020.

(5) *CrysAlis PRO*; Rigaku Oxford Diffraction Ltd, Yarnton, Oxfordshire, England.: 2020. (accessed.

(6) Sheldrick, I. U. a. G. M. An introduction to experimental phasing of macromolecules illustrated by SHELX; new autotracing features. *Acta Cryst.* **2018**, (D74), 106-116.

(7) Sheldrick, G. M. SHELXT – Integrated space-group and crystal-structure determination. *Acta Cryst. A* **2014**, *71*, 3-8. DOI: https://doi.org/10.1107/S2053273314026370.

(8) Sheldrick, G. M. Crystal structure refinement with SHELXL. *Acta Cryst. C* **2015**, *71*, 3-8.

(9) Dolomanov, O. V., Bourhis, L. J., Gildea, R. J., Howard, J. A. K. & Puschmann, H. . OLEX2: a complete structure solution, refinement and analysis program. *J. Appl. Cryst.* **2009**, *41*, 339-341.
